# Megafauna of the UKSRL exploration contract area and eastern Clarion-Clipperton Zone in the Pacific Ocean: Annelida, Arthropoda, Bryozoa, Chordata, Ctenophora, Mollusca

**DOI:** 10.3897/BDJ.5.e14598

**Published:** 2017-08-14

**Authors:** Diva J Amon, Amanda F Ziegler, Jeffrey C Drazen, Andrei V Grischenko, Astrid B Leitner, Dhugal J Lindsay, Janet R Voight, Mary K Wicksten, Craig M Young, Craig R Smith

**Affiliations:** 1 University of Hawaii, Honolulu, United States of America; 2 Perm State National Research University, Perm, Russia; 3 Japan Agency for Marine-Earth Science and Technology, Yokosuka, Japan; 4 The Field Museum, Chicago, United States of America; 5 Texas A&M University, College Station, United States of America; 6 Oregon Institute of Marine Biology, University of Oregon, Charleston, United States of America

**Keywords:** deep-sea mining, polymetallic nodule, Clarion-Clipperton Zone, megafauna, atlas

## Abstract

**Background:**

There is growing interest in mining polymetallic nodules from the abyssal Clarion-Clipperton Zone (CCZ) in the tropical Pacific Ocean. Despite having been the focus of environmental studies for decades, the benthic megafauna of the CCZ remain poorly known. To predict and manage the environmental impacts of mining in the CCZ, baseline knowledge of the megafauna is essential. The ABYSSLINE Project has conducted benthic biological baseline surveys in the UK Seabed Resources Ltd polymetallic-nodule exploration contract area (UK-1). Prior to ABYSSLINE research cruises in 2013 and 2015, no biological studies had been done in this area of the eastern CCZ.

**New information:**

Using a Remotely Operated Vehicle and Autonomous Underwater Vehicle (as well as several other pieces of equipment), the megafauna within the UK Seabed Resources Ltd exploration contract area (UK-1) and at a site ~250 km east of the UK-1 area were surveyed, allowing us to make the first estimates of megafaunal morphospecies richness from the imagery collected. Here, we present an atlas of the abyssal annelid, arthropod, bryozoan, chordate, ctenophore and molluscan megafauna observed and collected during the ABYSSLINE cruises to the UK-1 polymetallic-nodule exploration contract area in the CCZ. There appear to be at least 55 distinct morphospecies (8 Annelida, 12 Arthropoda, 4 Bryozoa, 22 Chordata, 5 Ctenophora, and 4 Mollusca) identified mostly by morphology but also using molecular barcoding for a limited number of animals that were collected. This atlas will aid the synthesis of megafaunal presence/absence data collected by contractors, scientists and other stakeholders undertaking work in the CCZ, ultimately helping to decipher the biogeography of the megafauna in this threatened habitat.

## Introduction

**Figure 1.**
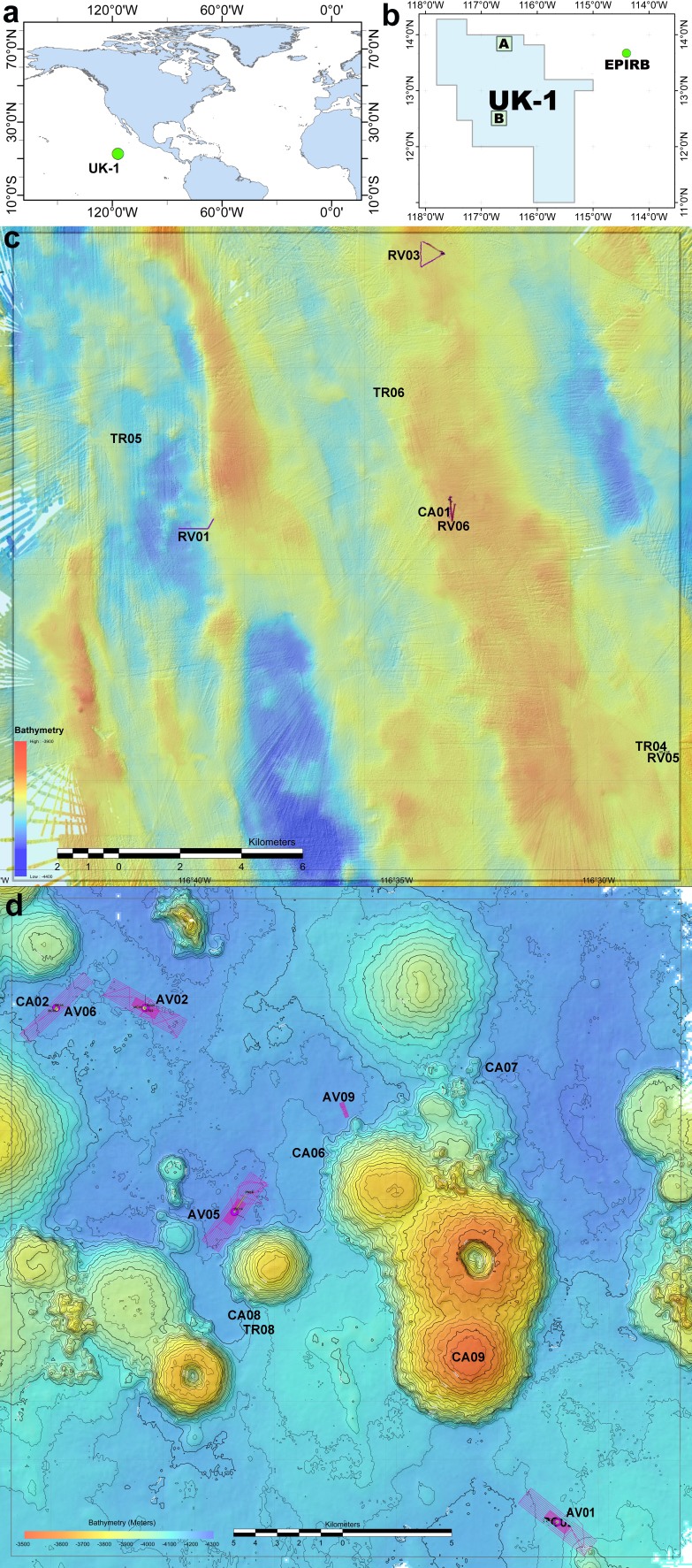
Locations of megafaunal surveys during the ABYSSLINE cruises, AB01 and AB02, in the Clarion-Clipperton Zone. (a) The location of the UK Seabed Resources Ltd exploration contract area (UK-1) in the eastern Pacific Ocean. (b) The locations of the 30x30-km survey areas, UK-1 Stratum A and UK-1 Stratum B, in relation to the UK-1 exploration contract area and the AB01 ROV dive site, EPIRB, which was approximately 250 km east of the UK-1 contract area. (c) The locations of ROV dives within UK-1 Stratum A, indicated by purple tracklines labelled with the dive number (e.g. RV01). Stations where imagery was collected with a baited camera (CA01) and samples collected with a baited trap (TR04, TR05, TR06) are also indicated. (d) The locations of AUV dives within UK-1 Stratum B, indicated by purple tracklines labelled with the dive number (e.g. AV01). Stations where imagery was collected with a baited camera (CA02, CA06, CA07, CA08, CA09) and samples collected with a baited trap (TR08) are also indicated. All maps were created by Seafloor Investigations Ltd for the ABYSSLINE Project using ArcGIS software (https://www.arcgis.com/features/).

The Clarion-Clipperton Zone (CCZ) is an abyssal region of the tropical eastern Pacific Ocean where deep-sea mining may take place in the near future (Ramirez-Llodra et al. 2011, Wedding et al. 2013; Fig. 1). High-grade polymetallic nodules, which could provide a commercial source of copper, cobalt, nickel, and manganese (among other metals), are abundant in this six million km^2^ region that lies in Areas Beyond National Jurisdiction (ABNJ), and thus falls under the legal mandate of the International Seabed Authority (ISA) (Wedding et al. 2013). Thus far, 16 exploration leases (each up to 75,000 km^2^ in area) have been granted by the ISA in the CCZ, with those for exploitation expected to soon follow (https://www.isa.org.jm/).

The ABYSSLINE (ABYSSal BaseLINE) Project was designed to undertake benthic biological baseline studies in accordance with ISA environmental guidelines within the UK Seabed Resources Ltd (UKSRL) exploration contract area (UK-1) (Amon et al. 2016). The UK-1 exploration contract area is one of the easternmost contract areas in the CCZ and encompasses ~58,000 km^2^ of seafloor (Fig. 1). The ABYSSLINE Project was led by scientists from the University of Hawai’i at Mānoa (USA), and included scientists from Hawai’i Pacific University (USA), the Natural History Museum, London (UK), the National Oceanography Centre, Southampton (UK), Senckenberg Gesellschaft für Naturforschung (Germany), Uni Research (Norway), and the International Research Institute of Stavanger (Norway). The ABYSSLINE Project aimed to evaluate baseline conditions of community structure and biodiversity for megafauna, macrofauna, meiofauna and microbes within the UK-1 contract area and across the CCZ (Amon et al. 2016, Amon et al. 2016, Amon et al. 2017, Dahlgren et al. 2016, Glover et al. 2016, Glover et al. 2015, Leitner et al. 2017, Shulse et al. 2016). No faunal studies had been undertaken in the UK-1 contract area prior to licensing by the ISA in 2013 and the commencement of the ABYSSLINE Project.

It is expected that nodule mining will drastically alter this unique deep-sea habitat with recovery expected to be slow (Amon et al. 2016, Jones et al. 2017, Oebius et al. 2001, Ramirez-Llodra et al. 2011, Vanreusel et al. 2016) and yet, despite increases in technology and the number of expeditions to the area, very little is known about the ecology and biogeography of the fauna inhabiting the region (Amon et al. 2016, Amon et al. 2016, Amon et al. 2017, Bluhm and Gebruk 1999, Dahlgren et al. 2016, Foell and Pawson 1986, Glover et al. 2016, Jones et al. 2017, Leitner et al. 2017, Martinez-Arbizu et al. 2013, Pawson 1983, Pawson and Foell 1986, Roux 2004, Roux and Pawson 1999, Shulse et al. 2016, Tilot 2006, Vanreusel et al. 2016, Vecchione 2016, Wang and Lu 2002). The megafauna constitute an important component of the biodiversity in the abyssal deep sea and play a significant role in deep-sea ecosystem function (Amon et al. 2016, Amon et al. 2017, Amon et al. 2016, Leitner et al. 2017, Smith et al. 2008, Vanreusel et al. 2016). Amon et al. 2016 could not locate a single megafauna record for the UK-1 exploration contract area in OBIS or elsewhere. This is likely the result of a lack of sampling, complete taxonomic identification of specimens and/or ensuring data are publicly available, especially as an abundant and diverse megafauna is already known from the CCZ from photographic and video surveys (Amon et al. 2016, Amon et al. 2017, Amon et al. 2016, Dahlgren et al. 2016, Foell and Pawson 1986, Glover et al. 2016, Leitner et al. 2017, Martinez-Arbizu et al. 2013, Pawson 1983, Pawson and Foell 1986, Tilot 2006, Vanreusel et al. 2016, Vecchione 2016, Wang and Lu 2002). To predict and manage the environmental impacts of mining in the CCZ and within the UK-1 exploration contract area, baseline knowledge of the megafauna is essential; in addition, it will allow for a future complete taxonomic and biogeographic synthesis of the fauna of the CCZ (Wedding et al. 2015).

Here, we present the second section (Annelida, Arthropoda, Bryozoa, Chordata, Ctenophora, and Mollusca) of an anticipated four-section image atlas of benthic megafauna that inhabit the UK-1 exploration contract area based on ROV and AUV surveys and samples collected during two cruises of the ABYSSLINE project. The first in this series (Echinodermata) has already been published (Amon et al. 2017). These two sections will be supported by Cnidaria and Porifera sections in the near future. This atlas was crucial during the ABYSSLINE quantitative megafaunal analyses (Amon et al. 2016) and we hope that it will help standardize putative morphospecies and be useful to other scientists and stakeholders undertaking research in the CCZ in the future.

## Materials and methods

The UKSRL exploration contract area (UK-1) is located in the eastern CCZ in the Pacific Ocean (Fig. 1). There were two ABYSSLINE research cruises to the UK-1 exploration contract area: the AB01 or MV1313 cruise on the R/V *Melville* from 3 to 27 October 2013, and the AB02 or TN319 cruise on the R/V *Thompson* from 12 February to 25 March 2015. The AB01 cruise focused on a 30x30-km stratum (UK-1 Stratum A) centered at 13°49′ N, 116°36′ W in the northern portion of the UK-1 contract area (Fig. 1). During the AB01 cruise, multibeam bathymetric surveys indicated an abyssal seafloor characterized by ridges and valleys running from NNW to SSE at 3900–4400 m. The commercial Remotely Operated Vehicle (ROV) *Remora III*, operated by Phoenix International Holdings, performed video surveys and sample collections at four randomly-located sites within UK-1 Stratum A in the UK-1 contract area. Additionally, surveys were done ~250 km to the east of the UK-1 contract area, at a site called "EPIRB" centered at 13°40′ N, 114°24′ W (Fig. 1). Work at the EPIRB site was dictated by an emergency response to an Emergency Position Indicating Radio Beacon (EPIRB) distress signal and, although unplanned, provided a useful broader context for our study.

The ROV was equipped with two manipulators, four ROS QLEDIII lights, one 1Cam Alpha Component high-definition downward-looking "science" video camera (1080p video and 24.1 megapixel stills) and one standard-definition forward-looking “pilot” video camera. During surveys, the vehicle had substantial difficulty maintaining constant altitude, direction and velocity over the seabed, thereby limiting both the usable imagery and specimens collected.

The AB02 cruise focused on a 30x30-km stratum (UK-1 Stratum B) centered at 12°28' N, 116°36' W in the central portion of the UK-1 exploration contract area (Fig. 1). During the AB02 cruise, multibeam bathymetric surveys indicated an abyssal seafloor dominated by numerous high-relief volcanic seamounts between 3500-4300 m (Fig. 1). The Autonomous Underwater Vehicle (AUV) *REMUS 6000*, operated by Woods Hole Oceanographic Institution, performed image surveys at five randomly-located sites within UK-1 Stratum B (Fig. 1). The AUV was equipped with four ROS QLEDIII lights, and one Prosilica GT3400 high-definition downward-looking still camera (9 megapixel stills).

During both AB01 and AB02, two baited camera systems were also used to collect video of scavenging arthropods and fishes at random locations in both the UK-1 Stratum A and UK-1 Stratum B (Leitner et al. 2017).

### Sample collection

The ROV was the primary tool used to collect specimens on the AB01 cruise, however due to significant difficulties, few megafauna were successfully sampled (Amon et al. 2016). Additionally, scavenging arthropods and fishes were captured in a baited trap during both AB01 and AB02 (Leitner et al. 2017). Once the respective sampling equipment was on deck, megafauna were quickly transferred to containers of chilled seawater, photographed, and a tissue subsample taken for DNA analyses. DNA samples were preserved in 80% ethanol and the remainder of the animal was preserved in buffered 4% formalin-seawater solution or 95% ethanol, depending on the taxon. On board, all collected specimens were also imaged, with the resulting images included in this manuscript. After the cruise, morphological samples were sent to taxonomic experts for identification and all specimens sequenced for a range of DNA markers at the Natural History Museum, London, with tissue samples subsequently archived and made openly available for future taxonomic work (Dahlgren et al. 2016, Glover et al. 2015, Glover et al. 2016). All collected specimens were used for taxonomic identifications including ground-truthing identifications based on images.

### Megafaunal image surveys and analyes

All imagery from both "pilot" and "science" cameras on the ROV (covering roughly 8,000 m^2^) collected during AB01 was used during the creation of this atlas (Amon et al. 2016, Amon et al. 2017). All imagery from the AUV (27,178 images covering roughly 500,000 m^2^) collected during AB02 was also used, although the majority of these images (>20,000 images) were at too high an altitude (>6 m) for megafauna to be resolved and identified. All video from both cameras on the ROV, as well as from the AUV, were viewed multiple times and frames archived of each identifiable megafaunal morphotype or morphospecies.

The criteria used for selection of megafaunal morphospecies during AB01 was that individuals were >2 cm in maximum dimension and that there was sufficient detail to identify them to a putative "species-level" morphotype (Amon et al. 2016). The AUV imagery collected during AB02 was lower resolution forcing the criteria to be modified to megafauna >5 cm only being included. Morphospecies that could not be identified to species but appeared morphologically distinct were assigned a unique informal species name (e.g. Polychaeta morphospecies 1). These were identified by taxonomic experts or by using the "Atlas of Abyssal Megafauna Morphotypes of the Clarion-Clipperton Fracture Zone" created for the ISA (http://ccfzatlas.com/)(Foell and Pawson 1986, Martinez-Arbizu et al. 2013, Tilot 2006). Morphospecies from this study that matched morphotypes listed in the "Atlas of Abyssal Megafauna Morphotypes of the Clarion-Clipperton Fracture Zone (Martinez-Arbizu et al. 2013)" have had a section titled 'Nomenclature' added to their data, which includes their identification from the online atlas. This is in an effort to provide coherence between these CCZ atlases. For morphospecies that were morphologically similar to a well-defined species name, we use the open nomenclature expression "cf.", although a precautionary approach was taken.

This process estimated the number of annelid, arthropod, bryozoan, chordate, ctenophore and mollusc megafauna morphospecies in the UK-1 contract area and eastern CCZ, and will aid in delimiting species ranges in the CCZ. A concise list of morphospecies and their respective locations can be found in the Suppl. material 1. However, since the majority of the morphospecies were not collected, it is impossible to confirm species identities in most cases or undertake systematic studies on this fauna (Amon et al. 2016, Amon et al. 2017).

## Checklists

### Annelids of the UKSRL exploration contract area (UK-1) and the eastern Clarion-Clipperton Zone

#### Annelida

Lamarck, 1809

#### Polychaeta

Grube, 1850

#### cf. Polychaeta morphospecies

##### Materials

- **Type status:** Other material. **Occurrence:** recordedBy: Diva J. Amon, Amanda F. Ziegler; individualCount: 1; lifeStage: Adult; behavior: On seafloor; occurrenceStatus: present; preparations: Imaged only; associatedReferences: Amon DJ, Ziegler AF, Dahlgren TG, Glover AG, Goineau A, Gooday AJ, Wiklund H, Smith CR. Insights into the abundance and diversity of abyssal megafauna in a polymetallic-nodule region in the eastern Clarion-Clipperton Zone. Scientific Reports. 2016;6. doi: 10.1038/srep30492; **Taxon:** taxonConceptID: cf. Polychaeta morphospecies; scientificName: Polychaeta sp.; kingdom: Animalia; phylum: Annelida; class: Polychaeta; taxonRank: class; scientificNameAuthorship: Grube, 1850; **Location:** waterBody: Pacific Ocean; stateProvince: Clarion-Clipperton Zone; locality: UK Seabed Resources Ltd exploration contract area (UK-1); verbatimLocality: UK-1 Stratum A; maximumDepthInMeters: 4035; locationRemarks: RV Melville Cruise MV1313; decimalLatitude: 13.8637; decimalLongitude: -116.5461; geodeticDatum: WGS84; coordinateUncertaintyInMeters: 25; **Identification:** identifiedBy: Adrian Glover, Helena Wiklund, Diva J. Amon, Amanda Ziegler; dateIdentified: 2014; identificationRemarks: Identified only from imagery; identificationQualifier: cf.; **Event:** samplingProtocol: Remotely Operated Vehicle; eventDate: 2013-10-21; eventTime: 1:09; habitat: Abyssal polymetallic-nodule field; fieldNumber: Dive 6 (RV06); **Record Level:** language: en; institutionCode: UHM; datasetName: ABYSSLINE; basisOfRecord: HumanObservation

##### Notes

Seen on seafloor. No additional distinguishing features to place it beyond class Polychaeta.

Fig. 2

#### Phyllodocida

Dales, 1962

#### Polynoidae

Kinberg, 1856

#### cf. Polynoidae morphospecies

##### Materials

- **Type status:** Other material. **Occurrence:** recordedBy: Diva J. Amon, Amanda F. Ziegler; individualCount: 1; lifeStage: Adult; behavior: Swimming; occurrenceStatus: present; preparations: Imaged only; **Taxon:** taxonConceptID: cf. Polynoidae morphospecies; scientificName: Polynoidae sp.; kingdom: Animalia; phylum: Annelida; class: Polychaeta; order: Phyllodocida; family: Polynoidae; taxonRank: family; scientificNameAuthorship: Kinberg, 1856; **Location:** waterBody: Pacific Ocean; stateProvince: Clarion-Clipperton Zone; locality: UK Seabed Resources Ltd exploration contract area (UK-1); verbatimLocality: UK-1 Stratum B; maximumDepthInMeters: 4222; locationRemarks: RV Thompson Cruise TN319; decimalLatitude: 12.5849; decimalLongitude: -116.7102; geodeticDatum: WGS84; coordinateUncertaintyInMeters: 25; **Identification:** identifiedBy: Adrian Glover, Helena Wiklund, Diva J. Amon, Amanda Ziegler; dateIdentified: 2015; identificationRemarks: Identified only from imagery; identificationQualifier: cf.; **Event:** samplingProtocol: Autonomous Underwater Vehicle; eventDate: 2015-03-09; eventTime: 14:37; habitat: Abyssal polymetallic-nodule field; fieldNumber: Dive 6 (AV06); **Record Level:** language: en; institutionCode: UHM; datasetName: ABYSSLINE; basisOfRecord: HumanObservation

##### Notes

Distinct segments and thick parapodia visible. Pennate shape with body narrowing toward anterior and posterior. Often swimming near the benthos.

Fig. 3

#### Sabellida

#### Sabellidae

Latreille, 1825

#### cf. Sabellidae morphospecies 1

##### Materials

- **Type status:** Other material. **Occurrence:** recordedBy: Diva J. Amon, Amanda F. Ziegler; individualCount: 1; lifeStage: Adult; behavior: On seafloor; occurrenceStatus: present; preparations: Imaged only; associatedReferences: Amon DJ, Ziegler AF, Dahlgren TG, Glover AG, Goineau A, Gooday AJ, Wiklund H, Smith CR. Insights into the abundance and diversity of abyssal megafauna in a polymetallic-nodule region in the eastern Clarion-Clipperton Zone. Scientific Reports. 2016;6. doi: 10.1038/srep30492; **Taxon:** taxonConceptID: cf. Sabellidae morphospecies 1; scientificName: Sabellidae sp.; kingdom: Animalia; phylum: Annelida; class: Polychaeta; order: Sabellida; family: Sabellidae; taxonRank: family; scientificNameAuthorship: Latreille, 1825; **Location:** waterBody: Pacific Ocean; stateProvince: Clarion-Clipperton Zone; locality: UK Seabed Resources Ltd exploration contract area (UK-1); verbatimLocality: UK-1 Stratum A; maximumDepthInMeters: 4029; locationRemarks: RV Melville Cruise MV1313; decimalLatitude: 13.8633; decimalLongitude: -116.5464; geodeticDatum: WGS84; coordinateUncertaintyInMeters: 25; **Identification:** identifiedBy: Adrian Glover, Helena Wiklund, Diva J. Amon, Amanda Ziegler; dateIdentified: 2014; identificationRemarks: Identified only from imagery; identificationQualifier: cf.; **Event:** samplingProtocol: Remotely Operated Vehicle; eventDate: 2013-10-21; eventTime: 9:11; habitat: Abyssal polymetallic-nodule field; fieldNumber: Dive 6 (RV06); **Record Level:** language: en; institutionCode: UHM; datasetName: ABYSSLINE; basisOfRecord: HumanObservation
- **Type status:** Other material. **Occurrence:** recordedBy: Diva J. Amon, Amanda F. Ziegler; individualCount: 1; lifeStage: Adult; behavior: On seafloor; occurrenceStatus: present; preparations: Imaged only; associatedReferences: Amon DJ, Ziegler AF, Dahlgren TG, Glover AG, Goineau A, Gooday AJ, Wiklund H, Smith CR. Insights into the abundance and diversity of abyssal megafauna in a polymetallic-nodule region in the eastern Clarion-Clipperton Zone. Scientific Reports. 2016;6. doi: 10.1038/srep30492; **Taxon:** taxonConceptID: cf. Sabellidae morphospecies 1; scientificName: Sabellidae sp.; kingdom: Animalia; phylum: Annelida; class: Polychaeta; order: Sabellida; family: Sabellidae; taxonRank: family; scientificNameAuthorship: Latreille, 1825; **Location:** waterBody: Pacific Ocean; stateProvince: Clarion-Clipperton Zone; locality: Eastern Clarion-Clipperton Zone; verbatimLocality: Site EPIRB; maximumDepthInMeters: 3934; locationRemarks: RV Melville Cruise MV1313; decimalLatitude: 13.6794; decimalLongitude: -114.4105; geodeticDatum: WGS84; coordinateUncertaintyInMeters: 25; **Identification:** identifiedBy: Adrian Glover, Helena Wiklund, Diva J. Amon, Amanda Ziegler; dateIdentified: 2014; identificationRemarks: Identified only from imagery; identificationQualifier: cf.; **Event:** samplingProtocol: Remotely Operated Vehicle; eventDate: 2013-10-23; eventTime: 12:28; habitat: Abyssal polymetallic-nodule field; fieldNumber: Dive 7 (RV07); **Record Level:** language: en; institutionCode: UHM; datasetName: ABYSSLINE; basisOfRecord: HumanObservation

##### Notes

Long, straight, and smooth tube emerging from seafloor with conical whorl of feathery tentacles at anterior.

Fig. 4

#### cf. Sabellidae morphospecies 2

##### Materials

- **Type status:** Other material. **Occurrence:** recordedBy: Diva J. Amon, Amanda F. Ziegler; individualCount: 1; lifeStage: Adult; behavior: On nodule; occurrenceStatus: present; preparations: Imaged only; associatedReferences: Amon DJ, Ziegler AF, Dahlgren TG, Glover AG, Goineau A, Gooday AJ, Wiklund H, Smith CR. Insights into the abundance and diversity of abyssal megafauna in a polymetallic-nodule region in the eastern Clarion-Clipperton Zone. Scientific Reports. 2016;6. doi: 10.1038/srep30492; **Taxon:** taxonConceptID: cf. Sabellidae morphospecies 2; scientificName: Sabellidae sp.; kingdom: Animalia; phylum: Annelida; class: Polychaeta; order: Sabellida; family: Sabellidae; taxonRank: family; scientificNameAuthorship: Latreille, 1825; **Location:** waterBody: Pacific Ocean; stateProvince: Clarion-Clipperton Zone; locality: Eastern Clarion-Clipperton Zone; verbatimLocality: Site EPIRB; maximumDepthInMeters: 3950; locationRemarks: RV Melville Cruise MV1313; decimalLatitude: 13.6794; decimalLongitude: -114.4142; geodeticDatum: WGS84; coordinateUncertaintyInMeters: 25; **Identification:** identifiedBy: Adrian Glover, Helena Wiklund, Diva J. Amon, Amanda Ziegler; dateIdentified: 2014; identificationRemarks: Identified only from imagery; identificationQualifier: cf.; **Event:** samplingProtocol: Remotely Operated Vehicle; eventDate: 2013-10-23; eventTime: 9:51; habitat: Abyssal polymetallic-nodule field; fieldNumber: Dive 7 (RV07); **Record Level:** language: en; institutionCode: UHM; datasetName: ABYSSLINE; basisOfRecord: HumanObservation

##### Notes

Thick tube seen protruding from sediment or attached to hard substrate. Whorl of feathery tentacles visible at anterior.

Fig. 5

#### Serpulidae

Rafinesque, 1815

#### cf. Serpulidae morphospecies 1

##### Materials

- **Type status:** Other material. **Occurrence:** recordedBy: Diva J. Amon, Amanda F. Ziegler; individualCount: 1; lifeStage: Adult; behavior: On nodule; occurrenceStatus: present; preparations: Imaged only; associatedReferences: Amon DJ, Ziegler AF, Dahlgren TG, Glover AG, Goineau A, Gooday AJ, Wiklund H, Smith CR. Insights into the abundance and diversity of abyssal megafauna in a polymetallic-nodule region in the eastern Clarion-Clipperton Zone. Scientific Reports. 2016;6. doi: 10.1038/srep30492; **Taxon:** taxonConceptID: cf. Serpulidae morphospecies 1; scientificName: Serpulidae sp.; kingdom: Animalia; phylum: Annelida; class: Polychaeta; order: Sabellida; family: Serpulidae; taxonRank: family; scientificNameAuthorship: Rafinesque, 1815; **Location:** waterBody: Pacific Ocean; stateProvince: Clarion-Clipperton Zone; locality: UK Seabed Resources Ltd exploration contract area (UK-1); verbatimLocality: UK-1 Stratum A; maximumDepthInMeters: 4024; locationRemarks: RV Melville Cruise MV1313; decimalLatitude: 13.8561; decimalLongitude: -116.5476; geodeticDatum: WGS84; coordinateUncertaintyInMeters: 25; **Identification:** identifiedBy: Adrian Glover, Helena Wiklund, Diva J. Amon, Amanda Ziegler; dateIdentified: 2014; identificationRemarks: Identified only from imagery; identificationQualifier: cf.; **Event:** samplingProtocol: Remotely Operated Vehicle; eventDate: 2013-10-21; eventTime: 2:59; habitat: Abyssal polymetallic-nodule field; fieldNumber: Dive 6 (RV06); **Record Level:** language: en; institutionCode: UHM; datasetName: ABYSSLINE; basisOfRecord: HumanObservation

##### Notes

White calcareous tube attached flat to hard substrate with feathery tentacles visible protruding from anterior end of tube.

Fig. 6

#### cf. Serpulidae morphospecies 2

##### Materials

- **Type status:** Other material. **Occurrence:** recordedBy: Diva J. Amon, Amanda F. Ziegler; individualCount: 1; lifeStage: Adult; behavior: On nodule; occurrenceStatus: present; preparations: Imaged only; associatedReferences: Amon DJ, Ziegler AF, Dahlgren TG, Glover AG, Goineau A, Gooday AJ, Wiklund H, Smith CR. Insights into the abundance and diversity of abyssal megafauna in a polymetallic-nodule region in the eastern Clarion-Clipperton Zone. Scientific Reports. 2016;6. doi: 10.1038/srep30492; **Taxon:** taxonConceptID: cf. Serpulidae morphospecies 2; scientificName: Serpulidae sp.; kingdom: Animalia; phylum: Annelida; class: Polychaeta; order: Sabellida; family: Serpulidae; taxonRank: family; scientificNameAuthorship: Rafinesque, 1815; **Location:** waterBody: Pacific Ocean; stateProvince: Clarion-Clipperton Zone; locality: UK Seabed Resources Ltd exploration contract area (UK-1); verbatimLocality: UK-1 Stratum A; maximumDepthInMeters: 4109; locationRemarks: RV Melville Cruise MV1313; decimalLatitude: 13.8498; decimalLongitude: -116.6457; geodeticDatum: WGS84; coordinateUncertaintyInMeters: 25; **Identification:** identifiedBy: Adrian Glover, Helena Wiklund, Diva J. Amon, Amanda Ziegler; dateIdentified: 2014; identificationRemarks: Identified only from imagery; identificationQualifier: cf.; **Event:** samplingProtocol: Remotely Operated Vehicle; eventDate: 2013-10-10; eventTime: 12:40; habitat: Abyssal polymetallic-nodule field; fieldNumber: Dive 1 (RV01); **Record Level:** language: en; institutionCode: UHM; datasetName: ABYSSLINE; basisOfRecord: HumanObservation

##### Notes

White calcareous tube seen projecting off hard substrate into water column with feathery tentacles visible from anterior.

Fig. 7

#### cf. Serpulidae morphospecies 3

##### Materials

- **Type status:** Other material. **Occurrence:** recordedBy: Diva J. Amon, Amanda F. Ziegler; individualCount: 1; lifeStage: Adult; behavior: On seafloor; occurrenceStatus: present; preparations: Imaged only; associatedReferences: Amon DJ, Ziegler AF, Dahlgren TG, Glover AG, Goineau A, Gooday AJ, Wiklund H, Smith CR. Insights into the abundance and diversity of abyssal megafauna in a polymetallic-nodule region in the eastern Clarion-Clipperton Zone. Scientific Reports. 2016;6. doi: 10.1038/srep30492; **Taxon:** taxonConceptID: cf. Serpulidae morphospecies 3; scientificName: Serpulidae sp.; kingdom: Animalia; phylum: Annelida; class: Polychaeta; order: Sabellida; family: Serpulidae; taxonRank: family; scientificNameAuthorship: Rafinesque, 1815; **Location:** waterBody: Pacific Ocean; stateProvince: Clarion-Clipperton Zone; locality: Eastern Clarion-Clipperton Zone; verbatimLocality: Site EPIRB; maximumDepthInMeters: 3934; locationRemarks: RV Melville Cruise MV1313; decimalLatitude: 13.6791; decimalLongitude: -114.4104; geodeticDatum: WGS84; coordinateUncertaintyInMeters: 25; **Identification:** identifiedBy: Adrian Glover, Helena Wiklund, Diva J. Amon, Amanda Ziegler; dateIdentified: 2014; identificationRemarks: Identified only from imagery; identificationQualifier: cf.; **Event:** samplingProtocol: Remotely Operated Vehicle; eventDate: 2013-10-23; eventTime: 10:34; habitat: Abyssal polymetallic-nodule field; fieldNumber: Dive 7 (RV07); **Record Level:** language: en; institutionCode: UHM; datasetName: ABYSSLINE; basisOfRecord: HumanObservation

##### Notes

White calcareous tube with whorl of feathery tentacles visible from anterior end of tube seen on soft sediment.

Fig. 8

#### Terebellida

sensu Rouse & Fauchald, 1997

#### Acrocirridae

Banse, 1969

#### cf. Acrocirridae morphospecies

##### Materials

- **Type status:** Other material. **Occurrence:** recordedBy: Diva J. Amon, Amanda F. Ziegler; individualCount: 1; lifeStage: Adult; behavior: Swimming; occurrenceStatus: present; preparations: Imaged only; **Taxon:** taxonConceptID: cf. Acrocirridae morphospecies; scientificName: Acrocirridae sp.; kingdom: Animalia; phylum: Annelida; class: Polychaeta; order: Terebellida; family: Acrocirridae; taxonRank: family; scientificNameAuthorship: Banse, 1969; **Location:** waterBody: Pacific Ocean; stateProvince: Clarion-Clipperton Zone; locality: Eastern Clarion-Clipperton Zone; verbatimLocality: Site EPIRB; maximumDepthInMeters: 3915; locationRemarks: RV Melville Cruise MV1313; decimalLatitude: 13.6785; decimalLongitude: -114.4067; geodeticDatum: WGS84; coordinateUncertaintyInMeters: 25; **Identification:** identifiedBy: Adrian Glover, Helena Wiklund, Diva J. Amon, Amanda Ziegler; dateIdentified: 2014; identificationRemarks: Identified only from imagery; identificationQualifier: cf.; **Event:** samplingProtocol: Remotely Operated Vehicle; eventDate: 2013-10-23; eventTime: 11:14; habitat: Abyssal polymetallic-nodule field; fieldNumber: Dive 7 (RV07); **Record Level:** language: en; institutionCode: UHM; datasetName: ABYSSLINE; basisOfRecord: HumanObservation

##### Notes

Distinct segments visible with long, paddle-like parapodia. Often swimming near the benthos.

Fig. 9

### Arthropods of the UKSRL exploration contract area (UK-1) and the eastern Clarion-Clipperton Zone

#### Arthropoda

von Siebold, 1848

#### Malacostraca

Latreille, 1802

#### Amphipoda

Latreille, 1816

#### Eurytheneidae

Stoddart & Lowry, 2004

#### Eurythenes

Smith in Scudder, 1882

#### cf. Eurythenes morphospecies 1

##### Materials

- **Type status:** Other material. **Occurrence:** recordedBy: Jeffrey Drazen, Astrid Leitner; individualCount: 1; lifeStage: Adult; behavior: Feeding on bait; occurrenceStatus: present; preparations: Imaged only; associatedReferences: Leitner A, Neuheimer A, Donlon E, Smith CR, Drazen JC. Environmental and bathymetric influences on abyssal bait-attending communities of the Clarion Clipperton Zone. Deep Sea Research Part I: Oceanographic Research Papers. 2017. doi: 10.1016/j.dsr.2017.04.017; **Taxon:** taxonConceptID: cf. *Eurythenes* morphospecies 1; scientificName: *Eurythenes* sp.; kingdom: Animalia; phylum: Arthropoda; class: Malacostraca; order: Amphipoda; family: Eurytheneidae; genus: Eurythenes; taxonRank: genus; scientificNameAuthorship: Smith in Scudder, 1882; **Location:** waterBody: Pacific Ocean; stateProvince: Clarion-Clipperton Zone; locality: UK Seabed Resources Ltd exploration contract area (UK-1); verbatimLocality: UK-1 Stratum B; maximumDepthInMeters: 4312; locationRemarks: RV Thompson Cruise TN319; decimalLatitude: 12.5532; decimalLongitude: -116.5386; geodeticDatum: WGS84; coordinateUncertaintyInMeters: 50; **Identification:** identifiedBy: Mary Wicksten, Jeffrey Drazen, Astrid Leitner, Diva J. Amon, Amanda Ziegler; dateIdentified: 2015; identificationRemarks: Identified only from imagery; identificationQualifier: cf.; **Event:** samplingProtocol: Baited Camera; eventDate: 2015-03-06; eventTime: 19:37; habitat: Abyssal polymetallic-nodule field; fieldNumber: CA07; **Record Level:** language: en; institutionCode: UHM; datasetName: ABYSSLINE; basisOfRecord: HumanObservation

##### Notes

Colour is different so might be a different species than *E. magellanicus* but the body color in *Eurythenes* spp. varies according to the stage of the molt cycle.

Fig. 10

#### Eurythenes magellanicus

H. Milne Edwards, 1848

##### Materials

- **Type status:** Other material. **Occurrence:** catalogNumber: AB1-TR04-amph-5; recordNumber: AB1-TR04-amph-5; recordedBy: Jeffrey Drazen, Astrid Leitner; individualCount: 1; lifeStage: Adult; occurrenceStatus: present; preparations: tissue and DNA voucher stored in 80% non-denatured ethanol aqueous solution and remainder of animal preserved in 4% formaldehyde; **Taxon:** taxonConceptID: Eurythenes magellanicus; scientificName: Eurythenes magellanicus; kingdom: Animalia; phylum: Arthropoda; class: Malacostraca; order: Amphipoda; family: Eurytheneidae; genus: Eurythenes; taxonRank: species; scientificNameAuthorship: H. Milne Edwards, 1848; **Location:** waterBody: Pacific Ocean; stateProvince: Clarion-Clipperton Zone; locality: UK Seabed Resources Ltd exploration contract area (UK-1); verbatimLocality: UK-1 Stratum A; maximumDepthInMeters: 4170; locationRemarks: RV Melville Cruise MV1313; decimalLatitude: 13.7615; decimalLongitude: -116.4655; geodeticDatum: WGS84; coordinateUncertaintyInMeters: 50; **Identification:** identifiedBy: Inga Mohrbeck, Mary Wicksten, Jeffrey Drazen, Astrid Leitner, Diva J. Amon, Amanda Ziegler; dateIdentified: 2014; identificationRemarks: Identified by morphology and DNA of collected specimen; **Event:** samplingProtocol: Baited Trap; eventDate: 2013-10-17; eventTime: 7:13; habitat: Abyssal polymetallic-nodule field; fieldNumber: TR04; **Record Level:** language: en; institutionCode: UHM; datasetName: ABYSSLINE; basisOfRecord: PreservedSpecimen

##### Notes

This and other amphipods can be distinguished from "true shrimp" (Order Decapoda) by having NO carapace, sessile eyes (no stalks) and three pairs of terminal appendages (uropods). Species of *Eurythenes* are the largest deep-sea amphipods and often are attracted to bait. Notice that there are large coxal plates at the bases of the legs - these are absent in decapod shrimps.

Fig. 11

#### Podoceridae

Leach, 1814

#### cf. Podoceridae morphospecies

##### Materials

- **Type status:** Other material. **Occurrence:** recordedBy: Diva J. Amon, Amanda F. Ziegler; individualCount: 2; lifeStage: Adult; behavior: On a sponge stalk; occurrenceStatus: present; preparations: Imaged only; associatedReferences: Amon DJ, Ziegler AF, Dahlgren TG, Glover AG, Goineau A, Gooday AJ, Wiklund H, Smith CR. Insights into the abundance and diversity of abyssal megafauna in a polymetallic-nodule region in the eastern Clarion-Clipperton Zone. Scientific Reports. 2016;6. doi: 10.1038/srep30492; **Taxon:** taxonConceptID: cf. Podoceridae morphospecies; scientificName: Podoceridae sp.; kingdom: Animalia; phylum: Arthropoda; class: Malacostraca; order: Amphipoda; family: Podoceridae; taxonRank: family; scientificNameAuthorship: Leach, 1814; **Location:** waterBody: Pacific Ocean; stateProvince: Clarion-Clipperton Zone; locality: UK Seabed Resources Ltd exploration contract area (UK-1); verbatimLocality: UK-1 Stratum A; maximumDepthInMeters: 4073; locationRemarks: RV Melville Cruise MV1313; decimalLatitude: 13.7603; decimalLongitude: -116.4678; geodeticDatum: WGS84; coordinateUncertaintyInMeters: 25; **Identification:** identifiedBy: Mary Wicksten, Diva J. Amon, Amanda F. Ziegler; dateIdentified: 2014; identificationRemarks: Identified only from imagery; identificationQualifier: cf.; **Event:** samplingProtocol: Remotely Operated Vehicle; eventDate: 2013-10-18; eventTime: 2:45; habitat: Abyssal polymetallic-nodule field; fieldNumber: Dive 5 (RV05); **Record Level:** language: en; institutionCode: UHM; datasetName: ABYSSLINE; basisOfRecord: HumanObservation

##### Notes

The very large dorsal spines on the posterior part of the body are diagnostic. Often found on stalks in pairs.

Fig. 12

#### Decapoda

Latreille, 1802

#### Aristeidae

Wood-Mason in Wood-Mason & Alcock, 1891

#### Hemipenaeus

Spence Bate, 1881

#### Hemipenaeus cf. spinidorsalis

Spence Bate, 1881

##### Materials

- **Type status:** Other material. **Occurrence:** recordedBy: Jeffrey Drazen, Astrid Leitner; individualCount: 1; lifeStage: Adult; behavior: Swimming; occurrenceStatus: present; preparations: Imaged only; **Taxon:** taxonConceptID: Hemipenaeus cf. spinidorsalis; scientificName: Hemipenaeus spinidorsalis; kingdom: Animalia; phylum: Arthropoda; class: Malacostraca; order: Decapoda; family: Aristeidae; genus: Hemipenaeus; taxonRank: species; scientificNameAuthorship: Spence Bate, 1881; **Location:** waterBody: Pacific Ocean; stateProvince: Clarion-Clipperton Zone; locality: UK Seabed Resources Ltd exploration contract area (UK-1); verbatimLocality: UK-1 Stratum A; maximumDepthInMeters: 4046; locationRemarks: RV Melville Cruise MV1313; decimalLatitude: 13.8661; decimalLongitude: -116.5548; geodeticDatum: WGS84; coordinateUncertaintyInMeters: 50; **Identification:** identifiedBy: Mary Wicksten, Jeffrey Drazen, Astrid Leitner, Diva J. Amon, Amanda Ziegler; dateIdentified: 2014; identificationRemarks: Identified only from imagery; identificationQualifier: cf.; **Event:** samplingProtocol: Baited Camera; eventDate: 2013-10-09; eventTime: 12:05; habitat: Abyssal polymetallic-nodule field; fieldNumber: CA01; **Record Level:** language: en; institutionCode: UHM; datasetName: ABYSSLINE; basisOfRecord: HumanObservation
- **Type status:** Other material. **Occurrence:** recordedBy: Diva J. Amon, Amanda F. Ziegler; individualCount: 1; lifeStage: Adult; behavior: Swimming; occurrenceStatus: present; preparations: Imaged only; **Taxon:** taxonConceptID: Hemipenaeus cf. spinidorsalis; scientificName: Hemipenaeus spinidorsalis; kingdom: Animalia; phylum: Arthropoda; class: Malacostraca; order: Decapoda; family: Aristeidae; genus: Hemipenaeus; taxonRank: species; scientificNameAuthorship: Spence Bate, 1881; **Location:** waterBody: Pacific Ocean; stateProvince: Clarion-Clipperton Zone; locality: UK Seabed Resources Ltd exploration contract area (UK-1); verbatimLocality: UK-1 Stratum B; maximumDepthInMeters: 4210; locationRemarks: RV Thompson Cruise TN319; decimalLatitude: 12.5684; decimalLongitude: -116.7327; geodeticDatum: WGS84; coordinateUncertaintyInMeters: 25; **Identification:** identifiedBy: Mary Wicksten, Diva J. Amon, Amanda F. Ziegler; dateIdentified: 2015; identificationRemarks: Identified only from imagery; identificationQualifier: cf.; **Event:** samplingProtocol: Autonomous Underwater Vehicle; eventDate: 2015-03-09; eventTime: 12:17; habitat: Abyssal polymetallic-nodule field; fieldNumber: Dive 6 (AV06); **Record Level:** language: en; institutionCode: UHM; datasetName: ABYSSLINE; basisOfRecord: HumanObservation
- **Type status:** Other material. **Occurrence:** recordedBy: Diva J. Amon, Amanda F. Ziegler; individualCount: 1; lifeStage: Adult; behavior: Swimming; occurrenceStatus: present; preparations: Imaged only; **Taxon:** taxonConceptID: Hemipenaeus cf. spinidorsalis; scientificName: Hemipenaeus spinidorsalis; kingdom: Animalia; phylum: Arthropoda; class: Malacostraca; order: Decapoda; family: Aristeidae; genus: Hemipenaeus; taxonRank: species; scientificNameAuthorship: SpenceBate, 1881; **Location:** waterBody: Pacific Ocean; stateProvince: Clarion-Clipperton Zone; locality: UK Seabed Resources Ltd exploration contract area (UK-1); verbatimLocality: UK-1 Stratum B; maximumDepthInMeters: 4252; locationRemarks: RV Thompson Cruise TN319; decimalLatitude: 12.5103; decimalLongitude: -116.6390; geodeticDatum: WGS84; coordinateUncertaintyInMeters: 25; **Identification:** identifiedBy: Mary Wicksten, Diva J. Amon, Amanda F. Ziegler; dateIdentified: 2015; identificationRemarks: Identified only from imagery; identificationQualifier: cf.; **Event:** samplingProtocol: Autonomous Underwater Vehicle; eventDate: 2015-03-18; eventTime: 13:36; habitat: Abyssal polymetallic-nodule field; fieldNumber: Dive 9 (AV09); **Record Level:** language: en; institutionCode: UHM; datasetName: ABYSSLINE; basisOfRecord: HumanObservation
- **Type status:** Other material. **Occurrence:** recordedBy: Jeffrey Drazen, Astrid Leitner; individualCount: 1; lifeStage: Adult; behavior: Swimming; occurrenceStatus: present; preparations: Imaged only; **Taxon:** taxonConceptID: Hemipenaeus cf. spinidorsalis; scientificName: Hemipenaeus spinidorsalis; kingdom: Animalia; phylum: Arthropoda; class: Malacostraca; order: Decapoda; family: Aristeidae; genus: Hemipenaeus; taxonRank: species; scientificNameAuthorship: Spence Bate, 1881; **Location:** waterBody: Pacific Ocean; stateProvince: Clarion-Clipperton Zone; locality: UK Seabed Resources Ltd exploration contract area (UK-1); verbatimLocality: UK-1 Stratum B; maximumDepthInMeters: 4312; locationRemarks: RV Thompson Cruise TN319; decimalLatitude: 12.5532; decimalLongitude: -116.5386; geodeticDatum: WGS84; coordinateUncertaintyInMeters: 50; **Identification:** identifiedBy: Mary Wicksten, Jeffrey Drazen, Astrid Leitner, Diva J. Amon, Amanda Ziegler; dateIdentified: 2015; identificationRemarks: Identified only from imagery; identificationQualifier: cf.; **Event:** samplingProtocol: Baited Camera; eventDate: 2015-03-05; eventTime: 19:37; habitat: Abyssal polymetallic-nodule field; fieldNumber: CA07; **Record Level:** language: en; institutionCode: UHM; datasetName: ABYSSLINE; basisOfRecord: HumanObservation
- **Type status:** Other material. **Occurrence:** recordedBy: Jeffrey Drazen, Astrid Leitner; individualCount: 1; lifeStage: Adult; behavior: Swimming; occurrenceStatus: present; preparations: Imaged only; **Taxon:** taxonConceptID: Hemipenaeus cf. spinidorsalis; scientificName: Hemipenaeus spinidorsalis; kingdom: Animalia; phylum: Arthropoda; class: Malacostraca; order: Decapoda; family: Aristeidae; genus: Hemipenaeus; taxonRank: species; scientificNameAuthorship: Spence Bate, 1881; **Location:** waterBody: Pacific Ocean; stateProvince: Clarion-Clipperton Zone; locality: UK Seabed Resources Ltd exploration contract area (UK-1); verbatimLocality: UK-1 Stratum B; maximumDepthInMeters: 4201; locationRemarks: RV Thompson Cruise TN319; decimalLatitude: 12.3836; decimalLongitude: -116.4907; geodeticDatum: WGS84; coordinateUncertaintyInMeters: 50; **Identification:** identifiedBy: Mary Wicksten, Jeffrey Drazen, Astrid Leitner, Diva J. Amon, Amanda Ziegler; dateIdentified: 2015; identificationRemarks: Identified only from imagery; identificationQualifier: cf.; **Event:** samplingProtocol: Baited Camera; eventDate: 2013-10-19; eventTime: 17:01; habitat: Abyssal polymetallic-nodule field; fieldNumber: CA01; **Record Level:** language: en; institutionCode: UHM; datasetName: ABYSSLINE; basisOfRecord: HumanObservation

##### Notes

Red color, toothed rostrum, abdominal appendages very long and setose, antennal scale is round, not elongate-oval, able to swim readily. Likely to be *H. spinidorsalis* because this is the only species of the genus reported in this part of the Pacific.

Fig. 13

#### Cerataspis

Gray, 1828

#### Cerataspis cf. monstrosus

Gray, 1828

##### Materials

- **Type status:** Other material. **Occurrence:** recordedBy: Jeffrey Drazen, Astrid Leitner; individualCount: 1; lifeStage: Adult; behavior: Feeding on bait; occurrenceStatus: present; preparations: Imaged only; associatedReferences: Amon DJ, Ziegler AF, Dahlgren TG, Glover AG, Goineau A, Gooday AJ, Wiklund H, Smith CR. Insights into the abundance and diversity of abyssal megafauna in a polymetallic-nodule region in the eastern Clarion-Clipperton Zone. Scientific Reports. 2016;6. doi: 10.1038/srep30492; Leitner A, Neuheimer A, Donlon E, Smith CR, Drazen JC. Environmental and bathymetric influences on abyssal bait-attending communities of the Clarion Clipperton Zone. Deep Sea Research Part I: Oceanographic Research Papers. 2017. doi: 10.1016/j.dsr.2017.04.017; **Taxon:** taxonConceptID: Cerataspis cf. monstrosus; scientificName: Cerataspis monstrosus; kingdom: Animalia; phylum: Arthropoda; class: Malacostraca; order: Decapoda; family: Aristeidae; genus: Cerataspis; taxonRank: species; scientificNameAuthorship: Gray, 1828; **Location:** waterBody: Pacific Ocean; stateProvince: Clarion-Clipperton Zone; locality: UK Seabed Resources Ltd exploration contract area (UK-1); verbatimLocality: UK-1 Stratum A; maximumDepthInMeters: 4046; locationRemarks: RV Melville Cruise MV1313; decimalLatitude: 13.8661; decimalLongitude: -116.5548; geodeticDatum: WGS84; coordinateUncertaintyInMeters: 50; **Identification:** identifiedBy: Mary Wicksten, Jeffrey Drazen, Astrid Leitner, Diva J. Amon, Amanda Ziegler; dateIdentified: 2014; identificationRemarks: Identified only from imagery; identificationQualifier: cf.; **Event:** samplingProtocol: Baited Camera; eventDate: 2013-10-09; eventTime: 12:05; habitat: Abyssal polymetallic-nodule field; fieldNumber: CA01; **Record Level:** language: en; institutionCode: UHM; datasetName: ABYSSLINE; basisOfRecord: HumanObservation

##### Notes

Very long abdominal appendages, ability to swim, toothed rostrum, red color, elongate oval antennal scale. One of the largest deep-sea shrimp.

Fig. 14

#### Glyphocrangonidae

Smith, 1884

#### Glyphocrangon

A. Milne-Edwards, 1881

#### cf. Glyphocrangon morphospecies

##### Materials

- **Type status:** Other material. **Occurrence:** recordedBy: Diva J. Amon, Amanda F. Ziegler; individualCount: 1; lifeStage: Adult; behavior: On seafloor; occurrenceStatus: present; preparations: Imaged only; **Taxon:** taxonConceptID: cf. *Glyphocrangon* morphospecies; scientificName: *Glyphocrangon* sp.; kingdom: Animalia; phylum: Arthropoda; class: Malacostraca; order: Decapoda; family: Glyphocrangonidae; genus: Glyphocrangon; taxonRank: genus; scientificNameAuthorship: Milne-Edwards, 1881; **Location:** waterBody: Pacific Ocean; stateProvince: Clarion-Clipperton Zone; locality: UK Seabed Resources Ltd exploration contract area (UK-1); verbatimLocality: UK-1 Stratum B; maximumDepthInMeters: 4222; locationRemarks: RV Thompson Cruise TN319; decimalLatitude: 12.5842; decimalLongitude: -116.7045; geodeticDatum: WGS84; coordinateUncertaintyInMeters: 25; **Identification:** identifiedBy: Mary Wicksten, Diva J. Amon, Amanda F. Ziegler; dateIdentified: 2015; identificationRemarks: Identified only from imagery; identificationQualifier: cf.; **Event:** samplingProtocol: Autonomous Underwater Vehicle; eventDate: 2015-03-09; eventTime: 5:41; habitat: Abyssal polymetallic-nodule field; fieldNumber: Dive 6 (AV06); **Record Level:** language: en; institutionCode: UHM; datasetName: ABYSSLINE; basisOfRecord: HumanObservation

##### Notes

Very large round eyes, stocky body, ridged margins on abdominal somites, short legs. In lateral view, carapace bears strong ridges (carinae).

Fig. 15

#### Munidopsidae

Ortmann, 1898

#### Munidopsis

Whiteaves, 1874

#### cf. Munidopsis morphospecies

##### Materials

- **Type status:** Other material. **Occurrence:** recordedBy: Jeffrey Drazen, Astrid Leitner; individualCount: 1; lifeStage: Adult; behavior: On seafloor; occurrenceStatus: present; preparations: Imaged only; associatedReferences: Leitner A, Neuheimer A, Donlon E, Smith CR, Drazen JC. Environmental and bathymetric influences on abyssal bait-attending communities of the Clarion Clipperton Zone. Deep Sea Research Part I: Oceanographic Research Papers. 2017. doi: 10.1016/j.dsr.2017.04.017; **Taxon:** taxonConceptID: cf. *Munidopsis* morphospecies; scientificName: *Munidopsis* sp.; kingdom: Animalia; phylum: Arthropoda; class: Malacostraca; order: Decapoda; family: Munidopsidae; genus: Munidopsis; taxonRank: genus; scientificNameAuthorship: Whiteaves, 1874; **Location:** waterBody: Pacific Ocean; stateProvince: Clarion-Clipperton Zone; locality: UK Seabed Resources Ltd exploration contract area (UK-1); verbatimLocality: UK-1 Stratum B; maximumDepthInMeters: 3605; locationRemarks: RV Thompson Cruise TN319; decimalLatitude: 12.4353; decimalLongitude: -116.5533; geodeticDatum: WGS84; coordinateUncertaintyInMeters: 50; **Identification:** identifiedBy: Mary Wicksten, Jeffrey Drazen, Astrid Leitner, Diva J. Amon, Amanda Ziegler; dateIdentified: 2015; identificationRemarks: Identified only from imagery; identificationQualifier: cf.; **Event:** samplingProtocol: Baited Camera; eventDate: 2015-03-09; eventTime: 19:15; habitat: Abyssal polymetallic-nodule field; fieldNumber: CA09; **Record Level:** language: en; institutionCode: UHM; datasetName: ABYSSLINE; basisOfRecord: HumanObservation

##### Notes

Pale color, no pigment in eyes, short but strong pincers, three pair of walking legs (pereopods) on each side (not four, as would be seen in a true crab, Brachyura). Mouthparts do not cover oral field (as they usually do in true crabs). To identify the species, one would need a dorsal view.

Fig. 16

#### Nematocarcinidae

Smith, 1884

#### Nematocarcinus

A. Milne-Edwards, 1881

#### cf. Nematocarcinus morphospecies

##### Materials

- **Type status:** Other material. **Occurrence:** recordedBy: Diva J. Amon, Amanda F. Ziegler; individualCount: 1; lifeStage: Adult; behavior: On seafloor; occurrenceStatus: present; preparations: Imaged only; associatedReferences: Amon DJ, Ziegler AF, Dahlgren TG, Glover AG, Goineau A, Gooday AJ, Wiklund H, Smith CR. Insights into the abundance and diversity of abyssal megafauna in a polymetallic-nodule region in the eastern Clarion-Clipperton Zone. Scientific Reports. 2016;6. doi: 10.1038/srep30492; **Taxon:** taxonConceptID: cf. *Nematocarcinus* morphospecies; scientificName: *Nematocarcinus* sp.; kingdom: Animalia; phylum: Arthropoda; class: Malacostraca; order: Decapoda; family: Nematocarcinidae; genus: Nematocarcinus; taxonRank: genus; scientificNameAuthorship: Milne-Edwards, 1881; **Location:** waterBody: Pacific Ocean; stateProvince: Clarion-Clipperton Zone; locality: UK Seabed Resources Ltd exploration contract area (UK-1); verbatimLocality: UK-1 Stratum A; maximumDepthInMeters: 4032; locationRemarks: RV Melville Cruise MV1313; decimalLatitude: 13.8629; decimalLongitude: -116.5485; geodeticDatum: WGS84; coordinateUncertaintyInMeters: 25; **Identification:** identifiedBy: Mary Wicksten, Jeffrey Drazen, Astrid Leitner, Diva J. Amon, Amanda Ziegler; dateIdentified: 2014; identificationRemarks: Identified only from imagery; identificationQualifier: cf.; **Event:** samplingProtocol: Remotely Operated Vehicle; eventDate: 2013-10-21; eventTime: 4:59; habitat: Abyssal polymetallic-nodule field; fieldNumber: Dive 6 (RV06); **Record Level:** language: en; institutionCode: UHM; datasetName: ABYSSLINE; basisOfRecord: HumanObservation
- **Type status:** Other material. **Occurrence:** recordedBy: Diva J. Amon, Amanda F. Ziegler; individualCount: 1; lifeStage: Adult; behavior: On seafloor; occurrenceStatus: present; preparations: Imaged only; associatedReferences: Amon DJ, Ziegler AF, Dahlgren TG, Glover AG, Goineau A, Gooday AJ, Wiklund H, Smith CR. Insights into the abundance and diversity of abyssal megafauna in a polymetallic-nodule region in the eastern Clarion-Clipperton Zone. Scientific Reports. 2016;6. doi: 10.1038/srep30492; **Taxon:** taxonConceptID: cf. *Nematocarcinus* morphospecies; scientificName: *Nematocarcinus* sp.; kingdom: Animalia; phylum: Arthropoda; class: Malacostraca; order: Decapoda; family: Nematocarcinidae; genus: Nematocarcinus; taxonRank: genus; scientificNameAuthorship: Milne-Edwards, 1881; **Location:** waterBody: Pacific Ocean; stateProvince: Clarion-Clipperton Zone; locality: UK Seabed Resources Ltd exploration contract area (UK-1); verbatimLocality: UK-1 Stratum A; maximumDepthInMeters: 4021; locationRemarks: RV Melville Cruise MV1313; decimalLatitude: 13.8583; decimalLongitude: -116.5472; geodeticDatum: WGS84; coordinateUncertaintyInMeters: 25; **Identification:** identifiedBy: Mary Wicksten, Jeffrey Drazen, Astrid Leitner, Diva J. Amon, Amanda Ziegler; dateIdentified: 2014; identificationRemarks: Identified only from imagery; identificationQualifier: cf.; **Event:** samplingProtocol: Remotely Operated Vehicle; eventDate: 2013-10-21; eventTime: 2:30; habitat: Abyssal polymetallic-nodule field; fieldNumber: Dive 6 (RV06); **Record Level:** language: en; institutionCode: UHM; datasetName: ABYSSLINE; basisOfRecord: HumanObservation

##### Notes

Enormously long and slender legs and antennae, slender body, toothed rostrum (not visible in dorsal view), abdominal appendages usually not visible in dorsal view.

Fig. 17

#### Solenoceridae

Wood-Mason in Wood-Mason & Alcock, 1891

#### Hymenopenaeus

Smith, 1882

#### Hymenopenaeus cf. nereus

Faxon, 1893

##### Materials

- **Type status:** Other material. **Occurrence:** catalogNumber: AB1-TR05-shrimp-2; recordNumber: AB1-TR05-shrimp-2; recordedBy: Jeffrey Drazen, Astrid Leitner; individualCount: 2; lifeStage: Adult; occurrenceStatus: present; preparations: tissue and DNA voucher stored in 80% non-denatured ethanol aqueous solution and remainder of animal preserved in 4% formaldehyde; associatedReferences: Amon DJ, Ziegler AF, Dahlgren TG, Glover AG, Goineau A, Gooday AJ, Wiklund H, Smith CR. Insights into the abundance and diversity of abyssal megafauna in a polymetallic-nodule region in the eastern Clarion-Clipperton Zone. Scientific Reports. 2016;6. doi: 10.1038/srep30492; Leitner A, Neuheimer A, Donlon E, Smith CR, Drazen JC. Environmental and bathymetric influences on abyssal bait-attending communities of the Clarion Clipperton Zone. Deep Sea Research Part I: Oceanographic Research Papers. 2017. doi: 10.1016/j.dsr.2017.04.017; **Taxon:** taxonConceptID: Hymenopenaeus nereus; scientificName: Hymenopenaeus nereus; kingdom: Animalia; phylum: Arthropoda; class: Malacostraca; order: Decapoda; family: Solenoceridae; genus: Hymenopeneaus; taxonRank: species; scientificNameAuthorship: Faxon, 1893; **Location:** waterBody: Pacific Ocean; stateProvince: Clarion-Clipperton Zone; locality: UK Seabed Resources Ltd exploration contract area (UK-1); verbatimLocality: UK-1 Stratum A; maximumDepthInMeters: 4156; locationRemarks: RV Melville Cruise MV1313; decimalLatitude: 13.8885; decimalLongitude: -116.6824; geodeticDatum: WGS84; coordinateUncertaintyInMeters: 50; **Identification:** identifiedBy: Mary Wicksten, Jeffrey Drazen, Astrid Leitner, Diva J. Amon, Amanda Ziegler; dateIdentified: 2014; identificationRemarks: Identified by morphology of collected specimen; **Event:** samplingProtocol: Baited Trap; eventDate: 2013-10-19; eventTime: 4:40; habitat: Abyssal polymetallic-nodule field; fieldNumber: TR05; **Record Level:** language: en; institutionCode: UHM; datasetName: ABYSSLINE; basisOfRecord: PreservedSpecimen
- **Type status:** Other material. **Occurrence:** recordedBy: Diva J. Amon, Amanda F. Ziegler; individualCount: 1; lifeStage: Adult; behavior: On seafloor; occurrenceStatus: present; preparations: Imaged only; associatedReferences: Amon DJ, Ziegler AF, Dahlgren TG, Glover AG, Goineau A, Gooday AJ, Wiklund H, Smith CR. Insights into the abundance and diversity of abyssal megafauna in a polymetallic-nodule region in the eastern Clarion-Clipperton Zone. Scientific Reports. 2016;6. doi: 10.1038/srep30492; Leitner A, Neuheimer A, Donlon E, Smith CR, Drazen JC. Environmental and bathymetric influences on abyssal bait-attending communities of the Clarion Clipperton Zone. Deep Sea Research Part I: Oceanographic Research Papers. 2017. doi: 10.1016/j.dsr.2017.04.017; **Taxon:** taxonConceptID: Hymenopenaeus cf. nereus; scientificName: Hymenopenaeus nereus; kingdom: Animalia; phylum: Arthropoda; class: Malacostraca; order: Decapoda; family: Solenoceridae; genus: Hymenopeneaus; taxonRank: species; scientificNameAuthorship: Faxon, 1893; **Location:** waterBody: Pacific Ocean; stateProvince: Clarion-Clipperton Zone; locality: UK Seabed Resources Ltd exploration contract area (UK-1); verbatimLocality: UK-1 Stratum A; maximumDepthInMeters: 4071; locationRemarks: RV Melville Cruise MV1313; decimalLatitude: 13.7604; decimalLongitude: -116.4679; geodeticDatum: WGS84; coordinateUncertaintyInMeters: 25; **Identification:** identifiedBy: Mary Wicksten, Diva J. Amon, Amanda F. Ziegler; dateIdentified: 2014; identificationRemarks: Identified only from imagery; identificationQualifier: cf.; **Event:** samplingProtocol: Remotely Operated Vehicle; eventDate: 2013-10-18; eventTime: 2:21; habitat: Abyssal polymetallic-nodule field; fieldNumber: Dive 5 (RV05); **Record Level:** language: en; institutionCode: UHM; datasetName: ABYSSLINE; basisOfRecord: HumanObservation
- **Type status:** Other material. **Occurrence:** recordedBy: Diva J. Amon, Amanda F. Ziegler; individualCount: 1; lifeStage: Adult; behavior: On seafloor; occurrenceStatus: present; preparations: Imaged only; associatedReferences: Amon DJ, Ziegler AF, Dahlgren TG, Glover AG, Goineau A, Gooday AJ, Wiklund H, Smith CR. Insights into the abundance and diversity of abyssal megafauna in a polymetallic-nodule region in the eastern Clarion-Clipperton Zone. Scientific Reports. 2016;6. doi: 10.1038/srep30492; Leitner A, Neuheimer A, Donlon E, Smith CR, Drazen JC. Environmental and bathymetric influences on abyssal bait-attending communities of the Clarion Clipperton Zone. Deep Sea Research Part I: Oceanographic Research Papers. 2017. doi: 10.1016/j.dsr.2017.04.017; **Taxon:** taxonConceptID: Hymenopenaeus cf. nereus; scientificName: Hymenopenaeus nereus; kingdom: Animalia; phylum: Arthropoda; class: Malacostraca; order: Decapoda; family: Solenoceridae; genus: Hymenopeneaus; taxonRank: species; scientificNameAuthorship: Faxon, 1893; **Location:** waterBody: Pacific Ocean; stateProvince: Clarion-Clipperton Zone; locality: UK Seabed Resources Ltd exploration contract area (UK-1); verbatimLocality: UK-1 Stratum A; maximumDepthInMeters: 4070; locationRemarks: RV Melville Cruise MV1313; decimalLatitude: 13.7604; decimalLongitude: -116.4678; geodeticDatum: WGS84; coordinateUncertaintyInMeters: 25; **Identification:** identifiedBy: Mary Wicksten, Diva J. Amon, Amanda F. Ziegler; dateIdentified: 2014; identificationRemarks: Identified only from imagery; identificationQualifier: cf.; **Event:** samplingProtocol: Remotely Operated Vehicle; eventDate: 2013-10-18; eventTime: 2:44; habitat: Abyssal polymetallic-nodule field; fieldNumber: Dive 5 (RV05); **Record Level:** language: en; institutionCode: UHM; datasetName: ABYSSLINE; basisOfRecord: HumanObservation
- **Type status:** Other material. **Occurrence:** recordedBy: Jeffrey Drazen, Astrid Leitner; individualCount: 1; lifeStage: Adult; behavior: On seafloor; occurrenceStatus: present; preparations: Imaged only; associatedReferences: Amon DJ, Ziegler AF, Dahlgren TG, Glover AG, Goineau A, Gooday AJ, Wiklund H, Smith CR. Insights into the abundance and diversity of abyssal megafauna in a polymetallic-nodule region in the eastern Clarion-Clipperton Zone. Scientific Reports. 2016;6. doi: 10.1038/srep30492; Leitner A, Neuheimer A, Donlon E, Smith CR, Drazen JC. Environmental and bathymetric influences on abyssal bait-attending communities of the Clarion Clipperton Zone. Deep Sea Research Part I: Oceanographic Research Papers. 2017. doi: 10.1016/j.dsr.2017.04.017; **Taxon:** taxonConceptID: Hymenopenaeus cf. nereus; scientificName: Hymenopenaeus nereus; kingdom: Animalia; phylum: Arthropoda; class: Malacostraca; order: Decapoda; family: Solenoceridae; genus: Hymenopeneaus; taxonRank: species; scientificNameAuthorship: Faxon, 1893; **Location:** waterBody: Pacific Ocean; stateProvince: Clarion-Clipperton Zone; locality: UK Seabed Resources Ltd exploration contract area (UK-1); verbatimLocality: UK-1 Stratum B; maximumDepthInMeters: 4267; locationRemarks: RV Thompson Cruise TN319; decimalLatitude: 12.5667; decimalLongitude: -116.7082; geodeticDatum: WGS84; coordinateUncertaintyInMeters: 50; **Identification:** identifiedBy: Mary Wicksten, Jeffrey Drazen, Astrid Leitner, Diva J. Amon, Amanda Ziegler; dateIdentified: 2015; identificationRemarks: Identified only from imagery; identificationQualifier: cf.; **Event:** samplingProtocol: Baited Camera; eventDate: 2015-02-21; eventTime: 20:06; habitat: Abyssal polymetallic-nodule field; fieldNumber: CA02; **Record Level:** language: en; institutionCode: UHM; datasetName: ABYSSLINE; basisOfRecord: HumanObservation

##### Notes

Short rostrum with few teeth, slender abdomen, very long thread-like legs, second abdominal somite does not overlap first or third somite.

Fig. 18

#### Stylodactylidae

Spence Bate, 1888

#### Bathystylodactylus

Hanamura & Takeda, 1996

#### cf. Bathystylodactylus morphospecies

##### Materials

- **Type status:** Other material. **Occurrence:** recordedBy: Diva J. Amon, Amanda F. Ziegler; individualCount: 1; lifeStage: Adult; behavior: On seafloor; occurrenceStatus: present; preparations: Imaged only; **Taxon:** taxonConceptID: cf. *Bathystylodactylus* morphospecies; scientificName: *Bathystylodactylus* sp.; kingdom: Animalia; phylum: Arthropoda; class: Malacostraca; order: Decapoda; family: Stylodactylidae; genus: Bathystylodactylus; taxonRank: genus; scientificNameAuthorship: Hanamura & Takeda, 1996; **Location:** waterBody: Pacific Ocean; stateProvince: Clarion-Clipperton Zone; locality: Eastern Clarion-Clipperton Zone; verbatimLocality: Site EPIRB; maximumDepthInMeters: 3922; locationRemarks: RV Melville Cruise MV1313; decimalLatitude: 13.6786; decimalLongitude: -114.4074; geodeticDatum: WGS84; coordinateUncertaintyInMeters: 25; **Identification:** identifiedBy: Mary Wicksten, Diva J. Amon, Amanda F. Ziegler; dateIdentified: 2014; identificationRemarks: Identified only from imagery; identificationQualifier: cf.; **Event:** samplingProtocol: Remotely Operated Vehicle; eventDate: 2013-10-23; eventTime: 11:07; habitat: Abyssal polymetallic-nodule field; fieldNumber: Dive 7 (RV07); **Record Level:** language: en; institutionCode: UHM; datasetName: ABYSSLINE; basisOfRecord: HumanObservation

##### Notes

Elongate setose legs and rostrum, abdominal appendages do not protrude laterally, shrimp is pale pink, not red, posture is with front of body angled slightly upward from sediment surface.

Fig. 19

#### Isopoda

Latreille, 1817

#### Munnopsidae

Lilljeborg, 1864

#### cf. Munnopsidae morphospecies

##### Materials

- **Type status:** Other material. **Occurrence:** recordedBy: Diva J. Amon, Amanda F. Ziegler; individualCount: 1; lifeStage: Adult; behavior: On a sponge; occurrenceStatus: present; preparations: Imaged only; associatedReferences: Amon DJ, Ziegler AF, Dahlgren TG, Glover AG, Goineau A, Gooday AJ, Wiklund H, Smith CR. Insights into the abundance and diversity of abyssal megafauna in a polymetallic-nodule region in the eastern Clarion-Clipperton Zone. Scientific Reports. 2016;6. doi: 10.1038/srep30492; **Taxon:** taxonConceptID: cf. Munnopsidae morphospecies; scientificName: Munnopsidae sp.; kingdom: Animalia; phylum: Arthropoda; class: Malacostraca; order: Isopoda; family: Munnopsidae; taxonRank: family; scientificNameAuthorship: Lilljeborg, 1864; **Location:** waterBody: Pacific Ocean; stateProvince: Clarion-Clipperton Zone; locality: UK Seabed Resources Ltd exploration contract area (UK-1); verbatimLocality: UK-1 Stratum A; maximumDepthInMeters: 4070; locationRemarks: RV Melville Cruise MV1313; decimalLatitude: 13.7603; decimalLongitude: -116.4678; geodeticDatum: WGS84; coordinateUncertaintyInMeters: 25; **Identification:** identifiedBy: Mary Wicksten, Diva J. Amon, Amanda F. Ziegler; dateIdentified: 2014; identificationRemarks: Identified only from imagery; identificationQualifier: cf.; **Event:** samplingProtocol: Remotely Operated Vehicle; eventDate: 2013-10-18; eventTime: 2:57; habitat: Abyssal polymetallic-nodule field; fieldNumber: Dive 5 (RV05); **Record Level:** language: en; institutionCode: UHM; datasetName: ABYSSLINE; basisOfRecord: HumanObservation
- **Type status:** Other material. **Occurrence:** recordedBy: Diva J. Amon, Amanda F. Ziegler; individualCount: 1; lifeStage: Adult; behavior: On seafloor; occurrenceStatus: present; preparations: Imaged only; associatedReferences: Amon DJ, Ziegler AF, Dahlgren TG, Glover AG, Goineau A, Gooday AJ, Wiklund H, Smith CR. Insights into the abundance and diversity of abyssal megafauna in a polymetallic-nodule region in the eastern Clarion-Clipperton Zone. Scientific Reports. 2016;6. doi: 10.1038/srep30492; **Taxon:** taxonConceptID: cf. Munnopsidae morphospecies; scientificName: Munnopsidae sp.; kingdom: Animalia; phylum: Arthropoda; class: Malacostraca; order: Isopoda; family: Munnopsidae; taxonRank: family; scientificNameAuthorship: Lilljeborg, 1864; **Location:** waterBody: Pacific Ocean; stateProvince: Clarion-Clipperton Zone; locality: UK Seabed Resources Ltd exploration contract area (UK-1); verbatimLocality: UK-1 Stratum A; maximumDepthInMeters: 4110; locationRemarks: RV Melville Cruise MV1313; decimalLatitude: 13.8500; decimalLongitude: -116.6456; geodeticDatum: WGS84; coordinateUncertaintyInMeters: 25; **Identification:** identifiedBy: Mary Wicksten, Diva J. Amon, Amanda F. Ziegler; dateIdentified: 2014; identificationRemarks: Identified only from imagery; identificationQualifier: cf.; **Event:** samplingProtocol: Remotely Operated Vehicle; eventDate: 2013-10-10; eventTime: 11:27; habitat: Abyssal polymetallic-nodule field; fieldNumber: Dive 1 (RV01); **Record Level:** language: en; institutionCode: UHM; datasetName: ABYSSLINE; basisOfRecord: HumanObservation

##### Notes

Like amphipods, isopods lack a carapace and have eyes set into the head. Very long spider-like legs and compact body. Distinguished from true sea spiders (Pycnogonida), which have a long anterior proboscis and a segmented slender body.

Fig. 20

#### Hexanauplia

Oakley, Wolfe, Lindgren & Zaharof, 2013

#### Scalpelliformes

Buckeridge & Newman, 2006

#### Scalpellidae

Pilsbry, 1907

#### Neoscalpellum

Pilsbry, 1907

#### cf. Neoscalpellum morphospecies

##### Materials

- **Type status:** Other material. **Occurrence:** recordedBy: Diva J. Amon, Amanda F. Ziegler; individualCount: 1; lifeStage: Adult; behavior: On nodule; occurrenceStatus: present; preparations: Imaged only; associatedReferences: Amon DJ, Ziegler AF, Dahlgren TG, Glover AG, Goineau A, Gooday AJ, Wiklund H, Smith CR. Insights into the abundance and diversity of abyssal megafauna in a polymetallic-nodule region in the eastern Clarion-Clipperton Zone. Scientific Reports. 2016;6. doi: 10.1038/srep30492; **Taxon:** taxonConceptID: cf. *Neoscalpellum* morphospecies; scientificName: *Neoscalpellum* sp.; kingdom: Animalia; phylum: Arthropoda; class: Hexanauplia; order: Scalpelliformes; family: Scalpellidae; genus: Neoscalpellum; taxonRank: genus; scientificNameAuthorship: Pilsbry, 1907; **Location:** waterBody: Pacific Ocean; stateProvince: Clarion-Clipperton Zone; locality: UK Seabed Resources Ltd exploration contract area (UK-1); verbatimLocality: UK-1 Stratum A; maximumDepthInMeters: 4025; locationRemarks: RV Melville Cruise MV1313; decimalLatitude: 13.8646; decimalLongitude: -116.5481; geodeticDatum: WGS84; coordinateUncertaintyInMeters: 25; **Identification:** identifiedBy: Mary Wicksten, Diva J. Amon, Amanda F. Ziegler; dateIdentified: 2014; identificationRemarks: Identified only from imagery; identificationQualifier: cf.; **Event:** samplingProtocol: Remotely Operated Vehicle; eventDate: 2013-10-21; eventTime: 6:10; habitat: Abyssal polymetallic-nodule field; fieldNumber: Dive 6 (RV06); **Record Level:** language: en; institutionCode: UHM; datasetName: ABYSSLINE; basisOfRecord: HumanObservation

##### Notes

Ovate upper area (the capitulum) and large uncalcified areas between plates.

Fig. 21

### Bryozoans of the UKSRL exploration contract area (UK-1) and the eastern Clarion-Clipperton Zone

#### Bryozoa

Ehrenberg, 1831

#### Gymnolaemata

Allman, 1856

#### Cheilostomatida

Busk, 1852

#### Bifaxarioidea

Busk, 1884

#### Bifaxariidae

Busk, 1884

#### Smithsonius

Gordon, 1988

#### cf. Smithsonius morphospecies

##### Materials

- **Type status:** Other material. **Occurrence:** recordedBy: Diva J. Amon, Amanda F. Ziegler; individualCount: 1; lifeStage: Adult; behavior: On nodule; occurrenceStatus: present; preparations: Imaged only; **Taxon:** taxonConceptID: cf. *Smithsonius* morphospecies; scientificName: *Smithsonius* sp.; kingdom: Animalia; phylum: Bryozoa; class: Gymnolaemata; order: Cheilostomatida; family: Bifaxariidae; genus: Smithsonius; taxonRank: genus; scientificNameAuthorship: Gordon, 1988; **Location:** waterBody: Pacific Ocean; stateProvince: Clarion-Clipperton Zone; locality: UK Seabed Resources Ltd exploration contract area (UK-1); verbatimLocality: UK-1 Stratum A; maximumDepthInMeters: 4024; locationRemarks: RV Melville Cruise MV1313; decimalLatitude: 13.8646; decimalLongitude: -116.5481; geodeticDatum: WGS84; coordinateUncertaintyInMeters: 25; **Identification:** identifiedBy: Andrei Grischenko, Diva J. Amon, Amanda F. Ziegler; dateIdentified: 2014; identificationRemarks: Identified only from imagery; identificationQualifier: cf.; **Event:** samplingProtocol: Remotely Operated Vehicle; eventDate: 2013-10-21; eventTime: 8:24; habitat: Abyssal polymetallic-nodule field; fieldNumber: Dive 6 (RV06); **Record Level:** language: en; institutionCode: UHM; datasetName: ABYSSLINE; basisOfRecord: HumanObservation
- **Type status:** Other material. **Occurrence:** recordedBy: Diva J. Amon, Amanda F. Ziegler; individualCount: 1; lifeStage: Adult; behavior: On nodule; occurrenceStatus: present; preparations: Imaged only; **Taxon:** taxonConceptID: cf. *Smithsonius* morphospecies; scientificName: *Smithsonius* sp.; kingdom: Animalia; phylum: Bryozoa; class: Gymnolaemata; order: Cheilostomatida; family: Bifaxariidae; genus: Smithsonius; taxonRank: genus; scientificNameAuthorship: Gordon, 1988; **Location:** waterBody: Pacific Ocean; stateProvince: Clarion-Clipperton Zone; locality: UK Seabed Resources Ltd exploration contract area (UK-1); verbatimLocality: UK-1 Stratum A; maximumDepthInMeters: 4119; locationRemarks: RV Melville Cruise MV1313; decimalLatitude: 13.8498; decimalLongitude: -116.6457; geodeticDatum: WGS84; coordinateUncertaintyInMeters: 25; **Identification:** identifiedBy: Andrei Grischenko, Diva J. Amon, Amanda F. Ziegler; dateIdentified: 2014; identificationRemarks: Identified only from imagery; identificationQualifier: cf.; **Event:** samplingProtocol: Remotely Operated Vehicle; eventDate: 2013-10-10; eventTime: 12:39; habitat: Abyssal polymetallic-nodule field; fieldNumber: Dive 1 (RV01); **Record Level:** language: en; institutionCode: UHM; datasetName: ABYSSLINE; basisOfRecord: HumanObservation
- **Type status:** Other material. **Occurrence:** recordedBy: Diva J. Amon, Amanda F. Ziegler; individualCount: 1; lifeStage: Adult; behavior: On nodule; occurrenceStatus: present; preparations: Imaged only; **Taxon:** taxonConceptID: cf. *Smithsonius* morphospecies; scientificName: *Smithsonius* sp.; kingdom: Animalia; phylum: Bryozoa; class: Gymnolaemata; order: Cheilostomatida; family: Bifaxariidae; genus: Smithsonius; taxonRank: genus; scientificNameAuthorship: Gordon, 1988; **Location:** waterBody: Pacific Ocean; stateProvince: Clarion-Clipperton Zone; locality: Eastern Clarion-Clipperton Zone; verbatimLocality: Site EPIRB; maximumDepthInMeters: 3944; locationRemarks: RV Melville Cruise MV1313; decimalLatitude: 13.6794; decimalLongitude: -114.4126; geodeticDatum: WGS84; coordinateUncertaintyInMeters: 25; **Identification:** identifiedBy: Andrei Grischenko, Diva J. Amon, Amanda F. Ziegler; dateIdentified: 2014; identificationRemarks: Identified only from imagery; identificationQualifier: cf.; **Event:** samplingProtocol: Remotely Operated Vehicle; eventDate: 2013-10-23; eventTime: 10:10; habitat: Abyssal polymetallic-nodule field; fieldNumber: Dive 7 (RV07); **Record Level:** language: en; institutionCode: UHM; datasetName: ABYSSLINE; basisOfRecord: HumanObservation
- **Type status:** Other material. **Occurrence:** recordedBy: Diva J. Amon, Amanda F. Ziegler; individualCount: 1; lifeStage: Adult; behavior: On nodule; occurrenceStatus: present; preparations: Imaged only; **Taxon:** taxonConceptID: cf. *Smithsonius* morphospecies; scientificName: *Smithsonius* sp.; kingdom: Animalia; phylum: Bryozoa; class: Gymnolaemata; order: Cheilostomatida; family: Bifaxariidae; genus: Smithsonius; taxonRank: genus; scientificNameAuthorship: Gordon, 1988; **Location:** waterBody: Pacific Ocean; stateProvince: Clarion-Clipperton Zone; locality: Eastern Clarion-Clipperton Zone; verbatimLocality: Site EPIRB; maximumDepthInMeters: 3953; locationRemarks: RV Melville Cruise MV1313; decimalLatitude: 13.6797; decimalLongitude: -114.4146; geodeticDatum: WGS84; coordinateUncertaintyInMeters: 25; **Identification:** identifiedBy: Andrei Grischenko, Diva J. Amon, Amanda F. Ziegler; dateIdentified: 2014; identificationRemarks: Identified only from imagery; identificationQualifier: cf.; **Event:** samplingProtocol: Remotely Operated Vehicle; eventDate: 2013-10-23; eventTime: 13:21; habitat: Abyssal polymetallic-nodule field; fieldNumber: Dive 7 (RV07); **Record Level:** language: en; institutionCode: UHM; datasetName: ABYSSLINE; basisOfRecord: HumanObservation

##### Notes

Fixed-erect colonies with solid bifurcating branches possessing alternately arranged zooids facing in the opposite directions.

Fig. 22

#### Buguloidea

Gray, 1848

#### Candidae

d'Orbigny, 1851

#### Notoplites

Harmer, 1923

#### cf. Notoplites morphospecies

##### Materials

- **Type status:** Other material. **Occurrence:** recordedBy: Diva J. Amon, Amanda F. Ziegler; individualCount: 1; lifeStage: Adult; behavior: On nodule; occurrenceStatus: present; preparations: Imaged only; **Taxon:** taxonConceptID: cf. *Notoplites* morphospecies; scientificName: *Notoplites* sp.; kingdom: Animalia; phylum: Bryozoa; class: Gymnolaemata; order: Cheilostomatida; family: Candidae; genus: Notoplites; taxonRank: genus; scientificNameAuthorship: Harmer, 1923; **Location:** waterBody: Pacific Ocean; stateProvince: Clarion-Clipperton Zone; locality: UK Seabed Resources Ltd exploration contract area (UK-1); verbatimLocality: UK-1 Stratum A; maximumDepthInMeters: 4110; locationRemarks: RV Melville Cruise MV1313; decimalLatitude: 13.8484; decimalLongitude: -116.6567; geodeticDatum: WGS84; coordinateUncertaintyInMeters: 25; **Identification:** identifiedBy: Andrei Grischenko, Diva J. Amon, Amanda F. Ziegler; dateIdentified: 2014; identificationRemarks: Identified only from imagery; identificationQualifier: cf.; **Event:** samplingProtocol: Remotely Operated Vehicle; eventDate: 2013-10-10; eventTime: 11:10; habitat: Abyssal polymetallic-nodule field; fieldNumber: Dive 1 (RV01); **Record Level:** language: en; institutionCode: UHM; datasetName: ABYSSLINE; basisOfRecord: HumanObservation
- **Type status:** Other material. **Occurrence:** recordedBy: Diva J. Amon, Amanda F. Ziegler; individualCount: 1; lifeStage: Adult; behavior: On nodule; occurrenceStatus: present; preparations: Imaged only; **Taxon:** taxonConceptID: cf. *Notoplites* morphospecies; scientificName: *Notoplites* sp.; kingdom: Animalia; phylum: Bryozoa; class: Gymnolaemata; order: Cheilostomatida; family: Candidae; genus: Notoplites; taxonRank: genus; scientificNameAuthorship: Harmer, 1923; **Location:** waterBody: Pacific Ocean; stateProvince: Clarion-Clipperton Zone; locality: Eastern Clarion-Clipperton Zone; verbatimLocality: Site EPIRB; maximumDepthInMeters: 3914; locationRemarks: RV Melville Cruise MV1313; decimalLatitude: 13.6793; decimalLongitude: -114.4072; geodeticDatum: WGS84; coordinateUncertaintyInMeters: 25; **Identification:** identifiedBy: Andrei Grischenko, Diva J. Amon, Amanda F. Ziegler; dateIdentified: 2014; identificationRemarks: Identified only from imagery; identificationQualifier: cf.; **Event:** samplingProtocol: Remotely Operated Vehicle; eventDate: 2013-10-23; eventTime: 11:52; habitat: Abyssal polymetallic-nodule field; fieldNumber: Dive 7 (RV07); **Record Level:** language: en; institutionCode: UHM; datasetName: ABYSSLINE; basisOfRecord: HumanObservation
- **Type status:** Other material. **Occurrence:** recordedBy: Diva J. Amon, Amanda F. Ziegler; individualCount: 1; lifeStage: Adult; behavior: On nodule; occurrenceStatus: present; preparations: Imaged only; **Taxon:** taxonConceptID: cf. *Notoplites* morphospecies; scientificName: *Notoplites* sp.; kingdom: Animalia; phylum: Bryozoa; class: Gymnolaemata; order: Cheilostomatida; family: Candidae; genus: Notoplites; taxonRank: genus; scientificNameAuthorship: Harmer, 1923; **Location:** waterBody: Pacific Ocean; stateProvince: Clarion-Clipperton Zone; locality: UK Seabed Resources Ltd exploration contract area (UK-1); verbatimLocality: UK-1 Stratum A; maximumDepthInMeters: 4110; locationRemarks: RV Melville Cruise MV1313; decimalLatitude: 13.8502; decimalLongitude: -116.6457; geodeticDatum: WGS84; coordinateUncertaintyInMeters: 25; **Identification:** identifiedBy: Andrei Grischenko, Diva J. Amon, Amanda F. Ziegler; dateIdentified: 2014; identificationRemarks: Identified only from imagery; identificationQualifier: cf.; **Event:** samplingProtocol: Remotely Operated Vehicle; eventDate: 2013-10-10; eventTime: 12:05; habitat: Abyssal polymetallic-nodule field; fieldNumber: Dive 1 (RV01); **Record Level:** language: en; institutionCode: UHM; datasetName: ABYSSLINE; basisOfRecord: HumanObservation
- **Type status:** Other material. **Occurrence:** recordedBy: Diva J. Amon, Amanda F. Ziegler; individualCount: 1; lifeStage: Adult; behavior: On nodule; occurrenceStatus: present; preparations: Imaged only; **Taxon:** taxonConceptID: cf. *Notoplites* morphospecies; scientificName: *Notoplites* sp.; kingdom: Animalia; phylum: Bryozoa; class: Gymnolaemata; order: Cheilostomatida; family: Candidae; genus: Notoplites; taxonRank: genus; scientificNameAuthorship: Harmer, 1923; **Location:** waterBody: Pacific Ocean; stateProvince: Clarion-Clipperton Zone; locality: UK Seabed Resources Ltd exploration contract area (UK-1); verbatimLocality: UK-1 Stratum A; maximumDepthInMeters: 4026; locationRemarks: RV Melville Cruise MV1313; decimalLatitude: 13.8647; decimalLongitude: -116.5482; geodeticDatum: WGS84; coordinateUncertaintyInMeters: 25; **Identification:** identifiedBy: Andrei Grischenko, Diva J. Amon, Amanda F. Ziegler; dateIdentified: 2014; identificationRemarks: Identified only from imagery; identificationQualifier: cf.; **Event:** samplingProtocol: Remotely Operated Vehicle; eventDate: 2013-10-21; eventTime: 5:34; habitat: Abyssal polymetallic-nodule field; fieldNumber: Dive 6 (RV06); **Record Level:** language: en; institutionCode: UHM; datasetName: ABYSSLINE; basisOfRecord: HumanObservation

##### Notes

Rooted arborescent colonies with delicate bifurcating branches interconnected by transverse parallel series of chitinous tubes (fibers).

Fig. 23

#### Calloporoidea

Norman, 1903

#### Farciminariidae

Busk, 1852

#### Columnella

Levinsen, 1914

#### cf. Columnella morphospecies

##### Materials

- **Type status:** Other material. **Occurrence:** recordedBy: Diva J. Amon, Amanda F. Ziegler; individualCount: 1; lifeStage: Adult; behavior: On nodule; occurrenceStatus: present; preparations: Imaged only; **Taxon:** taxonConceptID: cf. *Columnella* morphospecies; scientificName: *Columnella* sp.; kingdom: Animalia; phylum: Bryozoa; class: Gymnolaemata; order: Cheilostomatida; family: Farciminariidae; genus: Columnella; taxonRank: genus; scientificNameAuthorship: Levinsen, 1914; **Location:** waterBody: Pacific Ocean; stateProvince: Clarion-Clipperton Zone; locality: UK Seabed Resources Ltd exploration contract area (UK-1); verbatimLocality: UK-1 Stratum A; maximumDepthInMeters: 4110; locationRemarks: RV Melville Cruise MV1313; decimalLatitude: 13.8500; decimalLongitude: -116.6456; geodeticDatum: WGS84; coordinateUncertaintyInMeters: 25; **Identification:** identifiedBy: Andrei Grischenko, Diva J. Amon, Amanda F. Ziegler; dateIdentified: 2014; identificationRemarks: Identified only from imagery; identificationQualifier: cf.; **Event:** samplingProtocol: Remotely Operated Vehicle; eventDate: 2013-10-10; eventTime: 13:17; habitat: Abyssal polymetallic-nodule field; fieldNumber: Dive 1 (RV01); **Record Level:** language: en; institutionCode: UHM; datasetName: ABYSSLINE; basisOfRecord: HumanObservation

##### Notes

Rooted arborescent, lightly calcified colony with slender, bifurcating branches.

Fig. 24

#### Lepralielloidea

Vigneaux, 1949

#### cf. Lepralielloidea morphospecies

##### Materials

- **Type status:** Other material. **Occurrence:** recordedBy: Diva J. Amon, Amanda F. Ziegler; individualCount: 1; lifeStage: Adult; behavior: On nodule; occurrenceStatus: present; preparations: Imaged only; **Taxon:** taxonConceptID: cf. Lepralielloidea morphospecies; scientificName: Lepralielloidea sp.; kingdom: Animalia; phylum: Bryozoa; class: Gymnolaemata; order: Cheilostomatida; taxonRank: superfamily; scientificNameAuthorship: Vigneaux, 1949; **Location:** waterBody: Pacific Ocean; stateProvince: Clarion-Clipperton Zone; locality: UK Seabed Resources Ltd exploration contract area (UK-1); verbatimLocality: UK-1 Stratum A; maximumDepthInMeters: 4110; locationRemarks: RV Melville Cruise MV1313; decimalLatitude: 13.8484; decimalLongitude: -116.6567; geodeticDatum: WGS84; coordinateUncertaintyInMeters: 25; **Identification:** identifiedBy: Andrei Grischenko, Diva J. Amon, Amanda F. Ziegler; dateIdentified: 2014; identificationRemarks: Identified only from imagery; identificationQualifier: cf.; **Event:** samplingProtocol: Remotely Operated Vehicle; eventDate: 2013-10-10; eventTime: 11:10; habitat: Abyssal polymetallic-nodule field; fieldNumber: Dive 1 (RV01); **Record Level:** language: en; institutionCode: UHM; datasetName: ABYSSLINE; basisOfRecord: HumanObservation

##### Notes

Fixed-erect colony with relatively short stem followed by four solid dichotomous branches.

Fig. 25

### Chordates of the UKSRL exploration contract area (UK-1) and the eastern Clarion-Clipperton Zone

#### Chordata

Haeckel, 1874

#### Ascidiacea

Blainville, 1824

#### Phlebobranchia

Lahille, 1886

#### cf. Phlebobranchia morphospecies 1

##### Materials

- **Type status:** Other material. **Occurrence:** recordedBy: Diva J. Amon, Amanda F. Ziegler; individualCount: 1; lifeStage: Adult; behavior: On nodule; occurrenceStatus: present; preparations: Imaged only; associatedReferences: Amon DJ, Ziegler AF, Dahlgren TG, Glover AG, Goineau A, Gooday AJ, Wiklund H, Smith CR. Insights into the abundance and diversity of abyssal megafauna in a polymetallic-nodule region in the eastern Clarion-Clipperton Zone. Scientific Reports. 2016;6. doi: 10.1038/srep30492; **Taxon:** taxonConceptID: cf. Phlebobranchia morphospecies 1; scientificName: Phlebobranchia sp.; kingdom: Animalia; phylum: Chordata; class: Ascidiacea; order: Phlebobranchia; taxonRank: order; scientificNameAuthorship: Lahille, 1886; **Location:** waterBody: Pacific Ocean; stateProvince: Clarion-Clipperton Zone; locality: UK Seabed Resources Ltd exploration contract area (UK-1); verbatimLocality: UK-1 Stratum A; maximumDepthInMeters: 4033; locationRemarks: RV Melville Cruise MV1313; decimalLatitude: 13.8636; decimalLongitude: -116.5486; geodeticDatum: WGS84; coordinateUncertaintyInMeters: 25; **Identification:** identifiedBy: Craig Young, Diva J. Amon, Amanda F. Ziegler; dateIdentified: 2014; identificationRemarks: Identified only from imagery; identificationQualifier: cf.; **Event:** samplingProtocol: Remotely Operated Vehicle; eventDate: 2013-10-21; eventTime: 5:10; habitat: Abyssal polymetallic-nodule field; fieldNumber: Dive 6 (RV06); **Record Level:** language: en; institutionCode: UHM; datasetName: ABYSSLINE; basisOfRecord: HumanObservation

##### Notes

Elongate phlebobranch ascidian. A branching transparent stolon to the left of the ascidian may be part of the individual, but this is not certain.

Fig. 26

#### cf. Phlebobranchia morphospecies 2

##### Materials

- **Type status:** Other material. **Occurrence:** recordedBy: Diva J. Amon, Amanda F. Ziegler; individualCount: 1; lifeStage: Adult; behavior: On nodule; occurrenceStatus: present; preparations: Imaged only; **Taxon:** taxonConceptID: cf. Phlebobranchia morphospecies 2; scientificName: Phlebobranchia sp.; kingdom: Animalia; phylum: Chordata; class: Ascidiacea; order: Phlebobranchia; taxonRank: order; scientificNameAuthorship: Lahille, 1886; **Location:** waterBody: Pacific Ocean; stateProvince: Clarion-Clipperton Zone; locality: UK Seabed Resources Ltd exploration contract area (UK-1); verbatimLocality: UK-1 Stratum A; maximumDepthInMeters: 4101; locationRemarks: RV Melville Cruise MV1313; decimalLatitude: 13.8518; decimalLongitude: -116.6448; geodeticDatum: WGS84; coordinateUncertaintyInMeters: 25; **Identification:** identifiedBy: Craig Young, Diva J. Amon, Amanda F. Ziegler; dateIdentified: 2014; identificationRemarks: Identified only from imagery; identificationQualifier: cf.; **Event:** samplingProtocol: Remotely Operated Vehicle; eventDate: 2013-10-10; eventTime: 16:57; habitat: Abyssal polymetallic-nodule field; fieldNumber: Dive 1 (RV01); **Record Level:** language: en; institutionCode: UHM; datasetName: ABYSSLINE; basisOfRecord: HumanObservation

##### Notes

Very transparent ascidian approximately twice as long as it is wide. Larger incurrent siphon points anteriorally. Smaller atrial siphon points to the side. Rows of bright white spots are probably not part of the ascidian.

Fig. 27

#### Octacnemidae

Herdman, 1888

#### Dicopia

Sluiter, 1905

#### cf. Dicopia morphospecies

##### Materials

- **Type status:** Other material. **Occurrence:** recordedBy: Diva J. Amon, Amanda F. Ziegler; individualCount: 1; lifeStage: Adult; behavior: On nodule; occurrenceStatus: present; preparations: Imaged only; **Taxon:** taxonConceptID: cf. *Dicopia* morphospecies; scientificName: *Dicopia* sp.; kingdom: Animalia; phylum: Chordata; class: Ascidiacea; order: Phlebobranchia; family: Octacnemidae; genus: Dicopia; taxonRank: genus; scientificNameAuthorship: Sluiter, 1905; **Location:** waterBody: Pacific Ocean; stateProvince: Clarion-Clipperton Zone; locality: Eastern Clarion-Clipperton Zone; verbatimLocality: Site EPIRB; maximumDepthInMeters: 3927; locationRemarks: RV Melville Cruise MV1313; decimalLatitude: 13.6789; decimalLongitude: -114.4093; geodeticDatum: WGS84; coordinateUncertaintyInMeters: 25; **Identification:** identifiedBy: Craig Young, Diva J. Amon, Amanda F. Ziegler; dateIdentified: 2014; identificationRemarks: Identified only from imagery; identificationQualifier: cf.; **Event:** samplingProtocol: Remotely Operated Vehicle; eventDate: 2013-10-23; eventTime: 10:47; habitat: Abyssal polymetallic-nodule field; fieldNumber: Dive 7 (RV07); **Record Level:** language: en; institutionCode: UHM; datasetName: ABYSSLINE; basisOfRecord: HumanObservation
- **Type status:** Other material. **Occurrence:** recordedBy: Diva J. Amon, Amanda F. Ziegler; individualCount: 1; lifeStage: Adult; behavior: On nodule; occurrenceStatus: present; preparations: Imaged only; **Taxon:** taxonConceptID: cf. *Dicopia* morphospecies; scientificName: *Dicopia* sp.; kingdom: Animalia; phylum: Chordata; class: Ascidiacea; order: Phlebobranchia; family: Octacnemidae; genus: Dicopia; taxonRank: genus; scientificNameAuthorship: Sluiter, 1905; **Location:** waterBody: Pacific Ocean; stateProvince: Clarion-Clipperton Zone; locality: UK Seabed Resources Ltd exploration contract area (UK-1); verbatimLocality: UK-1 Stratum A; maximumDepthInMeters: 4063; locationRemarks: RV Melville Cruise MV1313; decimalLatitude: 13.9666; decimalLongitude: -116.5573; geodeticDatum: WGS84; coordinateUncertaintyInMeters: 25; **Identification:** identifiedBy: Craig Young, Diva J. Amon, Amanda F. Ziegler; dateIdentified: 2014; identificationRemarks: Identified only from imagery; identificationQualifier: cf.; **Event:** samplingProtocol: Remotely Operated Vehicle; eventDate: 2013-10-15; eventTime: 23:58; habitat: Abyssal polymetallic-nodule field; fieldNumber: Dive 3 (RV03); **Record Level:** language: en; institutionCode: UHM; datasetName: ABYSSLINE; basisOfRecord: HumanObservation

##### Notes

Attached to polymetallic nodules by a short, thick stalk of transparent tunic. Expansive incurrent siphon appears as a smiling mouth on the side of the animal. Large incurrent (atrial) siphon on top appears as a transparent region when open. Nearly circular in shape when viewed from above.

Fig. 28

#### Megalodicopia

Oka, 1918

#### cf. Megalodicopia morphospecies

##### Materials

- **Type status:** Other material. **Occurrence:** recordedBy: Diva J. Amon, Amanda F. Ziegler; individualCount: 1; lifeStage: Adult; behavior: On nodule; occurrenceStatus: present; preparations: Imaged only; **Taxon:** taxonConceptID: cf. *Megalodicopia* morphospecies; scientificName: *Megalodicopia* sp.; kingdom: Animalia; phylum: Chordata; class: Ascidiacea; order: Phlebobranchia; family: Octacnemidae; genus: Megalodicopia; taxonRank: genus; scientificNameAuthorship: Oka, 1918; **Location:** waterBody: Pacific Ocean; stateProvince: Clarion-Clipperton Zone; locality: Eastern Clarion-Clipperton Zone; verbatimLocality: Site EPIRB; maximumDepthInMeters: 3920; locationRemarks: RV Melville Cruise MV1313; decimalLatitude: 13.6787; decimalLongitude: -114.4072; geodeticDatum: WGS84; coordinateUncertaintyInMeters: 25; **Identification:** identifiedBy: Craig Young, Diva J. Amon, Amanda F. Ziegler; dateIdentified: 2014; identificationRemarks: Identified only from imagery; identificationQualifier: cf.; **Event:** samplingProtocol: Remotely Operated Vehicle; eventDate: 2013-10-23; eventTime: 11:09; habitat: Abyssal polymetallic-nodule field; fieldNumber: Dive 7 (RV07); **Record Level:** language: en; institutionCode: UHM; datasetName: ABYSSLINE; basisOfRecord: HumanObservation
- **Type status:** Other material. **Occurrence:** recordedBy: Diva J. Amon, Amanda F. Ziegler; individualCount: 1; lifeStage: Adult; behavior: On nodule; occurrenceStatus: present; preparations: Imaged only; **Taxon:** taxonConceptID: cf. *Megalodicopia* morphospecies; scientificName: *Megalodicopia* sp.; kingdom: Animalia; phylum: Chordata; class: Ascidiacea; order: Phlebobranchia; family: Octacnemidae; genus: Megalodicopia; taxonRank: genus; scientificNameAuthorship: Oka, 1918; **Location:** waterBody: Pacific Ocean; stateProvince: Clarion-Clipperton Zone; locality: UK Seabed Resources Ltd exploration contract area (UK-1); verbatimLocality: UK-1 Stratum B; maximumDepthInMeters: 4242; locationRemarks: RV Melville Cruise MV1313; decimalLatitude: 12.4970; decimalLongitude: -116.6372; geodeticDatum: WGS84; coordinateUncertaintyInMeters: 25; **Identification:** identifiedBy: Craig Young, Diva J. Amon, Amanda F. Ziegler; dateIdentified: 2015; identificationRemarks: Identified only from imagery; identificationQualifier: cf.; **Event:** samplingProtocol: Autonomous Underwater Vehicle; eventDate: 2015-03-18; eventTime: 9:40; habitat: Abyssal polymetallic-nodule field; fieldNumber: Dive 9 (AV09); **Record Level:** language: en; institutionCode: UHM; datasetName: ABYSSLINE; basisOfRecord: HumanObservation

##### Notes

Round transparent ascidian on a thick stalk of tunic that stands higher off the bottom than *Dicopia*. Excurrent siphon is also much smaller than that of *Dicopia*.

Fig. 29

#### Situla

Vinogradova, 1969

#### cf. Situla morphospecies

##### Materials

- **Type status:** Other material. **Occurrence:** recordedBy: Diva J. Amon, Amanda F. Ziegler; individualCount: 1; lifeStage: Adult; behavior: On nodule; occurrenceStatus: present; preparations: Imaged only; **Taxon:** taxonConceptID: cf. *Situla* morphospecies; scientificName: *Situla* sp.; kingdom: Animalia; phylum: Chordata; class: Ascidiacea; order: Phlebobranchia; family: Octacnemidae; genus: Situla; taxonRank: genus; scientificNameAuthorship: Vinogradova, 1969; **Location:** waterBody: Pacific Ocean; stateProvince: Clarion-Clipperton Zone; locality: UK Seabed Resources Ltd exploration contract area (UK-1); verbatimLocality: UK-1 Stratum A; maximumDepthInMeters: 4057; locationRemarks: RV Melville Cruise MV1313; decimalLatitude: 13.9671; decimalLongitude: -116.5587; geodeticDatum: WGS84; coordinateUncertaintyInMeters: 25; **Identification:** identifiedBy: Craig Young, Diva J. Amon, Amanda F. Ziegler; dateIdentified: 2014; identificationRemarks: Identified only from imagery; identificationQualifier: cf.; **Event:** samplingProtocol: Remotely Operated Vehicle; eventDate: 2013-10-16; eventTime: 0:14; habitat: Abyssal polymetallic-nodule field; fieldNumber: Dive 3 (RV03); **Record Level:** language: en; institutionCode: UHM; datasetName: ABYSSLINE; basisOfRecord: HumanObservation
- **Type status:** Other material. **Occurrence:** recordedBy: Diva J. Amon, Amanda F. Ziegler; individualCount: 1; lifeStage: Adult; behavior: On nodule; occurrenceStatus: present; preparations: Imaged only; **Taxon:** taxonConceptID: cf. *Situla* morphospecies; scientificName: *Situla* sp.; kingdom: Animalia; phylum: Chordata; class: Ascidiacea; order: Phlebobranchia; family: Octacnemidae; genus: Situla; taxonRank: genus; scientificNameAuthorship: Vinogradova, 1969; **Location:** waterBody: Pacific Ocean; stateProvince: Clarion-Clipperton Zone; locality: UK Seabed Resources Ltd exploration contract area (UK-1); verbatimLocality: UK-1 Stratum B; maximumDepthInMeters: 4215; locationRemarks: RV Melville Cruise MV1313; decimalLatitude: 12.5896; decimalLongitude: -116.7089; geodeticDatum: WGS84; coordinateUncertaintyInMeters: 25; **Identification:** identifiedBy: Craig Young, Diva J. Amon, Amanda F. Ziegler; dateIdentified: 2015; identificationRemarks: Identified only from imagery; identificationQualifier: cf.; **Event:** samplingProtocol: Autonomous Underwater Vehicle; eventDate: 2015-03-09; eventTime: 14:31; habitat: Abyssal polymetallic-nodule field; fieldNumber: Dive 6 (AV06); **Record Level:** language: en; institutionCode: UHM; datasetName: ABYSSLINE; basisOfRecord: HumanObservation
- **Type status:** Other material. **Occurrence:** recordedBy: Diva J. Amon, Amanda F. Ziegler; individualCount: 1; lifeStage: Adult; behavior: On nodule; occurrenceStatus: present; preparations: Imaged only; **Taxon:** taxonConceptID: cf. *Situla* morphospecies; scientificName: *Situla* sp.; kingdom: Animalia; phylum: Chordata; class: Ascidiacea; order: Phlebobranchia; family: Octacnemidae; genus: Situla; taxonRank: genus; scientificNameAuthorship: Vinogradova, 1969; **Location:** waterBody: Pacific Ocean; stateProvince: Clarion-Clipperton Zone; locality: UK Seabed Resources Ltd exploration contract area (UK-1); verbatimLocality: UK-1 Stratum A; maximumDepthInMeters: 4097; locationRemarks: RV Melville Cruise MV1313; decimalLatitude: 13.8527; decimalLongitude: -116.6444; geodeticDatum: WGS84; coordinateUncertaintyInMeters: 25; **Identification:** identifiedBy: Craig Young, Diva J. Amon, Amanda F. Ziegler; dateIdentified: 2014; identificationRemarks: Identified only from imagery; identificationQualifier: cf.; **Event:** samplingProtocol: Remotely Operated Vehicle; eventDate: 2013-10-10; eventTime: 17:05; habitat: Abyssal polymetallic-nodule field; fieldNumber: Dive 1 (RV01); **Record Level:** language: en; institutionCode: UHM; datasetName: ABYSSLINE; basisOfRecord: HumanObservation

##### Notes

Large transparent ascidian most often attached to the side of a rock by means of a short but stout tunic stalk. In contrast to *Dicopia* or *Megadicopia*, this species is somewhat elongated laterally. The excurrent siphon is smaller in diameter than that of *Dicopia*.

Fig. 30

#### Actinopterygii

#### cf. Actinopterygii morphospecies

##### Materials

- **Type status:** Other material. **Occurrence:** recordedBy: Diva J. Amon, Amanda F. Ziegler; individualCount: 1; lifeStage: Adult; behavior: Swimming; occurrenceStatus: present; preparations: Imaged only; **Taxon:** taxonConceptID: cf. Actinopterygii morphospecies; scientificName: Actinopterygii sp.; kingdom: Animalia; phylum: Chordata; class: Actinopterygii; taxonRank: class; **Location:** waterBody: Pacific Ocean; stateProvince: Clarion-Clipperton Zone; locality: UK Seabed Resources Ltd exploration contract area (UK-1); verbatimLocality: UK-1 Stratum B; maximumDepthInMeters: 4223; locationRemarks: RV Thompson Cruise TN319; decimalLatitude: 12.5887; decimalLongitude: -116.7169; geodeticDatum: WGS84; coordinateUncertaintyInMeters: 25; **Identification:** identifiedBy: Astrid Leitner, Jeffrey Drazen, Diva J. Amon, Amanda F. Ziegler; dateIdentified: 2015; identificationRemarks: Identified only from imagery; identificationQualifier: cf.; **Event:** samplingProtocol: Autonomous Underwater Vehicle; eventDate: 2015-03-09; eventTime: 8:30; habitat: Abyssal polymetallic-nodule field; fieldNumber: Dive 6 (AV06); **Record Level:** language: en; institutionCode: UHM; datasetName: ABYSSLINE; basisOfRecord: HumanObservation

##### Notes

Rounded head with large rounded pectoral fins tapering to a thin tail.

Fig. 31

#### Anguilliformes

#### Synaphobranchidae

Johnson, 1862

#### Histiobranchus

Gill, 1883

#### Histiobranchus cf. bathybius

Günther, 1877

Histiobranchus cf. bathybius In the “Atlas of Abyssal Megafauna Morphotypes of the Clarion-Clipperton Fracture Zone” created for the ISA (http://ccfzatlas.com/), this morphospecies is listed as "Synaphobranchidae morphotype".

##### Materials

- **Type status:** Other material. **Occurrence:** recordedBy: Diva J. Amon, Amanda F. Ziegler; individualCount: 1; lifeStage: Adult; behavior: Swimming; occurrenceStatus: present; preparations: Imaged only; associatedReferences: Leitner A, Neuheimer A, Donlon E, Smith CR, Drazen JC. Environmental and bathymetric influences on abyssal bait-attending communities of the Clarion Clipperton Zone. Deep Sea Research Part I: Oceanographic Research Papers. 2017. doi: 10.1016/j.dsr.2017.04.017; **Taxon:** taxonConceptID: Histiobranchus cf. bathybius; scientificName: Histiobranchus bathybius; kingdom: Animalia; phylum: Chordata; class: Actinopterygii; order: Anguilliformes; family: Synaphobranchidae; genus: Histiobranchus; taxonRank: species; scientificNameAuthorship: Günther, 1877; **Location:** waterBody: Pacific Ocean; stateProvince: Clarion-Clipperton Zone; locality: UK Seabed Resources Ltd exploration contract area (UK-1); verbatimLocality: UK-1 Stratum B; maximumDepthInMeters: 4249; locationRemarks: RV Thompson Cruise TN319; decimalLatitude: 12.5035; decimalLongitude: -116.6441; geodeticDatum: WGS84; coordinateUncertaintyInMeters: 25; **Identification:** identifiedBy: Astrid Leitner, Jeffrey Drazen, Diva J. Amon, Amanda F. Ziegler; dateIdentified: 2015; identificationRemarks: Identified only from imagery; identificationQualifier: cf.; **Event:** samplingProtocol: Autonomous Underwater Vehicle; eventDate: 2015-03-04; eventTime: 7:58; habitat: Abyssal polymetallic-nodule field; fieldNumber: Dive 5 (AV05); **Record Level:** language: en; institutionCode: UHM; datasetName: ABYSSLINE; basisOfRecord: HumanObservation
- **Type status:** Other material. **Occurrence:** recordedBy: Jeffrey Drazen, Astrid Leitner; individualCount: 1; lifeStage: Adult; behavior: Feeding on bait; occurrenceStatus: present; preparations: Imaged only; associatedReferences: Leitner A, Neuheimer A, Donlon E, Smith CR, Drazen JC. Environmental and bathymetric influences on abyssal bait-attending communities of the Clarion Clipperton Zone. Deep Sea Research Part I: Oceanographic Research Papers. 2017. doi: 10.1016/j.dsr.2017.04.017; **Taxon:** taxonConceptID: Histiobranchus cf. bathybius; scientificName: Histiobranchus bathybius; kingdom: Animalia; phylum: Chordata; class: Actinopterygii; order: Anguilliformes; family: Synaphobranchidae; genus: Histiobranchus; taxonRank: species; scientificNameAuthorship: Günther, 1877; **Location:** waterBody: Pacific Ocean; stateProvince: Clarion-Clipperton Zone; locality: UK Seabed Resources Ltd exploration contract area (UK-1); verbatimLocality: UK-1 Stratum B; maximumDepthInMeters: 4263; locationRemarks: RV Thompson Cruise TN319; decimalLatitude: 12.4526; decimalLongitude: -116.6488; geodeticDatum: WGS84; coordinateUncertaintyInMeters: 50; **Identification:** identifiedBy: Astrid Leitner, Jeffrey Drazen, Diva J. Amon, Amanda F. Ziegler; dateIdentified: 2015; identificationRemarks: Identified only from imagery; identificationQualifier: cf.; **Event:** samplingProtocol: Baited Camera; eventDate: 2015-03-07; eventTime: 17:06; habitat: Abyssal polymetallic-nodule field; fieldNumber: CA08; **Record Level:** language: en; institutionCode: UHM; datasetName: ABYSSLINE; basisOfRecord: HumanObservation

##### Notes

Elongated eel-like body with small pectoral fins and large jaws extending well past eye. Dorsal fin insertion before anus. Generally white to grey. No prominent lateral line pores. Notch near the end of the anal fin.

Fig. 32

#### Aulopiformes

#### Bathysauridae

Fowler, 1944

#### Bathysaurus

Günther, 1878

#### Bathysaurus cf. mollis

Günther, 1878

Bathysaurus cf. mollis In the “Atlas of Abyssal Megafauna Morphotypes of the Clarion-Clipperton Fracture Zone” created for the ISA (http://ccfzatlas.com/), this morphospecies is listed as "*Bathysaurus mollis*".

##### Materials

- **Type status:** Other material. **Occurrence:** recordedBy: Diva J. Amon, Amanda F. Ziegler; individualCount: 1; lifeStage: Adult; behavior: Swimming; occurrenceStatus: present; preparations: Imaged only; associatedReferences: Amon DJ, Ziegler AF, Dahlgren TG, Glover AG, Goineau A, Gooday AJ, Wiklund H, Smith CR. Insights into the abundance and diversity of abyssal megafauna in a polymetallic-nodule region in the eastern Clarion-Clipperton Zone. Scientific Reports. 2016;6. doi: 10.1038/srep30492; **Taxon:** taxonConceptID: Bathysaurus cf. mollis; scientificName: Bathysaurus mollis; kingdom: Animalia; phylum: Chordata; class: Actinopterygii; order: Aulopiformes; family: Bathysauridae; genus: Bathysaurus; taxonRank: species; scientificNameAuthorship: Gunther, 1878; **Location:** waterBody: Pacific Ocean; stateProvince: Clarion-Clipperton Zone; locality: UK Seabed Resources Ltd exploration contract area (UK-1); verbatimLocality: UK-1 Stratum A; maximumDepthInMeters: 4028; locationRemarks: RV Melville Cruise MV1313; decimalLatitude: 13.8618; decimalLongitude: -116.5484; geodeticDatum: WGS84; coordinateUncertaintyInMeters: 25; **Identification:** identifiedBy: Astrid Leitner, Jeffrey Drazen, Diva J. Amon, Amanda F. Ziegler; dateIdentified: 2014; identificationRemarks: Identified only from imagery; identificationQualifier: cf.; **Event:** samplingProtocol: Remotely Operated Vehicle; eventDate: 2013-10-21; eventTime: 4:45; habitat: Abyssal polymetallic-nodule field; fieldNumber: Dive 6 (RV06); **Record Level:** language: en; institutionCode: UHM; datasetName: ABYSSLINE; basisOfRecord: HumanObservation
- **Type status:** Other material. **Occurrence:** recordedBy: Diva J. Amon, Amanda F. Ziegler; individualCount: 1; lifeStage: Adult; behavior: Swimming; occurrenceStatus: present; preparations: Imaged only; associatedReferences: Amon DJ, Ziegler AF, Dahlgren TG, Glover AG, Goineau A, Gooday AJ, Wiklund H, Smith CR. Insights into the abundance and diversity of abyssal megafauna in a polymetallic-nodule region in the eastern Clarion-Clipperton Zone. Scientific Reports. 2016;6. doi: 10.1038/srep30492; **Taxon:** taxonConceptID: Bathysaurus cf. mollis; scientificName: Bathysaurus mollis; kingdom: Animalia; phylum: Chordata; class: Actinopterygii; order: Aulopiformes; family: Bathysauridae; genus: Bathysaurus; taxonRank: species; scientificNameAuthorship: Gunther, 1878; **Location:** waterBody: Pacific Ocean; stateProvince: Clarion-Clipperton Zone; locality: UK Seabed Resources Ltd exploration contract area (UK-1); verbatimLocality: UK-1 Stratum B; maximumDepthInMeters: 4226; locationRemarks: RV Thompson Cruise TN319; decimalLatitude: 12.5787; decimalLongitude: -116.7246; geodeticDatum: WGS84; coordinateUncertaintyInMeters: 25; **Identification:** identifiedBy: Astrid Leitner, Jeffrey Drazen, Diva J. Amon, Amanda F. Ziegler; dateIdentified: 2015; identificationRemarks: Identified only from imagery; identificationQualifier: cf.; **Event:** samplingProtocol: Autonomous Underwater Vehicle; eventDate: 2015-03-09; eventTime: 6:44; habitat: Abyssal polymetallic-nodule field; fieldNumber: Dive 6 (AV06); **Record Level:** language: en; institutionCode: UHM; datasetName: ABYSSLINE; basisOfRecord: HumanObservation

##### Notes

White to grey/silvery body with flattened, depressed head and gold to green, round reflective eyes. Large mouth with jaw extending past eye and prominent teeth. Typical posture is perched on substrate with all fins extended.

Fig. 33

#### Ipnopidae

Gill, 1884

#### Ipnops

Günther, 1878

#### Ipnops cf. meadi

Nielsen, 1966

Ipnops cf. meadi In the “Atlas of Abyssal Megafauna Morphotypes of the Clarion-Clipperton Fracture Zone” created for the ISA (http://ccfzatlas.com/), this morphospecies is listed as "*Ipnops* morphotype".

##### Materials

- **Type status:** Other material. **Occurrence:** recordedBy: Diva J. Amon, Amanda F. Ziegler; individualCount: 1; lifeStage: Adult; behavior: On seafloor; occurrenceStatus: present; preparations: Imaged only; associatedReferences: Amon DJ, Ziegler AF, Dahlgren TG, Glover AG, Goineau A, Gooday AJ, Wiklund H, Smith CR. Insights into the abundance and diversity of abyssal megafauna in a polymetallic-nodule region in the eastern Clarion-Clipperton Zone. Scientific Reports. 2016;6. doi: 10.1038/srep30492; **Taxon:** taxonConceptID: Ipnops cf. meadi; scientificName: Ipnops meadi; kingdom: Animalia; phylum: Chordata; class: Actinopterygii; order: Aulopiformes; family: Ipnopidae; genus: Ipnops; taxonRank: species; scientificNameAuthorship: Nielsen, 1966; **Location:** waterBody: Pacific Ocean; stateProvince: Clarion-Clipperton Zone; locality: UK Seabed Resources Ltd exploration contract area (UK-1); verbatimLocality: UK-1 Stratum A; maximumDepthInMeters: 4028; locationRemarks: RV Melville Cruise MV1313; decimalLatitude: 13.8637; decimalLongitude: -116.5462; geodeticDatum: WGS84; coordinateUncertaintyInMeters: 25; **Identification:** identifiedBy: Astrid Leitner, Jeffrey Drazen, Diva J. Amon, Amanda F. Ziegler; dateIdentified: 2014; identificationRemarks: Identified only from imagery; identificationQualifier: cf.; **Event:** samplingProtocol: Remotely Operated Vehicle; eventDate: 2013-10-21; eventTime: 1:09; habitat: Abyssal polymetallic-nodule field; fieldNumber: Dive 6 (RV06); **Record Level:** language: en; institutionCode: UHM; datasetName: ABYSSLINE; basisOfRecord: HumanObservation
- **Type status:** Other material. **Occurrence:** recordedBy: Diva J. Amon, Amanda F. Ziegler; individualCount: 1; lifeStage: Adult; behavior: On seafloor; occurrenceStatus: present; preparations: Imaged only; associatedReferences: Amon DJ, Ziegler AF, Dahlgren TG, Glover AG, Goineau A, Gooday AJ, Wiklund H, Smith CR. Insights into the abundance and diversity of abyssal megafauna in a polymetallic-nodule region in the eastern Clarion-Clipperton Zone. Scientific Reports. 2016;6. doi: 10.1038/srep30492; **Taxon:** taxonConceptID: Ipnops cf. meadi; scientificName: Ipnops meadi; kingdom: Animalia; phylum: Chordata; class: Actinopterygii; order: Aulopiformes; family: Ipnopidae; genus: Ipnops; taxonRank: species; scientificNameAuthorship: Nielsen, 1966; **Location:** waterBody: Pacific Ocean; stateProvince: Clarion-Clipperton Zone; locality: UK Seabed Resources Ltd exploration contract area (UK-1); verbatimLocality: UK-1 Stratum A; maximumDepthInMeters: 4027; locationRemarks: RV Melville Cruise MV1313; decimalLatitude: 13.8605; decimalLongitude: -116.5483; geodeticDatum: WGS84; coordinateUncertaintyInMeters: 25; **Identification:** identifiedBy: Astrid Leitner, Jeffrey Drazen, Diva J. Amon, Amanda F. Ziegler; dateIdentified: 2014; identificationRemarks: Identified only from imagery; identificationQualifier: cf.; **Event:** samplingProtocol: Remotely Operated Vehicle; eventDate: 2013-10-21; eventTime: 4:29; habitat: Abyssal polymetallic-nodule field; fieldNumber: Dive 6 (RV06); **Record Level:** language: en; institutionCode: UHM; datasetName: ABYSSLINE; basisOfRecord: HumanObservation

##### Notes

Thin slender, black body with large, bright, reflective, plate-like eyes and large mouth.

Fig. 34

#### Gadiformes

#### Macrouridae

Bonaparte, 1831

#### Coryphaenoides

Gunnerus, 1765

#### cf. Coryphaenoides morphospecies

cf. Coryphaenoides morphospecies In the “Atlas of Abyssal Megafauna Morphotypes of the Clarion-Clipperton Fracture Zone” created for the ISA (http://ccfzatlas.com/), this morphospecies is listed as "*Coryphaenoides* morphotype".

##### Materials

- **Type status:** Other material. **Occurrence:** recordedBy: Diva J. Amon, Amanda F. Ziegler; individualCount: 1; lifeStage: Adult; behavior: Swimming; occurrenceStatus: present; preparations: Imaged only; associatedReferences: Amon DJ, Ziegler AF, Dahlgren TG, Glover AG, Goineau A, Gooday AJ, Wiklund H, Smith CR. Insights into the abundance and diversity of abyssal megafauna in a polymetallic-nodule region in the eastern Clarion-Clipperton Zone. Scientific Reports. 2016;6. doi: 10.1038/srep30492; Leitner A, Neuheimer A, Donlon E, Smith CR, Drazen JC. Environmental and bathymetric influences on abyssal bait-attending communities of the Clarion Clipperton Zone. Deep Sea Research Part I: Oceanographic Research Papers. 2017. doi: 10.1016/j.dsr.2017.04.017; **Taxon:** taxonConceptID: cf. *Coryphaenoides* morphospecies; scientificName: *Coryphaenoides* sp.; kingdom: Animalia; phylum: Chordata; class: Actinopterygii; order: Gadiformes; family: Macrouridae; genus: Coryphaenoides; taxonRank: genus; scientificNameAuthorship: Gunnerus, 1765; **Location:** waterBody: Pacific Ocean; stateProvince: Clarion-Clipperton Zone; locality: UK Seabed Resources Ltd exploration contract area (UK-1); verbatimLocality: UK-1 Stratum A; maximumDepthInMeters: 4064; locationRemarks: RV Melville Cruise MV1313; decimalLatitude: 13.9628; decimalLongitude: -116.5510; geodeticDatum: WGS84; coordinateUncertaintyInMeters: 25; **Identification:** identifiedBy: Astrid Leitner, Jeffrey Drazen, Diva J. Amon, Amanda F. Ziegler; dateIdentified: 2014; identificationRemarks: Identified only from imagery; identificationQualifier: cf.; **Event:** samplingProtocol: Remotely Operated Vehicle; eventDate: 2013-10-16; eventTime: 5:15; habitat: Abyssal polymetallic-nodule field; fieldNumber: Dive 3 (RV03); **Record Level:** language: en; institutionCode: UHM; datasetName: ABYSSLINE; basisOfRecord: HumanObservation
- **Type status:** Other material. **Occurrence:** recordedBy: Diva J. Amon, Amanda F. Ziegler; individualCount: 1; lifeStage: Adult; behavior: Swimming; occurrenceStatus: present; preparations: Imaged only; associatedReferences: Amon DJ, Ziegler AF, Dahlgren TG, Glover AG, Goineau A, Gooday AJ, Wiklund H, Smith CR. Insights into the abundance and diversity of abyssal megafauna in a polymetallic-nodule region in the eastern Clarion-Clipperton Zone. Scientific Reports. 2016;6. doi: 10.1038/srep30492; Leitner A, Neuheimer A, Donlon E, Smith CR, Drazen JC. Environmental and bathymetric influences on abyssal bait-attending communities of the Clarion Clipperton Zone. Deep Sea Research Part I: Oceanographic Research Papers. 2017. doi: 10.1016/j.dsr.2017.04.017; **Taxon:** taxonConceptID: cf. *Coryphaenoides* morphospecies; scientificName: *Coryphaenoides* sp.; kingdom: Animalia; phylum: Chordata; class: Actinopterygii; order: Gadiformes; family: Macrouridae; genus: Coryphaenoides; taxonRank: genus; scientificNameAuthorship: Gunnerus, 1765; **Location:** waterBody: Pacific Ocean; stateProvince: Clarion-Clipperton Zone; locality: UK Seabed Resources Ltd exploration contract area (UK-1); verbatimLocality: UK-1 Stratum B; maximumDepthInMeters: 4221; locationRemarks: RV Thompson Cruise TN319; decimalLatitude: 12.5877; decimalLongitude: -116.7179; geodeticDatum: WGS84; coordinateUncertaintyInMeters: 25; **Identification:** identifiedBy: Astrid Leitner, Jeffrey Drazen, Diva J. Amon, Amanda F. Ziegler; dateIdentified: 2015; identificationRemarks: Identified only from imagery; identificationQualifier: cf.; **Event:** samplingProtocol: Autonomous Underwater Vehicle; eventDate: 2015-03-09; eventTime: 8:31; habitat: Abyssal polymetallic-nodule field; fieldNumber: Dive 6 (AV06); **Record Level:** language: en; institutionCode: UHM; datasetName: ABYSSLINE; basisOfRecord: HumanObservation
- **Type status:** Other material. **Occurrence:** catalogNumber: AB1-TR05-fish-1; recordNumber: AB1-TR05-fish-1; recordedBy: Jeffrey Drazen, Astrid Leitner; individualCount: 1; lifeStage: Adult; occurrenceStatus: present; preparations: tissue and DNA voucher stored in 80% non-denatured ethanol aqueous solution and remainder of animal preserved in 4% formaldehyde; associatedReferences: Amon DJ, Ziegler AF, Dahlgren TG, Glover AG, Goineau A, Gooday AJ, Wiklund H, Smith CR. Insights into the abundance and diversity of abyssal megafauna in a polymetallic-nodule region in the eastern Clarion-Clipperton Zone. Scientific Reports. 2016;6. doi: 10.1038/srep30492; Leitner A, Neuheimer A, Donlon E, Smith CR, Drazen JC. Environmental and bathymetric influences on abyssal bait-attending communities of the Clarion Clipperton Zone. Deep Sea Research Part I: Oceanographic Research Papers. 2017. doi: 10.1016/j.dsr.2017.04.017; **Taxon:** taxonConceptID: Coryphaenoides yaquinae; scientificName: Coryphaenoides yaquinae; kingdom: Animalia; phylum: Chordata; class: Actinopterygii; order: Gadiformes; family: Macrouridae; genus: Coryphaenoides; taxonRank: species; scientificNameAuthorship: Iwamoto & Stein, 1974; **Location:** waterBody: Pacific Ocean; stateProvince: Clarion-Clipperton Zone; locality: UK Seabed Resources Ltd exploration contract area (UK-1); verbatimLocality: UK-1 Stratum A; maximumDepthInMeters: 4156; locationRemarks: R/V Melville Cruise MV1313; decimalLatitude: 13.8885; decimalLongitude: -116.6824; geodeticDatum: WGS84; coordinateUncertaintyInMeters: 50; **Identification:** identifiedBy: Astrid Leitner, Jeffrey Drazen, Diva J. Amon, Amanda F. Ziegler; dateIdentified: 2014; identificationRemarks: Identified by morphology and DNA of collected specimen; **Event:** samplingProtocol: Baited Trap; eventDate: 2013-10-19; eventTime: 4:40; habitat: Abyssal polymetallic-nodule field; fieldNumber: TR05; **Record Level:** language: en; institutionCode: UHM; datasetName: ABYSSLINE; basisOfRecord: PreservedSpecimen
- **Type status:** Other material. **Occurrence:** catalogNumber: AB02-TR08-JD-61; recordNumber: AB02-TR08-JD-61; recordedBy: Jeffrey Drazen, Astrid Leitner; individualCount: 1; lifeStage: Adult; occurrenceStatus: present; preparations: tissue and DNA voucher stored in 80% non-denatured ethanol aqueous solution and remainder of animal preserved in 4% formaldehyde; associatedReferences: Amon DJ, Ziegler AF, Dahlgren TG, Glover AG, Goineau A, Gooday AJ, Wiklund H, Smith CR. Insights into the abundance and diversity of abyssal megafauna in a polymetallic-nodule region in the eastern Clarion-Clipperton Zone. Scientific Reports. 2016;6. doi: 10.1038/srep30492; Leitner A, Neuheimer A, Donlon E, Smith CR, Drazen JC. Environmental and bathymetric influences on abyssal bait-attending communities of the Clarion Clipperton Zone. Deep Sea Research Part I: Oceanographic Research Papers. 2017. doi: 10.1016/j.dsr.2017.04.017; **Taxon:** taxonConceptID: Coryphaenoides armatus; scientificName: Coryphaenoides armatus; kingdom: Animalia; phylum: Chordata; class: Actinopterygii; order: Gadiformes; family: Macrouridae; genus: Coryphaenoides; taxonRank: species; scientificNameAuthorship: Hector, 1875; **Location:** waterBody: Pacific Ocean; stateProvince: Clarion-Clipperton Zone; locality: UK Seabed Resources Ltd exploration contract area (UK-1); verbatimLocality: UK-1 Stratum A; maximumDepthInMeters: 4152; locationRemarks: RV Thompson Cruise TN319; decimalLatitude: 12.4435; decimalLongitude: -116.2074; geodeticDatum: WGS84; coordinateUncertaintyInMeters: 50; **Identification:** identifiedBy: Astrid Leitner, Jeffrey Drazen, Diva J. Amon, Amanda F. Ziegler; dateIdentified: 2015; identificationRemarks: Identified by morphology and DNA of collected specimen; **Event:** samplingProtocol: Baited Trap; eventDate: 2015-03-07; eventTime: 17:28; habitat: Abyssal polymetallic-nodule field; fieldNumber: TR08; **Record Level:** language: en; institutionCode: UHM; datasetName: ABYSSLINE; basisOfRecord: PreservedSpecimen
- **Type status:** Other material. **Occurrence:** recordedBy: Jeffrey Drazen, Astrid Leitner; individualCount: 4; lifeStage: Adult; behavior: Swimming; occurrenceStatus: present; preparations: Imaged only; associatedReferences: Amon DJ, Ziegler AF, Dahlgren TG, Glover AG, Goineau A, Gooday AJ, Wiklund H, Smith CR. Insights into the abundance and diversity of abyssal megafauna in a polymetallic-nodule region in the eastern Clarion-Clipperton Zone. Scientific Reports. 2016;6. doi: 10.1038/srep30492; Leitner A, Neuheimer A, Donlon E, Smith CR, Drazen JC. Environmental and bathymetric influences on abyssal bait-attending communities of the Clarion Clipperton Zone. Deep Sea Research Part I: Oceanographic Research Papers. 2017. doi: 10.1016/j.dsr.2017.04.017; **Taxon:** taxonConceptID: cf. *Coryphaenoides* morphospecies; scientificName: *Coryphaenoides* sp.; kingdom: Animalia; phylum: Chordata; class: Actinopterygii; order: Gadiformes; family: Macrouridae; genus: Coryphaenoides; taxonRank: genus; scientificNameAuthorship: Gunnerus, 1765; **Location:** waterBody: Pacific Ocean; stateProvince: Clarion-Clipperton Zone; locality: UK Seabed Resources Ltd exploration contract area (UK-1); verbatimLocality: UK-1 Stratum B; maximumDepthInMeters: 4275; locationRemarks: RV Thompson Cruise TN319; decimalLatitude: 12.5127; decimalLongitude: -116.6219; geodeticDatum: WGS84; coordinateUncertaintyInMeters: 50; **Identification:** identifiedBy: Astrid Leitner, Jeffrey Drazen, Diva J. Amon, Amanda F. Ziegler; dateIdentified: 2015; identificationRemarks: Identified only from imagery; identificationQualifier: cf.; **Event:** samplingProtocol: Baited Camera; eventDate: 2015-03-03; eventTime: 21:13; habitat: Abyssal polymetallic-nodule field; fieldNumber: CA06; **Record Level:** language: en; institutionCode: UHM; datasetName: ABYSSLINE; basisOfRecord: HumanObservation

##### Notes

Includes both species *C. armatus* and *C. yaquinae.* Both species have been recovered from the CCZ but are not readily distinguishable in photographs. Prominent snout and small chin barbel. First dorsal fin prominent and separated from the second dorsal fin which tapers to tip of whip tail. First pelvic fin ray elongated. Body color ranges from white to dark gray often with darker areas around fin insertions and eyes.

Fig. 35

#### Notacanthiformes

#### Halosauridae

Günther, 1868

#### cf. Halosauridae morphospecies

cf. Halosauridae morphospecies In the “Atlas of Abyssal Megafauna Morphotypes of the Clarion-Clipperton Fracture Zone” created for the ISA (http://ccfzatlas.com/), this morphospecies is listed as "Halosaur morphotype".

##### Materials

- **Type status:** Other material. **Occurrence:** recordedBy: Diva J. Amon, Amanda F. Ziegler; individualCount: 1; lifeStage: Adult; behavior: Swimming; occurrenceStatus: present; preparations: Imaged only; **Taxon:** taxonConceptID: cf. Halosauridae morphospecies; scientificName: Halosauridae sp.; kingdom: Animalia; phylum: Chordata; class: Actinopterygii; order: Notacanthiformes; family: Halosauridae; taxonRank: family; scientificNameAuthorship: Günther, 1868; **Location:** waterBody: Pacific Ocean; stateProvince: Clarion-Clipperton Zone; locality: UK Seabed Resources Ltd exploration contract area (UK-1); verbatimLocality: UK-1 Stratum B; maximumDepthInMeters: 4224; locationRemarks: RV Thompson Cruise TN319; decimalLatitude: 12.5850; decimalLongitude: -116.7021; geodeticDatum: WGS84; coordinateUncertaintyInMeters: 25; **Identification:** identifiedBy: Astrid Leitner, Jeffrey Drazen, Diva J. Amon, Amanda F. Ziegler; dateIdentified: 2015; identificationRemarks: Identified only from imagery; identificationQualifier: cf.; **Event:** samplingProtocol: Autonomous Underwater Vehicle; eventDate: 2015-03-09; eventTime: 5:38; habitat: Abyssal polymetallic-nodule field; fieldNumber: Dive 6 (AV06); **Record Level:** language: en; institutionCode: UHM; datasetName: ABYSSLINE; basisOfRecord: HumanObservation
- **Type status:** Other material. **Occurrence:** recordedBy: Diva J. Amon, Amanda F. Ziegler; individualCount: 1; lifeStage: Adult; behavior: Swimming; occurrenceStatus: present; preparations: Imaged only; **Taxon:** taxonConceptID: cf. Halosauridae morphospecies; scientificName: Halosauridae sp.; kingdom: Animalia; phylum: Chordata; class: Actinopterygii; order: Notacanthiformes; family: Halosauridae; taxonRank: family; scientificNameAuthorship: Günther, 1868; **Location:** waterBody: Pacific Ocean; stateProvince: Clarion-Clipperton Zone; locality: UK Seabed Resources Ltd exploration contract area (UK-1); verbatimLocality: UK-1 Stratum B; maximumDepthInMeters: 4213; locationRemarks: RV Thompson Cruise TN319; decimalLatitude: 12.5823; decimalLongitude: -116.7109; geodeticDatum: WGS84; coordinateUncertaintyInMeters: 25; **Identification:** identifiedBy: Astrid Leitner, Jeffrey Drazen, Diva J. Amon, Amanda F. Ziegler; dateIdentified: 2015; identificationRemarks: Identified only from imagery; identificationQualifier: cf.; **Event:** samplingProtocol: Autonomous Underwater Vehicle; eventDate: 2015-03-09; eventTime: 14:41; habitat: Abyssal polymetallic-nodule field; fieldNumber: Dive 6 (AV06); **Record Level:** language: en; institutionCode: UHM; datasetName: ABYSSLINE; basisOfRecord: HumanObservation

##### Notes

Very slender, long body with slender, pointed head. Long whip-like tail. Head often slightly darker than rest of body. Colors range from white to blue/grey. Distinguishable by swimming pattern: majority of slender body nearly completely straight with propulsion driven by flexion of terminal ¼ to ½ of body.

Fig. 36

#### Ophidiiformes

#### Ophidiidae

Rafinesque, 1810

#### cf. Ophidiidae morphospecies 1

cf. Ophidiidae morphospecies 1 In the “Atlas of Abyssal Megafauna Morphotypes of the Clarion-Clipperton Fracture Zone” created for the ISA (http://ccfzatlas.com/), this morphospecies is listed as "Ophidiidae morphotype 1".

##### Materials

- **Type status:** Other material. **Occurrence:** recordedBy: Diva J. Amon, Amanda F. Ziegler; individualCount: 1; lifeStage: Adult; behavior: Swimming; occurrenceStatus: present; preparations: Imaged only; associatedReferences: Amon DJ, Ziegler AF, Dahlgren TG, Glover AG, Goineau A, Gooday AJ, Wiklund H, Smith CR. Insights into the abundance and diversity of abyssal megafauna in a polymetallic-nodule region in the eastern Clarion-Clipperton Zone. Scientific Reports. 2016;6. doi: 10.1038/srep30492; **Taxon:** taxonConceptID: cf. Ophidiidae morphospecies 1; scientificName: Ophidiidae sp.; kingdom: Animalia; phylum: Chordata; class: Actinopterygii; order: Ophidiiformes; family: Ophidiidae; taxonRank: family; scientificNameAuthorship: Rafinesque, 1810; **Location:** waterBody: Pacific Ocean; stateProvince: Clarion-Clipperton Zone; locality: UK Seabed Resources Ltd exploration contract area (UK-1); verbatimLocality: UK-1 Stratum B; maximumDepthInMeters: 4234; locationRemarks: RV Thompson Cruise TN319; decimalLatitude: 12.5389; decimalLongitude: -116.6055; geodeticDatum: WGS84; coordinateUncertaintyInMeters: 25; **Identification:** identifiedBy: Astrid Leitner, Jeffrey Drazen, Diva J. Amon, Amanda F. Ziegler; dateIdentified: 2015; identificationRemarks: Identified only from imagery; identificationQualifier: cf.; **Event:** samplingProtocol: Autonomous Underwater Vehicle; eventDate: 2015-03-18; eventTime: 15:51; habitat: Abyssal polymetallic-nodule field; fieldNumber: Dive 9 (AV09); **Record Level:** language: en; institutionCode: UHM; datasetName: ABYSSLINE; basisOfRecord: HumanObservation
- **Type status:** Other material. **Occurrence:** recordedBy: Diva J. Amon, Amanda F. Ziegler; individualCount: 1; lifeStage: Adult; behavior: Swimming; occurrenceStatus: present; preparations: Imaged only; associatedReferences: Amon DJ, Ziegler AF, Dahlgren TG, Glover AG, Goineau A, Gooday AJ, Wiklund H, Smith CR. Insights into the abundance and diversity of abyssal megafauna in a polymetallic-nodule region in the eastern Clarion-Clipperton Zone. Scientific Reports. 2016;6. doi: 10.1038/srep30492; **Taxon:** taxonConceptID: cf. Ophidiidae morphospecies 1; scientificName: Ophidiidae sp.; kingdom: Animalia; phylum: Chordata; class: Actinopterygii; order: Ophidiiformes; family: Ophidiidae; taxonRank: family; scientificNameAuthorship: Rafinesque, 1810; **Location:** waterBody: Pacific Ocean; stateProvince: Clarion-Clipperton Zone; locality: UK Seabed Resources Ltd exploration contract area (UK-1); verbatimLocality: UK-1 Stratum A; maximumDepthInMeters: 4026; locationRemarks: RV Melville Cruise MV1313; decimalLatitude: 13.8643; decimalLongitude: -116.5486; geodeticDatum: WGS84; coordinateUncertaintyInMeters: 25; **Identification:** identifiedBy: Astrid Leitner, Jeffrey Drazen, Diva J. Amon, Amanda F. Ziegler; dateIdentified: 2014; identificationRemarks: Identified only from imagery; identificationQualifier: cf.; **Event:** samplingProtocol: Remotely Operated Vehicle; eventDate: 2013-10-21; eventTime: 5:18; habitat: Abyssal polymetallic-nodule field; fieldNumber: Dive 6 (RV06); **Record Level:** language: en; institutionCode: UHM; datasetName: ABYSSLINE; basisOfRecord: HumanObservation

##### Notes

A single dorsal fin begins in line with the pectoral fins and continues full length of body. Angular, near rectangular snout.

Fig. 37

#### cf. Ophidiidae morphospecies 2

##### Materials

- **Type status:** Other material. **Occurrence:** recordedBy: Jeffrey Drazen, Astrid Leitner; individualCount: 1; lifeStage: Adult; behavior: On seafloor; occurrenceStatus: present; preparations: Imaged only; associatedReferences: Leitner A, Neuheimer A, Donlon E, Smith CR, Drazen JC. Environmental and bathymetric influences on abyssal bait-attending communities of the Clarion Clipperton Zone. Deep Sea Research Part I: Oceanographic Research Papers. 2017. doi: 10.1016/j.dsr.2017.04.017; **Taxon:** taxonConceptID: cf. Ophidiidae morphospecies 2; scientificName: Ophidiidae sp.; kingdom: Animalia; phylum: Chordata; class: Actinopterygii; order: Ophidiiformes; family: Ophidiidae; taxonRank: family; scientificNameAuthorship: Rafinesque, 1810; **Location:** waterBody: Pacific Ocean; stateProvince: Clarion-Clipperton Zone; locality: UK Seabed Resources Ltd exploration contract area (UK-1); verbatimLocality: UK-1 Stratum B; maximumDepthInMeters: 3605; locationRemarks: RV Thompson Cruise TN319; decimalLatitude: 12.4353; decimalLongitude: -116.5533; geodeticDatum: WGS84; coordinateUncertaintyInMeters: 50; **Identification:** identifiedBy: Astrid Leitner, Jeffrey Drazen, Diva J. Amon, Amanda F. Ziegler; dateIdentified: 2015; identificationRemarks: Identified only from imagery; identificationQualifier: cf.; **Event:** samplingProtocol: Baited Camera; eventDate: 2015-03-09; eventTime: 19:15; habitat: Abyssal polymetallic-nodule field; fieldNumber: CA09; **Record Level:** language: en; institutionCode: UHM; datasetName: ABYSSLINE; basisOfRecord: HumanObservation

##### Notes

Angular head with lighter coloration along lower jaw. Rectangular body shape that maintains near maximum body depth for most of standard length. Dark contiguous dorsal and anal fins. Observed to drag body over sediment and leave a trail.

Fig. 38

#### cf. Ophidiidae morphospecies 3

cf. Ophidiidae morphospecies 3 In the “Atlas of Abyssal Megafauna Morphotypes of the Clarion-Clipperton Fracture Zone” created for the ISA (http://ccfzatlas.com/), this morphospecies is listed as "Ophidiidae morphotype 3".

##### Materials

- **Type status:** Other material. **Occurrence:** recordedBy: Diva J. Amon, Amanda F. Ziegler; individualCount: 1; lifeStage: Adult; behavior: Swimming; occurrenceStatus: present; preparations: Imaged only; **Taxon:** taxonConceptID: cf. Ophidiidae morphospecies 3; scientificName: Ophidiidae sp.; kingdom: Animalia; phylum: Chordata; class: Actinopterygii; order: Ophidiiformes; family: Ophidiidae; taxonRank: family; scientificNameAuthorship: Rafinesque, 1810; **Location:** waterBody: Pacific Ocean; stateProvince: Clarion-Clipperton Zone; locality: UK Seabed Resources Ltd exploration contract area (UK-1); verbatimLocality: UK-1 Stratum B; maximumDepthInMeters: 4248; locationRemarks: RV Thompson Cruise TN319; decimalLatitude: 12.4848; decimalLongitude: -116.6549; geodeticDatum: WGS84; coordinateUncertaintyInMeters: 25; **Identification:** identifiedBy: Astrid Leitner, Jeffrey Drazen, Diva J. Amon, Amanda F. Ziegler; dateIdentified: 2015; identificationRemarks: Identified only from imagery; identificationQualifier: cf.; **Event:** samplingProtocol: Autonomous Underwater Vehicle; eventDate: 2015-03-04; eventTime: 10:12; habitat: Abyssal polymetallic-nodule field; fieldNumber: Dive 5 (AV05); **Record Level:** language: en; institutionCode: UHM; datasetName: ABYSSLINE; basisOfRecord: HumanObservation

##### Notes

Slender fish with large and distinctive pectoral fins. Usually a grey in colour.

Fig. 39

#### cf. Ophidiidae morphospecies 4

##### Materials

- **Type status:** Other material. **Occurrence:** recordedBy: Diva J. Amon, Amanda F. Ziegler; individualCount: 1; lifeStage: Adult; behavior: Swimming; occurrenceStatus: present; preparations: Imaged only; **Taxon:** taxonConceptID: cf. Ophidiidae morphospecies 4; scientificName: Ophidiidae sp.; kingdom: Animalia; phylum: Chordata; class: Actinopterygii; order: Ophidiiformes; family: Ophidiidae; taxonRank: family; scientificNameAuthorship: Rafinesque, 1810; **Location:** waterBody: Pacific Ocean; stateProvince: Clarion-Clipperton Zone; locality: UK Seabed Resources Ltd exploration contract area (UK-1); verbatimLocality: UK-1 Stratum B; maximumDepthInMeters: 4251; locationRemarks: RV Thompson Cruise TN319; decimalLatitude: 12.4957; decimalLongitude: -116.6505; geodeticDatum: WGS84; coordinateUncertaintyInMeters: 25; **Identification:** identifiedBy: Astrid Leitner, Jeffrey Drazen, Diva J. Amon, Amanda F. Ziegler; dateIdentified: 2015; identificationRemarks: Identified only from imagery; identificationQualifier: cf.; **Event:** samplingProtocol: Autonomous Underwater Vehicle; eventDate: 2015-03-04; eventTime: 1:09; habitat: Abyssal polymetallic-nodule field; fieldNumber: Dive 5 (AV05); **Record Level:** language: en; institutionCode: UHM; datasetName: ABYSSLINE; basisOfRecord: HumanObservation
- **Type status:** Other material. **Occurrence:** recordedBy: Diva J. Amon, Amanda F. Ziegler; individualCount: 1; lifeStage: Adult; behavior: Swimming; occurrenceStatus: present; preparations: Imaged only; **Taxon:** taxonConceptID: cf. Ophidiidae morphospecies 4; scientificName: Ophidiidae sp.; kingdom: Animalia; phylum: Chordata; class: Actinopterygii; order: Ophidiiformes; family: Ophidiidae; taxonRank: family; scientificNameAuthorship: Rafinesque, 1810; **Location:** waterBody: Pacific Ocean; stateProvince: Clarion-Clipperton Zone; locality: UK Seabed Resources Ltd exploration contract area (UK-1); verbatimLocality: UK-1 Stratum B; maximumDepthInMeters: 4211; locationRemarks: RV Thompson Cruise TN319; decimalLatitude: 12.5726; decimalLongitude: -116.7342; geodeticDatum: WGS84; coordinateUncertaintyInMeters: 25; **Identification:** identifiedBy: Astrid Leitner, Jeffrey Drazen, Diva J. Amon, Amanda F. Ziegler; dateIdentified: 2015; identificationRemarks: Identified only from imagery; identificationQualifier: cf.; **Event:** samplingProtocol: Autonomous Underwater Vehicle; eventDate: 2015-03-09; eventTime: 7:43; habitat: Abyssal polymetallic-nodule field; fieldNumber: Dive 6 (AV06); **Record Level:** language: en; institutionCode: UHM; datasetName: ABYSSLINE; basisOfRecord: HumanObservation

##### Notes

Rounded bulbous head. Blue/purple to brown coloration.

Fig. 40

#### cf. Ophidiidae morphospecies 5

##### Materials

- **Type status:** Other material. **Occurrence:** recordedBy: Diva J. Amon, Amanda F. Ziegler; individualCount: 1; lifeStage: Adult; behavior: Swimming; occurrenceStatus: present; preparations: Imaged only; **Taxon:** taxonConceptID: cf. Ophidiidae morphospecies 5; scientificName: Ophidiidae sp.; kingdom: Animalia; phylum: Chordata; class: Actinopterygii; order: Ophidiiformes; family: Ophidiidae; taxonRank: family; scientificNameAuthorship: Rafinesque, 1810; **Location:** waterBody: Pacific Ocean; stateProvince: Clarion-Clipperton Zone; locality: UK Seabed Resources Ltd exploration contract area (UK-1); verbatimLocality: UK-1 Stratum B; maximumDepthInMeters: 4161; locationRemarks: RV Thompson Cruise TN319; decimalLatitude: 12.3681; decimalLongitude: -116.5187; geodeticDatum: WGS84; coordinateUncertaintyInMeters: 25; **Identification:** identifiedBy: Astrid Leitner, Jeffrey Drazen, Diva J. Amon, Amanda F. Ziegler; dateIdentified: 2015; identificationRemarks: Identified only from imagery; identificationQualifier: cf.; **Event:** samplingProtocol: Autonomous Underwater Vehicle; eventDate: 2015-02-18; eventTime: 16:22; habitat: Abyssal polymetallic-nodule field; fieldNumber: Dive 1 (AV01); **Record Level:** language: en; institutionCode: UHM; datasetName: ABYSSLINE; basisOfRecord: HumanObservation
- **Type status:** Other material. **Occurrence:** recordedBy: Diva J. Amon, Amanda F. Ziegler; individualCount: 1; lifeStage: Adult; behavior: Swimming; occurrenceStatus: present; preparations: Imaged only; **Taxon:** taxonConceptID: cf. Ophidiidae morphospecies 5; scientificName: Ophidiidae sp.; kingdom: Animalia; phylum: Chordata; class: Actinopterygii; order: Ophidiiformes; family: Ophidiidae; taxonRank: family; scientificNameAuthorship: Rafinesque, 1810; **Location:** waterBody: Pacific Ocean; stateProvince: Clarion-Clipperton Zone; locality: UK Seabed Resources Ltd exploration contract area (UK-1); verbatimLocality: UK-1 Stratum B; maximumDepthInMeters: 4229; locationRemarks: RV Thompson Cruise TN319; decimalLatitude: 12.5793; decimalLongitude: -116.7205; geodeticDatum: WGS84; coordinateUncertaintyInMeters: 25; **Identification:** identifiedBy: Astrid Leitner, Jeffrey Drazen, Diva J. Amon, Amanda F. Ziegler; dateIdentified: 2015; identificationRemarks: Identified only from imagery; identificationQualifier: cf.; **Event:** samplingProtocol: Autonomous Underwater Vehicle; eventDate: 2015-03-09; eventTime: 6:01; habitat: Abyssal polymetallic-nodule field; fieldNumber: Dive 6 (AV06); **Record Level:** language: en; institutionCode: UHM; datasetName: ABYSSLINE; basisOfRecord: HumanObservation

##### Notes

Bright white coloration. Small head. Dorsal fin insertion at half body length.

Fig. 41

#### Barathrites

Zugmayer, 1911

#### Barathrites cf. iris

Zugmayer, 1911

Barathrites cf. iris In the “Atlas of Abyssal Megafauna Morphotypes of the Clarion-Clipperton Fracture Zone” created for the ISA (http://ccfzatlas.com/), this morphospecies is listed as "Ophidiidae morphotype 2".

##### Materials

- **Type status:** Other material. **Occurrence:** recordedBy: Jeffrey Drazen, Astrid Leitner; individualCount: 1; lifeStage: Adult; behavior: Swimming; occurrenceStatus: present; preparations: Imaged only; associatedReferences: Leitner A, Neuheimer A, Donlon E, Smith CR, Drazen JC. Environmental and bathymetric influences on abyssal bait-attending communities of the Clarion Clipperton Zone. Deep Sea Research Part I: Oceanographic Research Papers. 2017. doi: 10.1016/j.dsr.2017.05; **Taxon:** taxonConceptID: Barathrites cf. iris; scientificName: Barathrites iris; kingdom: Animalia; phylum: Chordata; class: Actinopterygii; order: Ophidiiformes; family: Ophidiidae; genus: Barathrites; taxonRank: species; scientificNameAuthorship: Zugmayer, 1911; **Location:** waterBody: Pacific Ocean; stateProvince: Clarion-Clipperton Zone; locality: UK Seabed Resources Ltd exploration contract area (UK-1); verbatimLocality: UK-1 Stratum B; maximumDepthInMeters: 4212; locationRemarks: RV Thompson Cruise TN319; decimalLatitude: 12.5126; decimalLongitude: -116.6224; geodeticDatum: WGS84; coordinateUncertaintyInMeters: 50; **Identification:** identifiedBy: Astrid Leitner, Jeffrey Drazen, Diva J. Amon, Amanda F. Ziegler; dateIdentified: 2015; identificationRemarks: Identified only from imagery; identificationQualifier: cf.; **Event:** samplingProtocol: Baited Camera; eventDate: 2015-03-03; eventTime: 21:13; habitat: Abyssal polymetallic-nodule field; fieldNumber: CA06; **Record Level:** language: en; institutionCode: UHM; datasetName: ABYSSLINE; basisOfRecord: HumanObservation

##### Notes

Often bright white or mottled white/brown. Head small with distinct snout and curved downturned mouth. Relatively deep-bodied. Pelvic fins reduced to two rays each and under head forward of gill slit. Readily distinguished from other ophidiids by head morphology and body depth.

Fig. 42

#### Bassozetus

Gill, 1883

#### Bassozetus cf. nasus

Garman, 1899

Bassozetus cf. nasus In the “Atlas of Abyssal Megafauna Morphotypes of the Clarion-Clipperton Fracture Zone” created for the ISA (http://ccfzatlas.com/), this morphospecies is listed as "Ophidiidae morphotype 4".

##### Materials

- **Type status:** Other material. **Occurrence:** recordedBy: Diva J. Amon, Amanda F. Ziegler; individualCount: 1; lifeStage: Adult; behavior: Swimming; occurrenceStatus: present; preparations: Imaged only; associatedReferences: Amon DJ, Ziegler AF, Dahlgren TG, Glover AG, Goineau A, Gooday AJ, Wiklund H, Smith CR. Insights into the abundance and diversity of abyssal megafauna in a polymetallic-nodule region in the eastern Clarion-Clipperton Zone. Scientific Reports. 2016;6. doi: 10.1038/srep30492; Leitner A, Neuheimer A, Donlon E, Smith CR, Drazen JC. Environmental and bathymetric influences on abyssal bait-attending communities of the Clarion Clipperton Zone. Deep Sea Research Part I: Oceanographic Research Papers. 2017. doi: 10.1016/j.dsr.2017.04.017; **Taxon:** taxonConceptID: Bassozetus cf. nasus; scientificName: Bassozetus nasus; kingdom: Animalia; phylum: Chordata; class: Actinopterygii; order: Ophidiiformes; family: Ophidiidae; genus: Bassozetus; taxonRank: species; scientificNameAuthorship: Garman, 1899; **Location:** waterBody: Pacific Ocean; stateProvince: Clarion-Clipperton Zone; locality: UK Seabed Resources Ltd exploration contract area (UK-1); verbatimLocality: UK-1 Stratum A; maximumDepthInMeters: 4070; locationRemarks: RV Melville Cruise MV1313; decimalLatitude: 13.7603; decimalLongitude: -116.4677; geodeticDatum: WGS84; coordinateUncertaintyInMeters: 25; **Identification:** identifiedBy: Astrid Leitner, Jeffrey Drazen, Diva J. Amon, Amanda F. Ziegler; dateIdentified: 2014; identificationRemarks: Identified only from imagery; identificationQualifier: cf.; **Event:** samplingProtocol: Remotely Operated Vehicle; eventDate: 2013-10-18; eventTime: 2:16; habitat: Abyssal polymetallic-nodule field; fieldNumber: Dive 5 (RV05); **Record Level:** language: en; institutionCode: UHM; datasetName: ABYSSLINE; basisOfRecord: HumanObservation
- **Type status:** Other material. **Occurrence:** recordedBy: Diva J. Amon, Amanda F. Ziegler; individualCount: 1; lifeStage: Adult; behavior: Swimming; occurrenceStatus: present; preparations: Imaged only; associatedReferences: Amon DJ, Ziegler AF, Dahlgren TG, Glover AG, Goineau A, Gooday AJ, Wiklund H, Smith CR. Insights into the abundance and diversity of abyssal megafauna in a polymetallic-nodule region in the eastern Clarion-Clipperton Zone. Scientific Reports. 2016;6. doi: 10.1038/srep30492; Leitner A, Neuheimer A, Donlon E, Smith CR, Drazen JC. Environmental and bathymetric influences on abyssal bait-attending communities of the Clarion Clipperton Zone. Deep Sea Research Part I: Oceanographic Research Papers. 2017. doi: 10.1016/j.dsr.2017.04.017; **Taxon:** taxonConceptID: Bassozetus cf. nasus; scientificName: Bassozetus nasus; kingdom: Animalia; phylum: Chordata; class: Actinopterygii; order: Ophidiiformes; family: Ophidiidae; genus: Bassozetus; taxonRank: species; scientificNameAuthorship: Garman, 1899; **Location:** waterBody: Pacific Ocean; stateProvince: Clarion-Clipperton Zone; locality: UK Seabed Resources Ltd exploration contract area (UK-1); verbatimLocality: UK-1 Stratum B; maximumDepthInMeters: 4225; locationRemarks: RV Thompson Cruise TN319; decimalLatitude: 12.5778; decimalLongitude: -116.7241; geodeticDatum: WGS84; coordinateUncertaintyInMeters: 25; **Identification:** identifiedBy: Astrid Leitner, Jeffrey Drazen, Diva J. Amon, Amanda F. Ziegler; dateIdentified: 2015; identificationRemarks: Identified only from imagery; identificationQualifier: cf.; **Event:** samplingProtocol: Autonomous Underwater Vehicle; eventDate: 2015-03-09; eventTime: 11:50; habitat: Abyssal polymetallic-nodule field; fieldNumber: Dive 6 (AV06); **Record Level:** language: en; institutionCode: UHM; datasetName: ABYSSLINE; basisOfRecord: HumanObservation
- **Type status:** Other material. **Occurrence:** recordedBy: Jeffrey Drazen, Astrid Leitner; individualCount: 1; lifeStage: Adult; behavior: Swimming; occurrenceStatus: present; preparations: Imaged only; associatedReferences: Amon DJ, Ziegler AF, Dahlgren TG, Glover AG, Goineau A, Gooday AJ, Wiklund H, Smith CR. Insights into the abundance and diversity of abyssal megafauna in a polymetallic-nodule region in the eastern Clarion-Clipperton Zone. Scientific Reports. 2016;6. doi: 10.1038/srep30492; Leitner A, Neuheimer A, Donlon E, Smith CR, Drazen JC. Environmental and bathymetric influences on abyssal bait-attending communities of the Clarion Clipperton Zone. Deep Sea Research Part I: Oceanographic Research Papers. 2017. doi: 10.1016/j.dsr.2017.04.017; **Taxon:** taxonConceptID: Bassozetus cf. nasus; scientificName: Bassozetus nasus; kingdom: Animalia; phylum: Chordata; class: Actinopterygii; order: Ophidiiformes; family: Ophidiidae; genus: Bassozetus; taxonRank: species; scientificNameAuthorship: Garman, 1899; **Location:** waterBody: Pacific Ocean; stateProvince: Clarion-Clipperton Zone; locality: UK Seabed Resources Ltd exploration contract area (UK-1); verbatimLocality: UK-1 Stratum B; maximumDepthInMeters: 4312; locationRemarks: RV Thompson Cruise TN319; decimalLatitude: 12.5532; decimalLongitude: -116.5386; geodeticDatum: WGS84; coordinateUncertaintyInMeters: 50; **Identification:** identifiedBy: Astrid Leitner, Jeffrey Drazen, Diva J. Amon, Amanda F. Ziegler; dateIdentified: 2015; identificationRemarks: Identified only from imagery; identificationQualifier: cf.; **Event:** samplingProtocol: Baited Camera; eventDate: 2015-03-05; eventTime: 19:37; habitat: Abyssal polymetallic-nodule field; fieldNumber: CA07; **Record Level:** language: en; institutionCode: UHM; datasetName: ABYSSLINE; basisOfRecord: HumanObservation

##### Notes

Large round, bulbous head with a tadpole-like body morphology. Reduced eyes. A variety of colormorphs exist ranging from beige to dark brown. May have lighter spots. Single dorsal contiguous with anal fin. Pelvic fins reduced to one ray each and below head. Distinguished from other ophidiids by the large rounded head.

Fig. 43

#### Bathyonus

Goode & Bean, 1885

#### Bathyonus cf. caudalis

Garman, 1899

##### Materials

- **Type status:** Other material. **Occurrence:** recordedBy: Jeffrey Drazen, Astrid Leitner; individualCount: 1; lifeStage: Adult; behavior: Swimming; occurrenceStatus: present; preparations: Imaged only; associatedReferences: Leitner A, Neuheimer A, Donlon E, Smith CR, Drazen JC. Environmental and bathymetric influences on abyssal bait-attending communities of the Clarion Clipperton Zone. Deep Sea Research Part I: Oceanographic Research Papers. 2017. doi: 10.1016/j.dsr.2017.04.017; **Taxon:** taxonConceptID: Bathyonus cf. caudalis; scientificName: Bathyonus caudalis; kingdom: Animalia; phylum: Chordata; class: Actinopterygii; order: Ophidiiformes; family: Ophidiidae; genus: Bathyonus; taxonRank: species; scientificNameAuthorship: Garman, 1899; **Location:** waterBody: Pacific Ocean; stateProvince: Clarion-Clipperton Zone; locality: UK Seabed Resources Ltd exploration contract area (UK-1); verbatimLocality: UK-1 Stratum B; maximumDepthInMeters: 4212; locationRemarks: RV Thompson Cruise TN319; decimalLatitude: 12.5126; decimalLongitude: -116.6219; geodeticDatum: WGS84; coordinateUncertaintyInMeters: 50; **Identification:** identifiedBy: Astrid Leitner, Jeffrey Drazen, Diva J. Amon, Amanda F. Ziegler; dateIdentified: 2015; identificationRemarks: Identified only from imagery; identificationQualifier: cf.; **Event:** samplingProtocol: Baited Camera; eventDate: 2015-03-03; eventTime: 21:13; habitat: Abyssal polymetallic-nodule field; fieldNumber: CA06; **Record Level:** language: en; institutionCode: UHM; datasetName: ABYSSLINE; basisOfRecord: HumanObservation
- **Type status:** Other material. **Occurrence:** recordedBy: Diva J. Amon, Amanda F. Ziegler; individualCount: 1; lifeStage: Adult; behavior: Swimming; occurrenceStatus: present; preparations: Imaged only; associatedReferences: Leitner A, Neuheimer A, Donlon E, Smith CR, Drazen JC. Environmental and bathymetric influences on abyssal bait-attending communities of the Clarion Clipperton Zone. Deep Sea Research Part I: Oceanographic Research Papers. 2017. doi: 10.1016/j.dsr.2017.04.017; **Taxon:** taxonConceptID: Bathyonus cf. caudalis; scientificName: Bathyonus caudalis; kingdom: Animalia; phylum: Chordata; class: Actinopterygii; order: Ophidiiformes; family: Ophidiidae; genus: Bathyonus; taxonRank: species; scientificNameAuthorship: Garman, 1899; **Location:** waterBody: Pacific Ocean; stateProvince: Clarion-Clipperton Zone; locality: Eastern Clarion-Clipperton Zone; verbatimLocality: Site EPIRB; maximumDepthInMeters: 3956; locationRemarks: RV Melville Cruise MV1313; decimalLatitude: 13.6794; decimalLongitude: -114.4144; geodeticDatum: WGS84; coordinateUncertaintyInMeters: 25; **Identification:** identifiedBy: Astrid Leitner, Jeffrey Drazen, Diva J. Amon, Amanda F. Ziegler; dateIdentified: 2014; identificationRemarks: Identified only from imagery; identificationQualifier: cf.; **Event:** samplingProtocol: Remotely Operated Vehicle; eventDate: 2013-10-23; eventTime: 13:06; habitat: Abyssal polymetallic-nodule field; fieldNumber: Dive 7 (RV07); **Record Level:** language: en; institutionCode: UHM; datasetName: ABYSSLINE; basisOfRecord: HumanObservation

##### Notes

Very long tapered body. Usually golden brown in color. Lower rays of pectoral fins free and stronger than upper ones. No prominent spines on head. Pelvic fins reduced to two rays each and below head. Easily distinguished from other ophidiids by the large length to body depth ratio and the free pectoral fin rays.

Fig. 44

#### Perciformes

#### Zoarcidae

Swainson, 1839

#### cf. Zoarcidae morphospecies 1

##### Materials

- **Type status:** Other material. **Occurrence:** recordedBy: Jeffrey Drazen, Astrid Leitner; individualCount: 1; lifeStage: Adult; behavior: On seafloor; occurrenceStatus: present; preparations: Imaged only; associatedReferences: Leitner A, Neuheimer A, Donlon E, Smith CR, Drazen JC. Environmental and bathymetric influences on abyssal bait-attending communities of the Clarion Clipperton Zone. Deep Sea Research Part I: Oceanographic Research Papers. 2017. doi: 10.1016/j.dsr.2017.05; **Taxon:** taxonConceptID: cf. Zoarcidae morphospecies 1; scientificName: Zoarcidae sp.; kingdom: Animalia; phylum: Chordata; class: Actinopterygii; order: Perciformes; family: Zoarcidae; taxonRank: family; scientificNameAuthorship: Swainson, 1839; **Location:** waterBody: Pacific Ocean; stateProvince: Clarion-Clipperton Zone; locality: UK Seabed Resources Ltd exploration contract area (UK-1); verbatimLocality: UK-1 Stratum B; maximumDepthInMeters: 4236; locationRemarks: RV Thompson Cruise TN319; decimalLatitude: 12.4526; decimalLongitude: -116.6488; geodeticDatum: WGS84; coordinateUncertaintyInMeters: 50; **Identification:** identifiedBy: Astrid Leitner, Jeffrey Drazen, Diva J. Amon, Amanda F. Ziegler; dateIdentified: 2015; identificationRemarks: Identified only from imagery; identificationQualifier: cf.; **Event:** samplingProtocol: Baited Camera; eventDate: 2015-03-07; eventTime: 17:06; habitat: Abyssal polymetallic-nodule field; fieldNumber: CA08; **Record Level:** language: en; institutionCode: UHM; datasetName: ABYSSLINE; basisOfRecord: HumanObservation

##### Notes

White to grey stout, eel-like body. Small body size. Very rounded head. Usually adopts a curled body position. Distinguished by small size and head morphology.

Fig. 45

#### cf. Zoarcidae morphospecies 2

##### Materials

- **Type status:** Other material. **Occurrence:** recordedBy: Jeffrey Drazen, Astrid Leitner; individualCount: 1; lifeStage: Adult; behavior: On seafloor; occurrenceStatus: present; preparations: Imaged only; associatedReferences: Leitner A, Neuheimer A, Donlon E, Smith CR, Drazen JC. Environmental and bathymetric influences on abyssal bait-attending communities of the Clarion Clipperton Zone. Deep Sea Research Part I: Oceanographic Research Papers. 2017. doi: 10.1016/j.dsr.2017.05; **Taxon:** taxonConceptID: cf. Zoarcidae morphospecies 2; scientificName: Zoarcidae sp.; kingdom: Animalia; phylum: Chordata; class: Actinopterygii; order: Perciformes; family: Zoarcidae; taxonRank: family; scientificNameAuthorship: Swainson, 1839; **Location:** waterBody: Pacific Ocean; stateProvince: Clarion-Clipperton Zone; locality: UK Seabed Resources Ltd exploration contract area (UK-1); verbatimLocality: UK-1 Stratum B; maximumDepthInMeters: 4312; locationRemarks: RV Thompson Cruise TN319; decimalLatitude: 12.5532; decimalLongitude: -116.5586; geodeticDatum: WGS84; coordinateUncertaintyInMeters: 50; **Identification:** identifiedBy: Astrid Leitner, Jeffrey Drazen, Diva J. Amon, Amanda F. Ziegler; dateIdentified: 2015; identificationRemarks: Identified only from imagery; identificationQualifier: cf.; **Event:** samplingProtocol: Baited Camera; eventDate: 2015-03-05; eventTime: 19:37; habitat: Abyssal polymetallic-nodule field; fieldNumber: CA07; **Record Level:** language: en; institutionCode: UHM; datasetName: ABYSSLINE; basisOfRecord: HumanObservation

##### Notes

Stout eel-like body. Distinguished from *Pachycara nazca* by relatively more pointed, slender head and dark dorsal and anal fin edges.

Fig. 46

#### Pachycara

Zugmayer, 1911

#### Pachycara cf. nazca

Anderson & Bluhm, 1997

##### Materials

- **Type status:** Other material. **Occurrence:** recordedBy: Diva J. Amon, Amanda F. Ziegler; individualCount: 1; lifeStage: Adult; behavior: On seafloor; occurrenceStatus: present; preparations: Imaged only; associatedReferences: Amon DJ, Ziegler AF, Dahlgren TG, Glover AG, Goineau A, Gooday AJ, Wiklund H, Smith CR. Insights into the abundance and diversity of abyssal megafauna in a polymetallic-nodule region in the eastern Clarion-Clipperton Zone. Scientific Reports. 2016;6. doi: 10.1038/srep30492; Leitner A, Neuheimer A, Donlon E, Smith CR, Drazen JC. Environmental and bathymetric influences on abyssal bait-attending communities of the Clarion Clipperton Zone. Deep Sea Research Part I: Oceanographic Research Papers. 2017. doi: 10.1016/j.dsr.2017.04.017; **Taxon:** taxonConceptID: Pachycara cf. nazca; scientificName: Pachycara nazca; kingdom: Animalia; phylum: Chordata; class: Actinopterygii; order: Perciformes; family: Zoarcidae; genus: Pachycara; taxonRank: species; scientificNameAuthorship: Anderson & Bluhm, 1997; **Location:** waterBody: Pacific Ocean; stateProvince: Clarion-Clipperton Zone; locality: UK Seabed Resources Ltd exploration contract area (UK-1); verbatimLocality: UK-1 Stratum A; maximumDepthInMeters: 4070; locationRemarks: RV Melville Cruise MV1313; decimalLatitude: 13.7605; decimalLongitude: -116.4679; geodeticDatum: WGS84; coordinateUncertaintyInMeters: 25; **Identification:** identifiedBy: Astrid Leitner, Jeffrey Drazen, Diva J. Amon, Amanda F. Ziegler; dateIdentified: 2014; identificationRemarks: Identified only from imagery; identificationQualifier: cf.; **Event:** samplingProtocol: Remotely Operated Vehicle; eventDate: 2013-10-18; eventTime: 2:24; habitat: Abyssal polymetallic-nodule field; fieldNumber: Dive 5 (RV05); **Record Level:** language: en; institutionCode: UHM; datasetName: ABYSSLINE; basisOfRecord: HumanObservation
- **Type status:** Other material. **Occurrence:** catalogNumber: AB1-TR06-fish-1; recordNumber: AB1-TR06-fish-1; recordedBy: Jeffrey Drazen, Astrid Leitner; individualCount: 1; lifeStage: Adult; occurrenceStatus: present; preparations: tissue and DNA voucher stored in 80% non-denatured ethanol aqueous solution and remainder of animal preserved in 4% formaldehyde; associatedReferences: Amon DJ, Ziegler AF, Dahlgren TG, Glover AG, Goineau A, Gooday AJ, Wiklund H, Smith CR. Insights into the abundance and diversity of abyssal megafauna in a polymetallic-nodule region in the eastern Clarion-Clipperton Zone. Scientific Reports. 2016;6. doi: 10.1038/srep30492; Leitner A, Neuheimer A, Donlon E, Smith CR, Drazen JC. Environmental and bathymetric influences on abyssal bait-attending communities of the Clarion Clipperton Zone. Deep Sea Research Part I: Oceanographic Research Papers. 2017. doi: 10.1016/j.dsr.2017.04.017; **Taxon:** taxonConceptID: Pachycara nazca; scientificName: Pachycara nazca; kingdom: Animalia; phylum: Chordata; class: Actinopterygii; order: Perciformes; family: Zoarcidae; genus: Pachycara; taxonRank: species; scientificNameAuthorship: Anderson & Bluhm, 1997; **Location:** waterBody: Pacific Ocean; stateProvince: Clarion-Clipperton Zone; locality: UK Seabed Resources Ltd exploration contract area (UK-1); verbatimLocality: UK-1 Stratum A; maximumDepthInMeters: 4106; locationRemarks: RV Melville Cruise MV1313; decimalLatitude: 13.9022; decimalLongitude: -116.5742; geodeticDatum: WGS84; coordinateUncertaintyInMeters: 50; **Identification:** identifiedBy: Astrid Leitner, Jeffrey Drazen, Diva J. Amon, Amanda F. Ziegler, Eric Anderson; dateIdentified: 2014; identificationRemarks: Identified by morphology of collected specimen; **Event:** samplingProtocol: Baited Trap; eventDate: 2013-10-20; eventTime: 7:44; habitat: Abyssal polymetallic-nodule field; fieldNumber: TR06; **Record Level:** language: en; institutionCode: UHM; datasetName: ABYSSLINE; basisOfRecord: HumanObservation
- **Type status:** Other material. **Occurrence:** catalogNumber: AB1-TR06-fish-2; recordNumber: AB1-TR06-fish-2; recordedBy: Jeffrey Drazen, Astrid Leitner; individualCount: 1; lifeStage: Adult; occurrenceStatus: present; preparations: tissue and DNA voucher stored in 80% non-denatured ethanol aqueous solution and remainder of animal preserved in 4% formaldehyde; associatedReferences: Amon DJ, Ziegler AF, Dahlgren TG, Glover AG, Goineau A, Gooday AJ, Wiklund H, Smith CR. Insights into the abundance and diversity of abyssal megafauna in a polymetallic-nodule region in the eastern Clarion-Clipperton Zone. Scientific Reports. 2016;6. doi: 10.1038/srep30492; Leitner A, Neuheimer A, Donlon E, Smith CR, Drazen JC. Environmental and bathymetric influences on abyssal bait-attending communities of the Clarion Clipperton Zone. Deep Sea Research Part I: Oceanographic Research Papers. 2017. doi: 10.1016/j.dsr.2017.04.017; **Taxon:** taxonConceptID: Pachycara nazca; scientificName: Pachycara nazca; kingdom: Animalia; phylum: Chordata; class: Actinopterygii; order: Perciformes; family: Zoarcidae; genus: Pachycara; taxonRank: species; scientificNameAuthorship: Anderson & Bluhm, 1997; **Location:** waterBody: Pacific Ocean; stateProvince: Clarion-Clipperton Zone; locality: UK Seabed Resources Ltd exploration contract area (UK-1); verbatimLocality: UK-1 Stratum A; maximumDepthInMeters: 4106; locationRemarks: RV Melville Cruise MV1313; decimalLatitude: 13.9022; decimalLongitude: -116.5742; geodeticDatum: WGS84; coordinateUncertaintyInMeters: 50; **Identification:** identifiedBy: Astrid Leitner, Jeffrey Drazen, Diva J. Amon, Amanda F. Ziegler, Eric Anderson; dateIdentified: 2014; identificationRemarks: Identified by morphology of collected specimen; **Event:** samplingProtocol: Baited Trap; eventDate: 2013-10-20; eventTime: 7:44; habitat: Abyssal polymetallic-nodule field; fieldNumber: TR06; **Record Level:** language: en; institutionCode: UHM; datasetName: ABYSSLINE; basisOfRecord: HumanObservation
- **Type status:** Other material. **Occurrence:** recordedBy: Jeffrey Drazen, Astrid Leitner; individualCount: 1; lifeStage: Adult; behavior: On seafloor; occurrenceStatus: present; preparations: Imaged only; associatedReferences: Amon DJ, Ziegler AF, Dahlgren TG, Glover AG, Goineau A, Gooday AJ, Wiklund H, Smith CR. Insights into the abundance and diversity of abyssal megafauna in a polymetallic-nodule region in the eastern Clarion-Clipperton Zone. Scientific Reports. 2016;6. doi: 10.1038/srep30492; Leitner A, Neuheimer A, Donlon E, Smith CR, Drazen JC. Environmental and bathymetric influences on abyssal bait-attending communities of the Clarion Clipperton Zone. Deep Sea Research Part I: Oceanographic Research Papers. 2017. doi: 10.1016/j.dsr.2017.04.017; **Taxon:** taxonConceptID: Pachycara cf. nazca; scientificName: Pachycara nazca; kingdom: Animalia; phylum: Chordata; class: Actinopterygii; order: Perciformes; family: Zoarcidae; genus: Pachycara; taxonRank: species; scientificNameAuthorship: Anderson & Bluhm, 1997; **Location:** waterBody: Pacific Ocean; stateProvince: Clarion-Clipperton Zone; locality: UK Seabed Resources Ltd exploration contract area (UK-1); verbatimLocality: UK-1 Stratum B; maximumDepthInMeters: 4263; locationRemarks: RV Thompson Cruise TN319; decimalLatitude: 12.4526; decimalLongitude: -116.6488; geodeticDatum: WGS84; coordinateUncertaintyInMeters: 50; **Identification:** identifiedBy: Astrid Leitner, Jeffrey Drazen, Diva J. Amon, Amanda F. Ziegler; dateIdentified: 2015; identificationRemarks: Identified only from imagery; identificationQualifier: cf.; **Event:** samplingProtocol: Baited Camera; eventDate: 2015-03-07; eventTime: 17:06; habitat: Abyssal polymetallic-nodule field; fieldNumber: CA08; **Record Level:** language: en; institutionCode: UHM; datasetName: ABYSSLINE; basisOfRecord: HumanObservation

##### Notes

Large, deep-bodied eel-like shape. Large prominent lips and small rounded eyes. Large, rounded bulbous head. Smooth, scale-less appearance. Very round, large pectoral fins. Colors range from grey to white. Distinguished from other zoarcid morphospecies by large size, rounded head and pectoral fins.

Fig. 47

### Ctenophores of the UKSRL exploration contract area (UK-1) and the eastern Clarion-Clipperton Zone

#### Ctenophora

Eschscholtz, 1829

#### Tentaculata

Eschscholtz, 1825

#### Cydippida

Gegenbaur, 1856

#### Mertensiidae

L. Agassiz, 1860

#### cf. Mertensiidae morphospecies

##### Materials

- **Type status:** Other material. **Occurrence:** recordedBy: Diva J. Amon, Amanda F. Ziegler; individualCount: 1; lifeStage: Adult; behavior: Swimming; occurrenceStatus: present; preparations: Imaged only; **Taxon:** taxonConceptID: cf. Mertensiidae morphospecies; scientificName: Mertensiidae sp.; kingdom: Animalia; phylum: Ctenophora; class: Tentaculata; order: Cydippida; family: Mertensiidae; taxonRank: family; scientificNameAuthorship: L. Agassiz, 1860; **Location:** waterBody: Pacific Ocean; stateProvince: Clarion-Clipperton Zone; locality: UK Seabed Resources Ltd exploration contract area (UK-1); verbatimLocality: UK-1 Stratum B; maximumDepthInMeters: 4244; locationRemarks: RV Thompson Cruise TN319; decimalLatitude: 12.49983; decimalLongitude: -116.640175; geodeticDatum: WGS84; coordinateUncertaintyInMeters: 25; **Identification:** identifiedBy: Dhugal Lindsay, Diva J. Amon, Amanda F. Ziegler; dateIdentified: 2015; identificationRemarks: Identified only from imagery; identificationQualifier: cf.; **Event:** samplingProtocol: Autonomous Underwater Vehicle; eventDate: 2015-03-18; eventTime: 9:35; habitat: Abyssal polymetallic-nodule field; fieldNumber: Dive 9 (AV09); **Record Level:** language: en; institutionCode: UHM; datasetName: ABYSSLINE; basisOfRecord: HumanObservation

##### Notes

Two long tentacles with filamentous side branches. Elongate body (length >twice width) tinged red throughout with gut darker. Comb rows extending over almost whole body length. Aboral end from which tentacles protude with sunken statocyst.

Fig. 48

#### Lobata

Eschscholtz, 1825

#### cf. Lobata morphospecies 1

cf. Lobata morphospecies 1 In the “Atlas of Abyssal Megafauna Morphotypes of the Clarion-Clipperton Fracture Zone” created for the ISA (http://ccfzatlas.com/), this morphospecies is listed as "Unknown Ctenophora morphotype 1".

##### Materials

- **Type status:** Other material. **Occurrence:** recordedBy: Diva J. Amon, Amanda F. Ziegler; individualCount: 1; lifeStage: Adult; behavior: Swimming; occurrenceStatus: present; preparations: Imaged only; **Taxon:** taxonConceptID: cf. Lobata morphospecies 1; scientificName: Lobata sp.; kingdom: Animalia; phylum: Ctenophora; class: Tentaculata; order: Lobata; taxonRank: order; scientificNameAuthorship: Eschscholtz, 1825; **Location:** waterBody: Pacific Ocean; stateProvince: Clarion-Clipperton Zone; locality: UK Seabed Resources Ltd exploration contract area (UK-1); verbatimLocality: UK-1 Stratum B; maximumDepthInMeters: 4235; locationRemarks: RV Thompson Cruise TN319; decimalLatitude: 12.496153; decimalLongitude: -116.636375; geodeticDatum: WGS84; coordinateUncertaintyInMeters: 25; **Identification:** identifiedBy: Dhugal Lindsay, Diva J. Amon, Amanda F. Ziegler; dateIdentified: 2015; identificationRemarks: Identified only from imagery; identificationQualifier: cf.; **Event:** samplingProtocol: Autonomous Underwater Vehicle; eventDate: 2015-03-18; eventTime: 9:41; habitat: Abyssal polymetallic-nodule field; fieldNumber: Dive 9 (AV09); **Record Level:** language: en; institutionCode: UHM; datasetName: ABYSSLINE; basisOfRecord: HumanObservation

##### Notes

Whitish to transparent body with large, fleshy, rounded oral lobes but lacking auricles. Body flattened in tentacular plane. Stomodaeum not pigmented.

Fig. 49

#### cf. Lobata morphospecies 2

##### Materials

- **Type status:** Other material. **Occurrence:** recordedBy: Diva J. Amon, Amanda F. Ziegler; individualCount: 1; lifeStage: Adult; behavior: Swimming; occurrenceStatus: present; preparations: Imaged only; **Taxon:** taxonConceptID: cf. Lobata morphospecies 2; scientificName: Lobata sp.; kingdom: Animalia; phylum: Ctenophora; class: Tentaculata; order: Lobata; taxonRank: order; scientificNameAuthorship: Eschscholtz, 1825; **Location:** waterBody: Pacific Ocean; stateProvince: Clarion-Clipperton Zone; locality: UK Seabed Resources Ltd exploration contract area (UK-1); verbatimLocality: UK-1 Stratum B; maximumDepthInMeters: 4253; locationRemarks: RV Thompson Cruise TN319; decimalLatitude: 12.495027; decimalLongitude: -116.650252; geodeticDatum: WGS84; coordinateUncertaintyInMeters: 25; **Identification:** identifiedBy: Dhugal Lindsay, Diva J. Amon, Amanda F. Ziegler; dateIdentified: 2015; identificationRemarks: Identified only from imagery; identificationQualifier: cf.; **Event:** samplingProtocol: Autonomous Underwater Vehicle; eventDate: 2015-03-18; eventTime: 8:44; habitat: Abyssal polymetallic-nodule field; fieldNumber: Dive 9 (AV09); **Record Level:** language: en; institutionCode: UHM; datasetName: ABYSSLINE; basisOfRecord: HumanObservation

##### Notes

Transparent body with large, rounded oral lobes. Body small in comparison with lobes. Possible pigmentation in canal system.

Fig. 50

#### cf. Lobata morphospecies 3

##### Materials

- **Type status:** Other material. **Occurrence:** recordedBy: Diva J. Amon, Amanda F. Ziegler; individualCount: 1; lifeStage: Adult; behavior: Swimming; occurrenceStatus: present; preparations: Imaged only; **Taxon:** taxonConceptID: cf. Lobata morphospecies 3; scientificName: Lobata sp.; kingdom: Animalia; phylum: Ctenophora; class: Tentaculata; order: Lobata; taxonRank: order; scientificNameAuthorship: Eschscholtz, 1825; **Location:** waterBody: Pacific Ocean; stateProvince: Clarion-Clipperton Zone; locality: UK Seabed Resources Ltd exploration contract area (UK-1); verbatimLocality: UK-1 Stratum B; maximumDepthInMeters: 4225; locationRemarks: RV Thompson Cruise TN319; decimalLatitude: 12.579361; decimalLongitude: -116.687346; geodeticDatum: WGS84; coordinateUncertaintyInMeters: 25; **Identification:** identifiedBy: Dhugal Lindsay, Diva J. Amon, Amanda F. Ziegler; dateIdentified: 2015; identificationRemarks: Identified only from imagery; identificationQualifier: cf.; **Event:** samplingProtocol: Autonomous Underwater Vehicle; eventDate: 2015-03-09; eventTime: 3:27; habitat: Abyssal polymetallic-nodule field; fieldNumber: Dive 6 (AV06); **Record Level:** language: en; institutionCode: UHM; datasetName: ABYSSLINE; basisOfRecord: HumanObservation
- **Type status:** Other material. **Occurrence:** recordedBy: Diva J. Amon, Amanda F. Ziegler; individualCount: 1; lifeStage: Adult; behavior: Swimming; occurrenceStatus: present; preparations: Imaged only; **Taxon:** taxonConceptID: cf. Lobata morphospecies 3; scientificName: Lobata sp.; kingdom: Animalia; phylum: Ctenophora; class: Tentaculata; order: Lobata; taxonRank: order; scientificNameAuthorship: Eschscholtz, 1825; **Location:** waterBody: Pacific Ocean; stateProvince: Clarion-Clipperton Zone; locality: UK Seabed Resources Ltd exploration contract area (UK-1); verbatimLocality: UK-1 Stratum B; maximumDepthInMeters: 4224; locationRemarks: RV Thompson Cruise TN319; decimalLatitude: 12.579723; decimalLongitude: -116.727023; geodeticDatum: WGS84; coordinateUncertaintyInMeters: 25; **Identification:** identifiedBy: Dhugal Lindsay, Diva J. Amon, Amanda F. Ziegler; dateIdentified: 2015; identificationRemarks: Identified only from imagery; identificationQualifier: cf.; **Event:** samplingProtocol: Autonomous Underwater Vehicle; eventDate: 2015-03-09; eventTime: 7:56; habitat: Abyssal polymetallic-nodule field; fieldNumber: Dive 6 (AV06); **Record Level:** language: en; institutionCode: UHM; datasetName: ABYSSLINE; basisOfRecord: HumanObservation
- **Type status:** Other material. **Occurrence:** recordedBy: Diva J. Amon, Amanda F. Ziegler; individualCount: 1; lifeStage: Adult; behavior: Swimming; occurrenceStatus: present; preparations: Imaged only; **Taxon:** taxonConceptID: cf. Lobata morphospecies 3; scientificName: Lobata sp.; kingdom: Animalia; phylum: Ctenophora; class: Tentaculata; order: Lobata; taxonRank: order; scientificNameAuthorship: Eschscholtz, 1825; **Location:** waterBody: Pacific Ocean; stateProvince: Clarion-Clipperton Zone; locality: UK Seabed Resources Ltd exploration contract area (UK-1); verbatimLocality: UK-1 Stratum B; maximumDepthInMeters: 4221; locationRemarks: RV Thompson Cruise TN319; decimalLatitude: 12.581477; decimalLongitude: -116.722308; geodeticDatum: WGS84; coordinateUncertaintyInMeters: 25; **Identification:** identifiedBy: Dhugal Lindsay, Diva J. Amon, Amanda F. Ziegler; dateIdentified: 2015; identificationRemarks: Identified only from imagery; identificationQualifier: cf.; **Event:** samplingProtocol: Autonomous Underwater Vehicle; eventDate: 2015-03-09; eventTime: 10:13; habitat: Abyssal polymetallic-nodule field; fieldNumber: Dive 6 (AV06); **Record Level:** language: en; institutionCode: UHM; datasetName: ABYSSLINE; basisOfRecord: HumanObservation
- **Type status:** Other material. **Occurrence:** recordedBy: Diva J. Amon, Amanda F. Ziegler; individualCount: 1; lifeStage: Adult; behavior: Swimming; occurrenceStatus: present; preparations: Imaged only; **Taxon:** taxonConceptID: cf. Lobata morphospecies 3; scientificName: Lobata sp.; kingdom: Animalia; phylum: Ctenophora; class: Tentaculata; order: Lobata; taxonRank: order; scientificNameAuthorship: Eschscholtz, 1825; **Location:** waterBody: Pacific Ocean; stateProvince: Clarion-Clipperton Zone; locality: UK Seabed Resources Ltd exploration contract area (UK-1); verbatimLocality: UK-1 Stratum B; maximumDepthInMeters: 4226; locationRemarks: RV Thompson Cruise TN319; decimalLatitude: 12.579336; decimalLongitude: -116.723512; geodeticDatum: WGS84; coordinateUncertaintyInMeters: 25; **Identification:** identifiedBy: Dhugal Lindsay, Diva J. Amon, Amanda F. Ziegler; dateIdentified: 2015; identificationRemarks: Identified only from imagery; identificationQualifier: cf.; **Event:** samplingProtocol: Autonomous Underwater Vehicle; eventDate: 2015-03-09; eventTime: 11:03; habitat: Abyssal polymetallic-nodule field; fieldNumber: Dive 6 (AV06); **Record Level:** language: en; institutionCode: UHM; datasetName: ABYSSLINE; basisOfRecord: HumanObservation

##### Notes

Smoky pigmented body with very large, rounded oral lobes. Body width less than one-fourth of lobe width. Benthopelagic with lobes facing upwards.

Fig. 51

#### Lampoctenidae

Harbison, Matsumoto & Robison, 2001

#### Lampocteis

Harbison, Matsumoto & Robison, 2001

#### Lampocteis cf. cruentiventer

Harbison, Matsumoto & Robison, 2001

##### Materials

- **Type status:** Other material. **Occurrence:** recordedBy: Diva J. Amon, Amanda F. Ziegler; individualCount: 1; lifeStage: Adult; behavior: Swimming; occurrenceStatus: present; preparations: Imaged only; **Taxon:** taxonConceptID: Lampocteis cf. cruentiventer; scientificName: Lampocteis cruentiventer; kingdom: Animalia; phylum: Ctenophora; class: Tentaculata; order: Lobata; family: Lampoctenidae; genus: Lampocteis; taxonRank: species; scientificNameAuthorship: Harbison, Matsumoto & Robison, 2001; **Location:** waterBody: Pacific Ocean; stateProvince: Clarion-Clipperton Zone; locality: UK Seabed Resources Ltd exploration contract area (UK-1); verbatimLocality: UK-1 Stratum B; maximumDepthInMeters: 4210; locationRemarks: RV Thompson Cruise TN319; decimalLatitude: 12.496693; decimalLongitude: -116.629775; geodeticDatum: WGS84; coordinateUncertaintyInMeters: 25; **Identification:** identifiedBy: Dhugal Lindsay, Diva J. Amon, Amanda F. Ziegler; dateIdentified: 2015; identificationRemarks: Identified only from imagery; identificationQualifier: cf.; **Event:** samplingProtocol: Autonomous Underwater Vehicle; eventDate: 2015-03-18; eventTime: 9:57; habitat: Abyssal polymetallic-nodule field; fieldNumber: Dive 9 (AV09); **Record Level:** language: en; institutionCode: UHM; datasetName: ABYSSLINE; basisOfRecord: HumanObservation

##### Notes

Dark red pigmented body flattened in tentacular plane. With no long filamentous tentacles. Long comb rows.

Fig. 52

### Molluscs of the UKSRL exploration contract area (UK-1) and the eastern Clarion-Clipperton Zone

#### Mollusca

Linnaeus, 1758

#### Bivalvia

Linnaeus, 1758

#### cf. Bivalvia morphospecies

##### Materials

- **Type status:** Other material. **Occurrence:** recordedBy: Diva J. Amon, Amanda F. Ziegler; individualCount: 1; lifeStage: Adult; behavior: On seafloor; occurrenceStatus: present; preparations: Imaged only; associatedReferences: Amon DJ, Ziegler AF, Dahlgren TG, Glover AG, Goineau A, Gooday AJ, Wiklund H, Smith CR. Insights into the abundance and diversity of abyssal megafauna in a polymetallic-nodule region in the eastern Clarion-Clipperton Zone. Scientific Reports. 2016;6. doi: 10.1038/srep30492; **Taxon:** taxonConceptID: cf. Bivalvia morphospecies; scientificName: Bivalvia sp.; kingdom: Animalia; phylum: Mollusca; class: Bivalvia; taxonRank: class; scientificNameAuthorship: Linnaeus, 1758; **Location:** waterBody: Pacific Ocean; stateProvince: Clarion-Clipperton Zone; locality: Eastern Clarion-Clipperton Zone; verbatimLocality: Site EPIRB; maximumDepthInMeters: 3952; locationRemarks: RV Melville Cruise MV1313; decimalLatitude: 13.6794; decimalLongitude: -114.4147; geodeticDatum: WGS84; coordinateUncertaintyInMeters: 25; **Identification:** identifiedBy: Janet Voight, Diva J. Amon, Amanda F. Ziegler; dateIdentified: 2014; identificationRemarks: Identified only from imagery; identificationQualifier: cf.; **Event:** samplingProtocol: Remotely Operated Vehicle; eventDate: 2013-10-23; eventTime: 9:44; habitat: Abyssal polymetallic-nodule field; fieldNumber: Dive 7 (RV07); **Record Level:** language: en; institutionCode: UHM; datasetName: ABYSSLINE; basisOfRecord: HumanObservation
- **Type status:** Other material. **Occurrence:** recordedBy: Diva J. Amon, Amanda F. Ziegler; individualCount: 1; lifeStage: Adult; behavior: On seafloor; occurrenceStatus: present; preparations: Imaged only; associatedReferences: Amon DJ, Ziegler AF, Dahlgren TG, Glover AG, Goineau A, Gooday AJ, Wiklund H, Smith CR. Insights into the abundance and diversity of abyssal megafauna in a polymetallic-nodule region in the eastern Clarion-Clipperton Zone. Scientific Reports. 2016;6. doi: 10.1038/srep30492; **Taxon:** taxonConceptID: cf. Bivalvia morphospecies; scientificName: Bivalvia sp.; kingdom: Animalia; phylum: Mollusca; class: Bivalvia; taxonRank: class; scientificNameAuthorship: Linnaeus, 1758; **Location:** waterBody: Pacific Ocean; stateProvince: Clarion-Clipperton Zone; locality: Eastern Clarion-Clipperton Zone; verbatimLocality: Site EPIRB; maximumDepthInMeters: 3927; locationRemarks: RV Melville Cruise MV1313; decimalLatitude: 13.6789; decimalLongitude: -114.4093; geodeticDatum: WGS84; coordinateUncertaintyInMeters: 25; **Identification:** identifiedBy: Janet Voight, Diva J. Amon, Amanda F. Ziegler; dateIdentified: 2014; identificationRemarks: Identified only from imagery; identificationQualifier: cf.; **Event:** samplingProtocol: Remotely Operated Vehicle; eventDate: 2013-10-23; eventTime: 10:47; habitat: Abyssal polymetallic-nodule field; fieldNumber: Dive 7 (RV07); **Record Level:** language: en; institutionCode: UHM; datasetName: ABYSSLINE; basisOfRecord: HumanObservation
- **Type status:** Other material. **Occurrence:** recordedBy: Diva J. Amon, Amanda F. Ziegler; individualCount: 1; lifeStage: Adult; behavior: On seafloor; occurrenceStatus: present; preparations: Imaged only; associatedReferences: Amon DJ, Ziegler AF, Dahlgren TG, Glover AG, Goineau A, Gooday AJ, Wiklund H, Smith CR. Insights into the abundance and diversity of abyssal megafauna in a polymetallic-nodule region in the eastern Clarion-Clipperton Zone. Scientific Reports. 2016;6. doi: 10.1038/srep30492; **Taxon:** taxonConceptID: cf. Bivalvia morphospecies; scientificName: Bivalvia sp.; kingdom: Animalia; phylum: Mollusca; class: Bivalvia; taxonRank: class; scientificNameAuthorship: Linnaeus, 1758; **Location:** waterBody: Pacific Ocean; stateProvince: Clarion-Clipperton Zone; locality: Eastern Clarion-Clipperton Zone; verbatimLocality: Site EPIRB; maximumDepthInMeters: 3945; locationRemarks: RV Melville Cruise MV1313; decimalLatitude: 13.6794; decimalLongitude: -114.4130; geodeticDatum: WGS84; coordinateUncertaintyInMeters: 25; **Identification:** identifiedBy: Janet Voight, Diva J. Amon, Amanda F. Ziegler; dateIdentified: 2014; identificationRemarks: Identified only from imagery; identificationQualifier: cf.; **Event:** samplingProtocol: Remotely Operated Vehicle; eventDate: 2013-10-23; eventTime: 10:06; habitat: Abyssal polymetallic-nodule field; fieldNumber: Dive 7 (RV07); **Record Level:** language: en; institutionCode: UHM; datasetName: ABYSSLINE; basisOfRecord: HumanObservation

##### Notes

Infaunal, two-valves that meet at line; view in Fig. 53 a, c virtually into expanded siphon which is extended; Fig. 53b shows closed valves. Valve length enigmatically large (ca. 3-4 cm) for these depths, essentially 50% larger than expected.

Fig. 53

#### Cephalopoda

Cuvier, 1795

#### Octopoda

Leach, 1818

#### Cirroteuthidae

Keferstein, 1866

#### cf. Cirroteuthidae morphospecies

cf. Cirroteuthidae morphospecies In the “Atlas of Abyssal Megafauna Morphotypes of the Clarion-Clipperton Fracture Zone” created for the ISA (http://ccfzatlas.com/), this morphospecies is listed as "*Cirroteuthis* morphotype".

##### Materials

- **Type status:** Other material. **Occurrence:** recordedBy: Diva J. Amon, Amanda F. Ziegler; individualCount: 1; behavior: Swimming; occurrenceStatus: present; preparations: Imaged only; **Taxon:** taxonConceptID: cf. Cirroteuthidae morphospecies; scientificName: Cirroteuthidae sp.; kingdom: Animalia; phylum: Mollusca; class: Cephalopoda; order: Octopoda; family: Cirroteuthidae; taxonRank: family; scientificNameAuthorship: Keferstein, 1866; **Location:** waterBody: Pacific Ocean; stateProvince: Clarion-Clipperton Zone; locality: UK Seabed Resources Ltd exploration contract area (UK-1); verbatimLocality: UK-1 Stratum B; maximumDepthInMeters: 4225; locationRemarks: RV Thompson Cruise TN319; decimalLatitude: 12.5819; decimalLongitude: -116.7209; geodeticDatum: WGS84; coordinateUncertaintyInMeters: 25; **Identification:** identifiedBy: Janet Voight, Diva J. Amon, Amanda F. Ziegler; dateIdentified: 2015; identificationReferences: http://tolweb.org/Cirrata/20086; identificationRemarks: Identified only from imagery; identificationQualifier: cf.; **Event:** samplingProtocol: Autonomous Underwater Vehicle; eventDate: 2015-03-09; eventTime: 11:07; habitat: Abyssal polymetallic-nodule field; fieldNumber: Dive 6 (AV06); **Record Level:** language: en; institutionCode: UHM; datasetName: ABYSSLINE; basisOfRecord: HumanObservation
- **Type status:** Other material. **Occurrence:** recordedBy: Diva J. Amon, Amanda F. Ziegler; individualCount: 1; behavior: Swimming; occurrenceStatus: present; preparations: Imaged only; **Taxon:** taxonConceptID: cf. Cirroteuthidae morphospecies; scientificName: Cirroteuthidae sp.; kingdom: Animalia; phylum: Mollusca; class: Cephalopoda; order: Octopoda; family: Cirroteuthidae; taxonRank: family; scientificNameAuthorship: Keferstein, 1866; **Location:** waterBody: Pacific Ocean; stateProvince: Clarion-Clipperton Zone; locality: UK Seabed Resources Ltd exploration contract area (UK-1); verbatimLocality: UK-1 Stratum B; maximumDepthInMeters: 4233; locationRemarks: RV Thompson Cruise TN319; decimalLatitude: 12.5341; decimalLongitude: -116.6039; geodeticDatum: WGS84; coordinateUncertaintyInMeters: 25; **Identification:** identifiedBy: Janet Voight, Diva J. Amon, Amanda F. Ziegler; dateIdentified: 2015; identificationReferences: http://tolweb.org/Cirrata/20086; identificationRemarks: Identified only from imagery; identificationQualifier: cf.; **Event:** samplingProtocol: Autonomous Underwater Vehicle; eventDate: 2015-03-18; eventTime: 16:00; habitat: Abyssal polymetallic-nodule field; fieldNumber: Dive 9 (AV09); **Record Level:** language: en; institutionCode: UHM; datasetName: ABYSSLINE; basisOfRecord: HumanObservation
- **Type status:** Other material. **Occurrence:** recordedBy: Diva J. Amon, Amanda F. Ziegler; individualCount: 1; behavior: Swimming; occurrenceStatus: present; preparations: Imaged only; **Taxon:** taxonConceptID: cf. Cirroteuthidae morphospecies; scientificName: Cirroteuthidae sp.; kingdom: Animalia; phylum: Mollusca; class: Cephalopoda; order: Octopoda; family: Cirroteuthidae; taxonRank: family; scientificNameAuthorship: Keferstein, 1866; **Location:** waterBody: Pacific Ocean; stateProvince: Clarion-Clipperton Zone; locality: UK Seabed Resources Ltd exploration contract area (UK-1); verbatimLocality: UK-1 Stratum B; maximumDepthInMeters: 4254; locationRemarks: RV Thompson Cruise TN319; decimalLatitude: 12.4932; decimalLongitude: -116.6517; geodeticDatum: WGS84; coordinateUncertaintyInMeters: 25; **Identification:** identifiedBy: Janet Voight, Diva J. Amon, Amanda F. Ziegler; dateIdentified: 2015; identificationReferences: http://tolweb.org/Cirrata/20086; identificationRemarks: Identified only from imagery; identificationQualifier: cf.; **Event:** samplingProtocol: Autonomous Underwater Vehicle; eventDate: 2015-03-04; eventTime: 0:31; habitat: Abyssal polymetallic-nodule field; fieldNumber: Dive 5 (AV05); **Record Level:** language: en; institutionCode: UHM; datasetName: ABYSSLINE; basisOfRecord: HumanObservation
- **Type status:** Other material. **Occurrence:** recordedBy: Diva J. Amon, Amanda F. Ziegler; individualCount: 1; behavior: Swimming; occurrenceStatus: present; preparations: Imaged only; **Taxon:** taxonConceptID: cf. Cirroteuthidae morphospecies; scientificName: Cirroteuthidae sp.; kingdom: Animalia; phylum: Mollusca; class: Cephalopoda; order: Octopoda; family: Cirroteuthidae; taxonRank: family; scientificNameAuthorship: Keferstein, 1866; **Location:** waterBody: Pacific Ocean; stateProvince: Clarion-Clipperton Zone; locality: UK Seabed Resources Ltd exploration contract area (UK-1); verbatimLocality: UK-1 Stratum B; maximumDepthInMeters: 4223; locationRemarks: RV Thompson Cruise TN319; decimalLatitude: 12.5854; decimalLongitude: -116.7193; geodeticDatum: WGS84; coordinateUncertaintyInMeters: 25; **Identification:** identifiedBy: Janet Voight, Diva J. Amon, Amanda F. Ziegler; dateIdentified: 2015; identificationReferences: http://tolweb.org/Cirrata/20086; identificationRemarks: Identified only from imagery; identificationQualifier: cf.; **Event:** samplingProtocol: Autonomous Underwater Vehicle; eventDate: 2015-03-09; eventTime: 9:40; habitat: Abyssal polymetallic-nodule field; fieldNumber: Dive 6 (AV06); **Record Level:** language: en; institutionCode: UHM; datasetName: ABYSSLINE; basisOfRecord: HumanObservation

##### Notes

Octopod with large paired fins emerging from lateral mantle, secondary web between the arms and primary web, and two rows of long cirri lateral to single row of arm suckers. Head about the same width as mantle. Mantle notably elongate, vague translucence. Distal arms often coiled aborally, exposing the secondary web.

Fig. 54

#### Opisthoteuthidae

Verrill, 1896

#### Grimpoteuthis

Robson, 1932

#### cf. Grimpoteuthis morphospecies

cf. Grimpoteuthis morphospecies In the “Atlas of Abyssal Megafauna Morphotypes of the Clarion-Clipperton Fracture Zone” created for the ISA (http://ccfzatlas.com/), this morphospecies is listed as "*Grimpoteuthis* morphotype".

##### Materials

- **Type status:** Other material. **Occurrence:** recordedBy: Diva J. Amon, Amanda F. Ziegler; individualCount: 1; behavior: Swimming; occurrenceStatus: present; preparations: Imaged only; **Taxon:** taxonConceptID: cf. *Grimpoteuthis* morphospecies; scientificName: *Grimpoteuthis* sp.; kingdom: Animalia; phylum: Mollusca; class: Cephalopoda; order: Octopoda; family: Opisthoteuthidae ; genus: Grimpoteuthis; taxonRank: genus; scientificNameAuthorship: Robson, 1932; **Location:** waterBody: Pacific Ocean; stateProvince: Clarion-Clipperton Zone; locality: UK Seabed Resources Ltd exploration contract area (UK-1); verbatimLocality: UK-1 Stratum B; maximumDepthInMeters: 4249; locationRemarks: RV Thompson Cruise TN319; decimalLatitude: 12.4983; decimalLongitude: -116.6453; geodeticDatum: WGS84; coordinateUncertaintyInMeters: 25; **Identification:** identifiedBy: Janet Voight, Diva J. Amon, Amanda F. Ziegler; dateIdentified: 2015; identificationReferences: http://tolweb.org/Cirrata/20086; identificationRemarks: Identified only from imagery; identificationQualifier: cf.; **Event:** samplingProtocol: Autonomous Underwater Vehicle; eventDate: 2015-03-03; eventTime: 22:05; habitat: Abyssal polymetallic-nodule field; fieldNumber: Dive 5 (AV05); **Record Level:** language: en; institutionCode: UHM; datasetName: ABYSSLINE; basisOfRecord: HumanObservation
- **Type status:** Other material. **Occurrence:** recordedBy: Diva J. Amon, Amanda F. Ziegler; individualCount: 1; behavior: Swimming; occurrenceStatus: present; preparations: Imaged only; **Taxon:** taxonConceptID: cf. *Grimpoteuthis* morphospecies; scientificName: *Grimpoteuthis* sp.; kingdom: Animalia; phylum: Mollusca; class: Cephalopoda; order: Octopoda; family: Opisthoteuthidae ; genus: Grimpoteuthis; taxonRank: genus; scientificNameAuthorship: Robson, 1932; **Location:** waterBody: Pacific Ocean; stateProvince: Clarion-Clipperton Zone; locality: UK Seabed Resources Ltd exploration contract area (UK-1); verbatimLocality: UK-1 Stratum B; maximumDepthInMeters: 4253; locationRemarks: RV Thompson Cruise TN319; decimalLatitude: 12.5012; decimalLongitude: -116.6500; geodeticDatum: WGS84; coordinateUncertaintyInMeters: 25; **Identification:** identifiedBy: Janet Voight, Diva J. Amon, Amanda F. Ziegler; dateIdentified: 2015; identificationReferences: http://tolweb.org/Cirrata/20086; identificationRemarks: Identified only from imagery; identificationQualifier: cf.; **Event:** samplingProtocol: Autonomous Underwater Vehicle; eventDate: 2015-03-04; eventTime: 3:56; habitat: Abyssal polymetallic-nodule field; fieldNumber: Dive 5 (AV05); **Record Level:** language: en; institutionCode: UHM; datasetName: ABYSSLINE; basisOfRecord: HumanObservation
- **Type status:** Other material. **Occurrence:** recordedBy: Diva J. Amon, Amanda F. Ziegler; individualCount: 1; behavior: On seafloor; occurrenceStatus: present; preparations: Imaged only; **Taxon:** taxonConceptID: cf. *Grimpoteuthis* morphospecies; scientificName: *Grimpoteuthis* sp.; kingdom: Animalia; phylum: Mollusca; class: Cephalopoda; order: Octopoda; family: Opisthoteuthidae ; genus: Grimpoteuthis; taxonRank: genus; scientificNameAuthorship: Robson, 1932; **Location:** waterBody: Pacific Ocean; stateProvince: Clarion-Clipperton Zone; locality: UK Seabed Resources Ltd exploration contract area (UK-1); verbatimLocality: UK-1 Stratum B; maximumDepthInMeters: 4209; locationRemarks: RV Thompson Cruise TN319; decimalLatitude: 12.5720; decimalLongitude: -116.7349; geodeticDatum: WGS84; coordinateUncertaintyInMeters: 25; **Identification:** identifiedBy: Janet Voight, Diva J. Amon, Amanda F. Ziegler; dateIdentified: 2015; identificationReferences: http://tolweb.org/Cirrata/20086; identificationRemarks: Identified only from imagery; identificationQualifier: cf.; **Event:** samplingProtocol: Autonomous Underwater Vehicle; eventDate: 2015-03-09; eventTime: 7:42; habitat: Abyssal polymetallic-nodule field; fieldNumber: Dive 6 (AV06); **Record Level:** language: en; institutionCode: UHM; datasetName: ABYSSLINE; basisOfRecord: HumanObservation
- **Type status:** Other material. **Occurrence:** recordedBy: Diva J. Amon, Amanda F. Ziegler; individualCount: 1; behavior: On seafloor; occurrenceStatus: present; preparations: Imaged only; **Taxon:** taxonConceptID: cf. *Grimpoteuthis* morphospecies; scientificName: *Grimpoteuthis* sp.; kingdom: Animalia; phylum: Mollusca; class: Cephalopoda; order: Octopoda; family: Opisthoteuthidae ; genus: Grimpoteuthis; taxonRank: genus; scientificNameAuthorship: Robson, 1932; **Location:** waterBody: Pacific Ocean; stateProvince: Clarion-Clipperton Zone; locality: UK Seabed Resources Ltd exploration contract area (UK-1); verbatimLocality: UK-1 Stratum B; maximumDepthInMeters: 4224; locationRemarks: RV Thompson Cruise TN319; decimalLatitude: 12.5879; decimalLongitude: -116.7167; geodeticDatum: WGS84; coordinateUncertaintyInMeters: 25; **Identification:** identifiedBy: Janet Voight, Diva J. Amon, Amanda F. Ziegler; dateIdentified: 2015; identificationReferences: http://tolweb.org/Cirrata/20086; identificationRemarks: Identified only from imagery; identificationQualifier: cf.; **Event:** samplingProtocol: Autonomous Underwater Vehicle; eventDate: 2015-03-09; eventTime: 9:44; habitat: Abyssal polymetallic-nodule field; fieldNumber: Dive 6 (AV06); **Record Level:** language: en; institutionCode: UHM; datasetName: ABYSSLINE; basisOfRecord: HumanObservation

##### Notes

Octopod with moderate-sized paired fins emerging from lateral mantle, simple interbrachial web, often two-toned with light mantle, darker arms/web, and two rows of cirri lateral to arm suckers. Arms often held nearly straight. Often seen near or on the seafloor.

Fig. 55

#### Enteroctopodidae

Strugnell, Norman, Vecchione, Guzik & Allcock, 2014

#### Muusoctopus

Gleadall, 2004

#### cf. Muusoctopus morphospecies

##### Materials

- **Type status:** Other material. **Occurrence:** recordedBy: Diva J. Amon, Amanda F. Ziegler; individualCount: 1; behavior: On seafloor; occurrenceStatus: present; preparations: Imaged only; **Taxon:** taxonConceptID: cf. *Muusoctopus* morphospecies; scientificName: *Muusoctopus* sp.; kingdom: Animalia; phylum: Mollusca; class: Cephalopoda; order: Octopoda; family: Enteroctopodidae; genus: Muusoctopus; taxonRank: genus; scientificNameAuthorship: Gleadall, 2004; **Location:** waterBody: Pacific Ocean; stateProvince: Clarion-Clipperton Zone; locality: UK Seabed Resources Ltd exploration contract area (UK-1); verbatimLocality: UK-1 Stratum B; maximumDepthInMeters: 4225; locationRemarks: RV Thompson Cruise TN319; decimalLatitude: 12.5788; decimalLongitude: -116.6873; geodeticDatum: WGS84; coordinateUncertaintyInMeters: 25; **Identification:** identifiedBy: Janet Voight, Diva J. Amon, Amanda F. Ziegler; dateIdentified: 2015; identificationReferences: Voight, Janet R. "Observations of deep-sea octopodid behavior from undersea vehicles." American Malacological Bulletin 24.1 (2008): 43-50.; identificationRemarks: Identified only from imagery; identificationQualifier: cf.; **Event:** samplingProtocol: Autonomous Underwater Vehicle; eventDate: 2015-03-09; eventTime: 3:50; habitat: Abyssal polymetallic-nodule field; fieldNumber: Dive 6 (AV06); **Record Level:** language: en; institutionCode: UHM; datasetName: ABYSSLINE; basisOfRecord: HumanObservation

##### Notes

Finless, benthic octopod with two rows of arm suckers, head narrower than mantle, arms coiled aborally when still, extended into sediment when walking. Swimming is propelled by mantle jets, not by fins. Ventrum often darker than dorsum, but not necessarily.

Fig. 56

## Discussion

Although many of the morphospecies included here remain taxonomically ambiguous, we provide the first image atlas of annelid, arthropod, bryozoan, chordate, ctenophore and mollusc morphospecies inhabiting the UK-1 exploration contract area and the eastern CCZ. At least 55 distinct morphospecies (8 Annelida, 12 Arthropoda, 4 Bryozoa, 22 Chordata, 5 Ctenophora, and 4 Mollusca) were observed, although this is likely an underestimate given the poor image quality and presence of cryptic species (Amon et al. 2016). When the echinoderm megafauna are included, at least 117 morphospecies from seven phyla are documented (Amon et al. 2017). This is the second-highest total species richness recorded in the CCZ region for these seven phyla. This is remarkable given that this total is from one exploration contract area (UK-1) and a single dive site east of the contract area, and currently excludes two of the most speciose phyla, Cnidaria and Porifera, which will be treated in separate atlases in the future (Amon et al. 2016, Amon et al. 2016, Foell and Pawson 1986, Leitner et al. 2017, Tilot 2006, Wang and Lu 2002). However, we recognise that the comparison of species richness for these seven phyla aross the region is only valid if sampling effort was similar or standardised (Amon et al. 2016).

The numbers of annelid morphospecies in this study are similar to those in the only other CCZ megafauna study that includes annelids (Tilot 2006), although it is not clear whether there is overlap between these morphospecies. However, the number of arthropod morphospecies in Tilot (2006) (> 20) is considerably more than observed during this study and others in the CCZ (Amon et al. 2016, Foell and Pawson 1986, Leitner et al. 2017, Wang and Lu 2002). Interestingly, the observations of *Hymenopenaeus nereus* in the UKSRL exploration area and at the EPIRB site represent a range extension for this species (Hendrickx and Wicksten 2016). Similarly, *Eurythenes magellanicus*, collected from the UKSRL exploration area and confirmed by molecular and morphological analyses, has been previously recorded only from the Drake Passage and Brazil Basin (d’Acoz and Havermens 2015), representing a significant range extension for this species.

Tilot (2006) recorded nearly 30 chordate morphospecies, the highest diversity in the CCZ thus far, though comparable to that recorded here. Leitner et al. (2017) observed eight bait-attending fish morphospecies from the first scavenging experiments in the CCZ. The quantitative study by Amon et al. (2016) which utilised many of the images from AB01 included in this study reported six fish morphospecies from UK-1 Stratum A and EPIRB only, which included both bait-attending and non-bait-attending. The total number of fish morphospecies from Amon et al. (2016) and Leitner et al. (2017) (11 morphospecies) equals the number in the CCFZ online atlas (Martinez-Arbizu et al. 2013), although morphospecies differ. The macrourid species *Coryphaenoides yaquinae* is known from the Pacific Ocean whereas *C. armatus* is thought to have a worldwide distribution (Jamieson et al. 2012), however Amon et al. (2016) and Leitner et al. (2017) were the first to report and confirm the presence of these species in the UKSRL exploration contract area. It should be noted that distinguishing between *C. armatus* and *C. yaquinae* in imagery is very difficult and so morphological and molecular analyses of specimens should be used to confirm identification. The presence of Pachycara cf. nazca in the UKSRL exploration contract area also respresents a range extension for this species as it is previously known from the abyssal southeast Pacific Ocean off Peru (Anderson and Bluhm 1996).

The rarer megafaunal phyla in the CCZ appear to be the Bryozoa, Ctenophora and Mollusca but this study has recorded the highest diversities thus far for each of these phyla (four, five and four morphospecies respectively). Only one other megafaunal CCZ study has included bryozoans, with one species recorded (Foell and Pawson 1986). Previous studies identified between one and two ctenophore morphospecies in the CCZ (Martinez-Arbizu et al. 2013, Tilot 2006), whereas as many as five mollusc morphospecies have been observed. Between two and four cephalopod morphospecies (Foell and Pawson 1986, Martinez-Arbizu et al. 2013, Tilot 2006, Vecchione 2016, Wang and Lu 2002) have been recorded from elsewhere in the CCZ, however this may underestimate the cephalopod biodiversity as these highly mobile megafauna are able to evade ROVs and other imaging platforms. Megafaunal bivalves have only been mentioned briefly previously, perhaps because, like bryozoans, they are not observed easily in deep-sea imagery due to their small size (Amon et al. 2016, Tilot 2006, Wang and Lu 2002).

These morphospecies represent a range of functional traits: the serpulid and sabellid polychaetes and bryozoans are sessile suspension feeders, reliant on the polymetallic nodules as hard substrate, whereas most of the arthropods, chordates, cephalopods and ctenophores, are predatory (Amon et al. 2016, Amon et al. 2016, Leitner et al. 2017, Martinez-Arbizu et al. 2013). This differs from the echinoderms recorded in the UKSRL area and at the EPIRB site as most of those morphospecies were deposit feeders of suspension feeders (Amon et al. 2017). Approximately one third of the morphospecies in this atlas have been observed in other contract areas in the CCZ (Amon et al. 2016, Amon et al. 2016, Foell and Pawson 1986, Leitner et al. 2017, Martinez-Arbizu et al. 2013, Tilot 2006, Vecchione 2016, Wang and Lu 2002), although this may be an overestimate given the presence of cryptic species and the problems identifying megafauna from imagery, as has been experienced during studies in other poorly-explored areas (Amon et al. 2016, Bickford et al. 2007, Linse et al. 2007, Vrijenhoek 2009). Information similar to that presented here will likely be crucial to informing the future environmental management of the region.

While this image atlas, as well as the echinoderm atlas (Amon et al. 2017), has expanded the knowledge of benthic fauna in the UK-1 exploration contract area and overall CCZ, there is still a need for further high-quality imagery of fauna, and especially physical megafaunal specimens to groundtruth the imaged morphospecies via detailed morphological and molecular analyses. We expect that a number of the morphospecies included in this atlas may be new to science, new records, or poorly known, but this can only be confirmed when specimens are collected and analysed. Molecular analyses are especially important given the presence of cryptic species. The limited collection of voucher specimens in the CCZ continues to severely hamper reliable estimation of species richness and species distributions. Although the taxonomic identification of preserved material is always necessary, we hope that this atlas will aid scientists by showing what these morphospecies look like in situ in their natural surroundings, as well as by providing some ecological information (e.g. feeding modes, preferred habitat etc.). This information will be important in estimating the human impact on this ecosystem. Furthermore, the appearance of morphospecies captured in situ in images can drastically differ from that of collected or preserved material, especially when relatively rudimentary collection equipment (trawls, dredges etc.) are used. As mentioned in Amon et al. (2016), those working in the CCZ must share detailed descriptions of their equipment and methods to facilitate data standardization and statistically-rigorous regional comparisons. It is also important that the ISA-sponsored online atlas continues to be updated with new imagery (such as the images in this atlas), and that the morphospecies are properly identified with the help of taxonomists.

## Supplementary Material

Supplementary material 1 Concise table of megafaunal morphospecies locations Data type: Occurences
File: oo_146487.xlsx DJ Amon

## Figures and Tables

**Figure 2.**
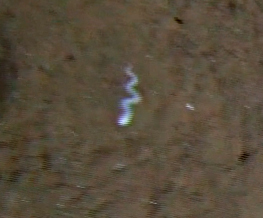
cf. Polychaeta morphospecies in situ on the seafloor in the UK-1 exploration contract area. Image corresponds with the data above. Image attribution: DJ Amon & CR Smith, University of Hawai’i.

**Figure 3.**
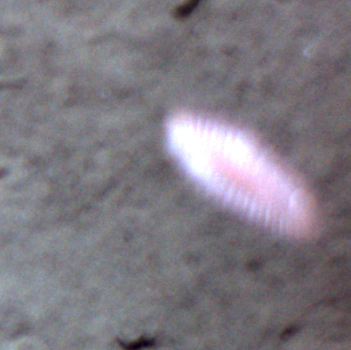
cf. Polynoidae morphospecies in situ swimming above the seafloor in the UK-1 exploration contract area. Image corresponds with the data above. Image attribution: Woods Hole Oceanographic Institution.

**Figure 4a.**
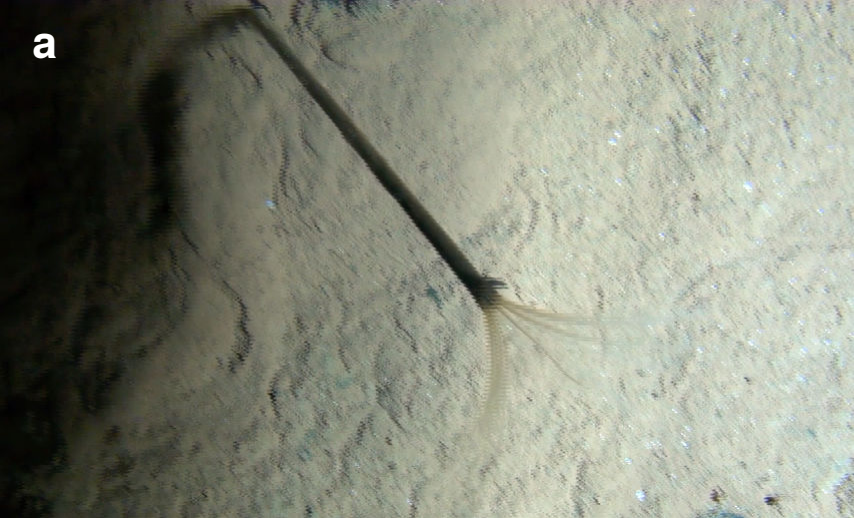
cf. Sabellidae morphospecies 1 in situ on the seafloor. Image attribution: DJ Amon & CR Smith, University of Hawai’i.

**Figure 4b.**
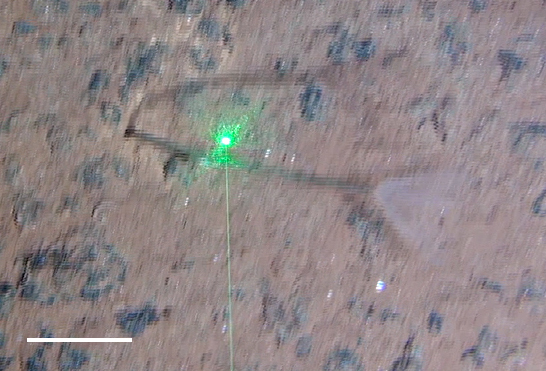
cf. Sabellidae morphospecies 1 in situ on the seafloor. Scale bar is 10 cm. Image attribution: DJ Amon & CR Smith, University of Hawai’i.

**Figure 5.**
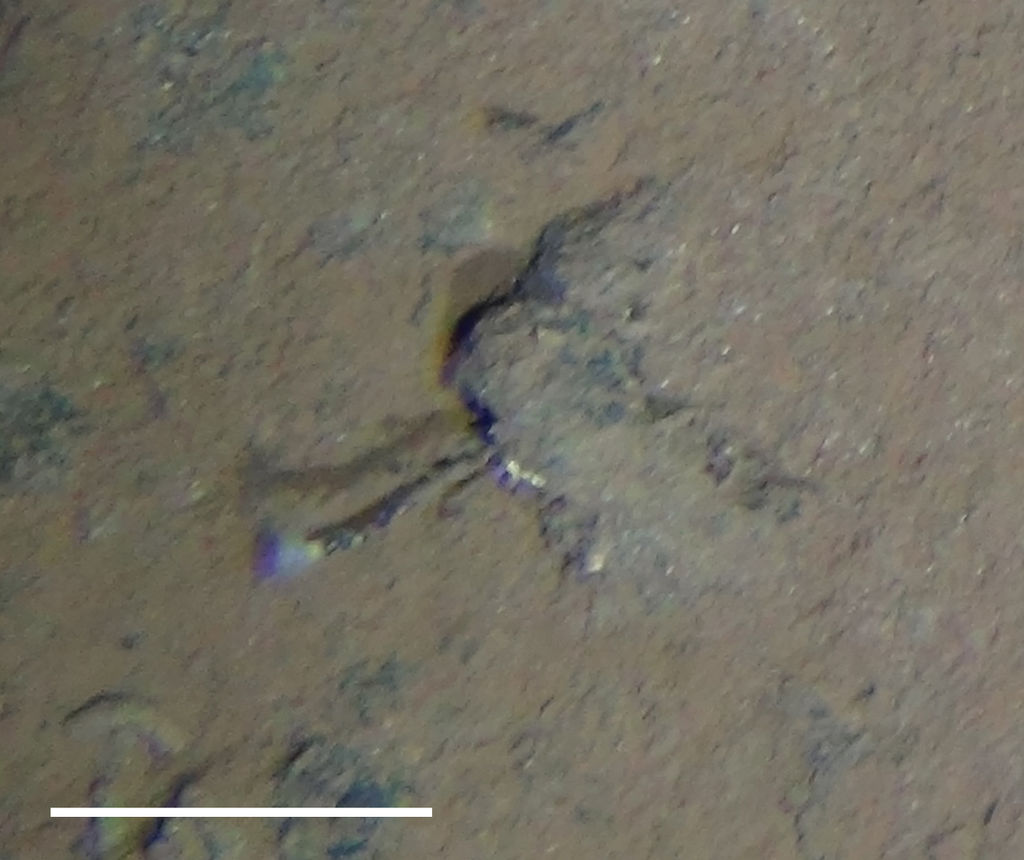
cf. Sabellidae morphospecies 2 attached to a polymetallic nodule on the seafloor in the eastern CCZ. Image corresponds with the data above. Scale bar is 10 cm. Image attribution: DJ Amon & CR Smith, University of Hawai’i.

**Figure 6.**
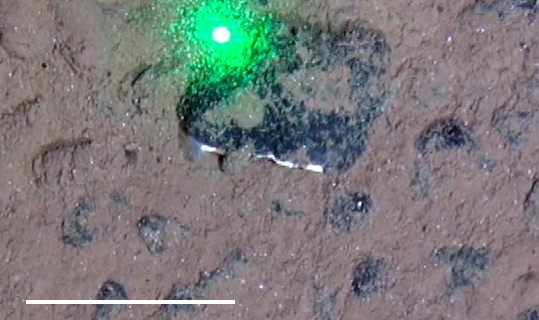
cf. Serpulidae morphospecies 1 attached to a polymetallic nodule on the seafloor in the UK-1 exploration contract area. Image corresponds with the data above. Scale bar is 10 cm. Image attribution: DJ Amon & CR Smith, University of Hawai’i.

**Figure 7.**
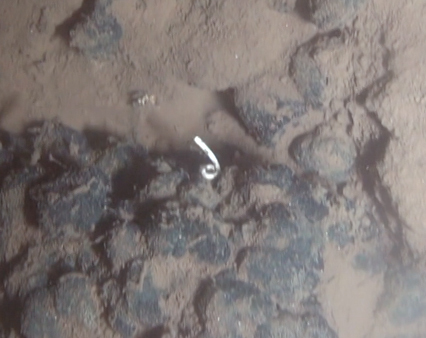
cf. Serpulidae morphospecies 2 attached to a polymetallic nodule on the seafloor in the UK-1 exploration contract area. Image corresponds with the data above. Image attribution: DJ Amon & CR Smith, University of Hawai’i.

**Figure 8.**
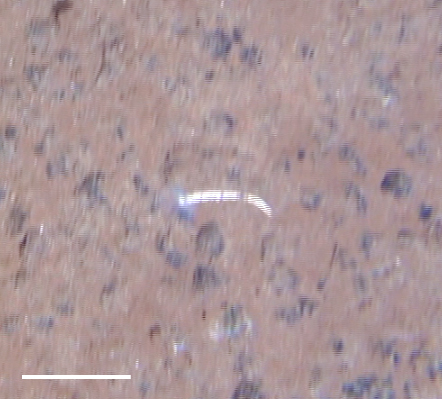
cf. Serpulidae morphospecies 3 in situ on the seafloor in the eastern CCZ. Image corresponds with the data above. Scale bar is 10 cm. Image attribution: DJ Amon & CR Smith, University of Hawai’i.

**Figure 9.**
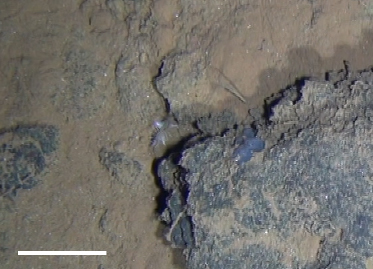
cf. Acrocirridae morphospecies in situ swimming above the seafloor in the eastern CCZ. Image corresponds with the data above. Scale bar is 10 cm. Image attribution: DJ Amon & CR Smith, University of Hawai’i.

**Figure 10.**
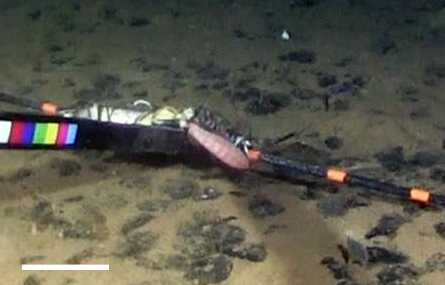
cf. *Eurythenes* morphospecies 1 in situ feeding on bait on the seafloor in the UK-1 exploration contract area. Image corresponds with the data above. Scale bar is 10 cm. Image attribution: A Leitner and J Drazen, University of Hawai’i.

**Figure 11a.**
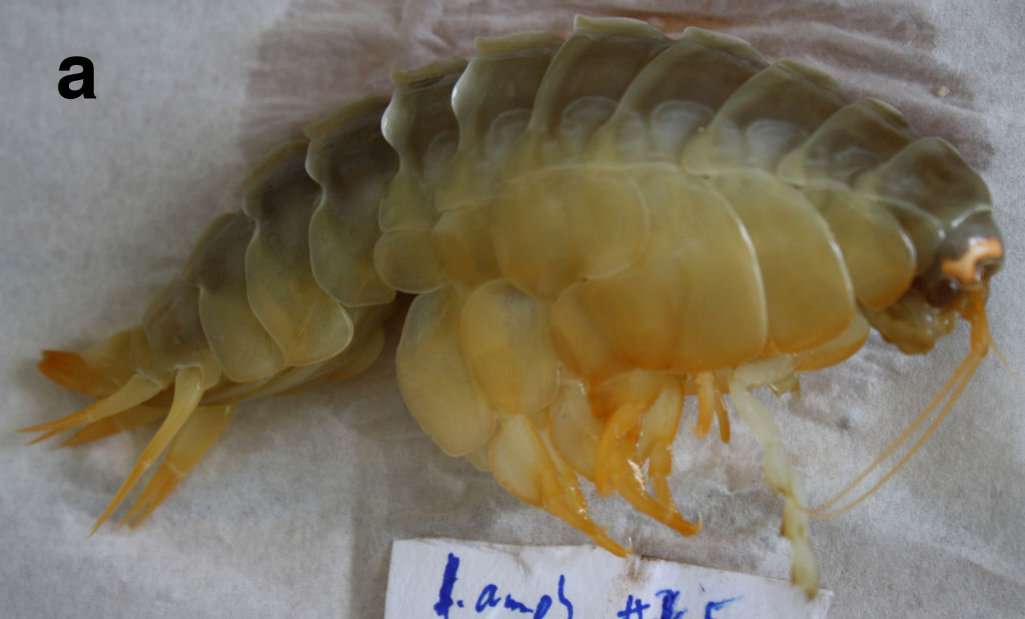
Side view of *Eurythenes magellanicus* after collection. Image attribution: A Leitner and J Drazen.

**Figure 11b.**
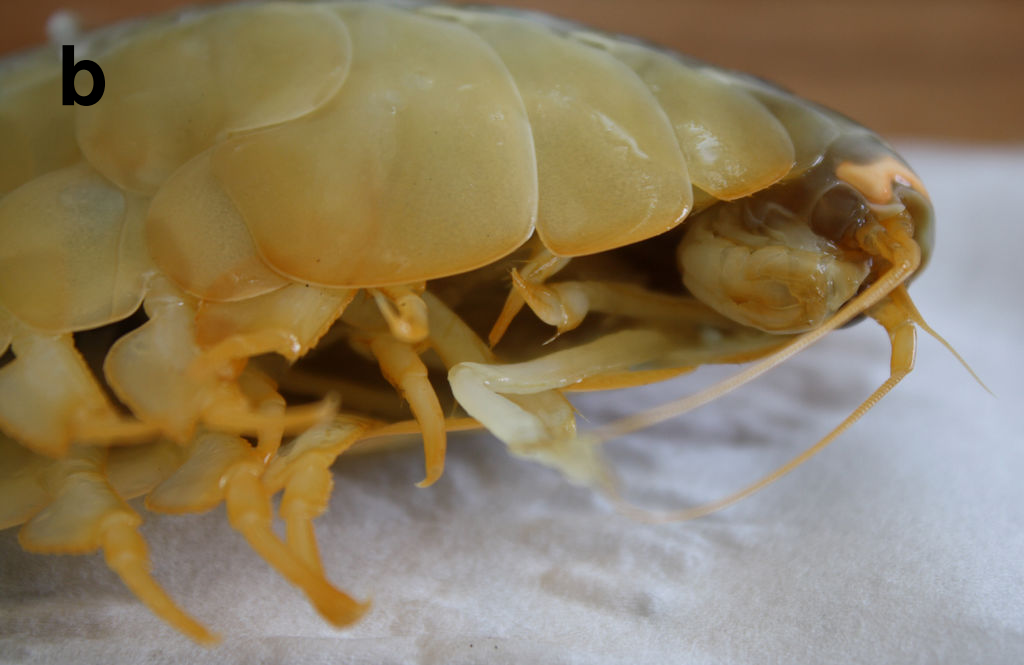
Ventral view of *Eurythenes magellanicus* after collection. Image attribution: A Leitner and J Drazen.

**Figure 11c.**
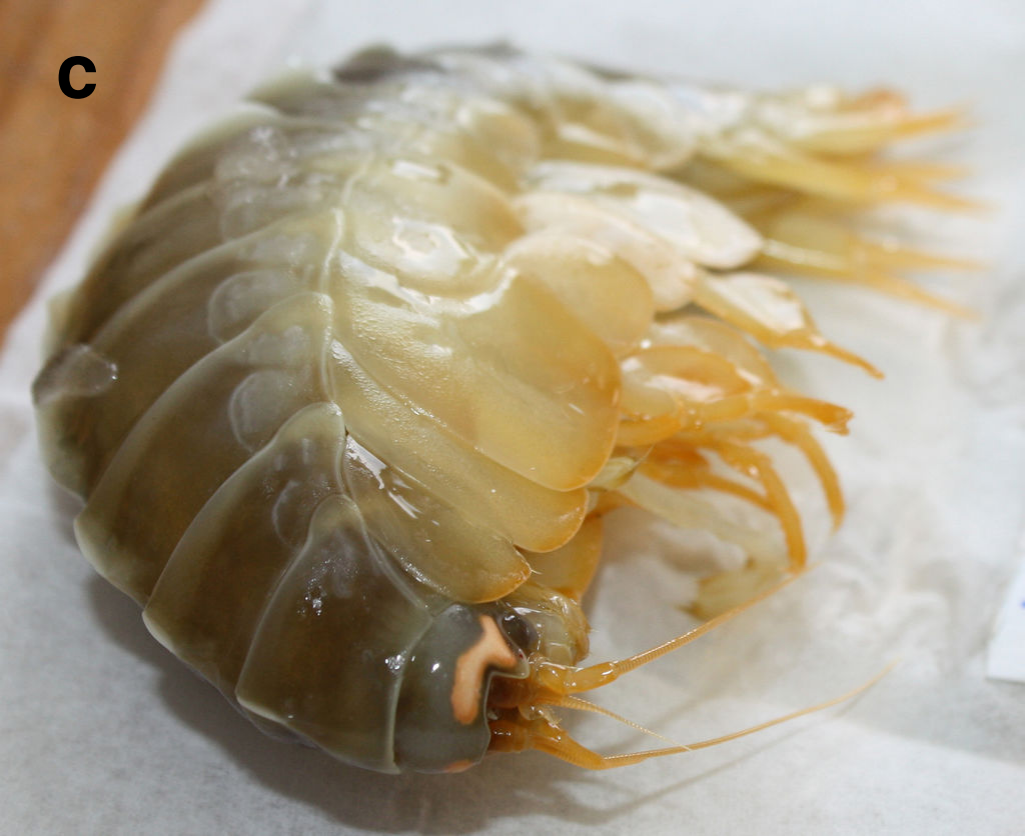
Front view of *Eurythenes magellanicus* after collection. Image attribution: A Leitner and J Drazen.

**Figure 12.**
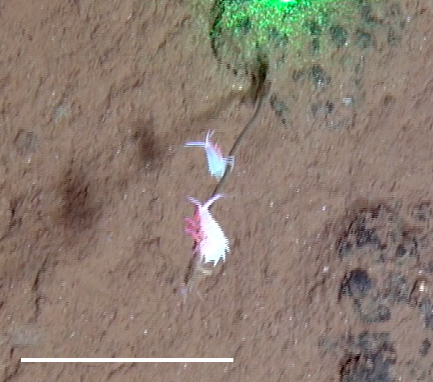
A pair (likely male and female) of cf. Podoceridae morphospecies in situ on a sponge stalk in the UK-1 exploration contract area. Image corresponds with the data above. Scale bar is 10 cm. Image attribution: DJ Amon & CR Smith, University of Hawai’i.

**Figure 13a.**
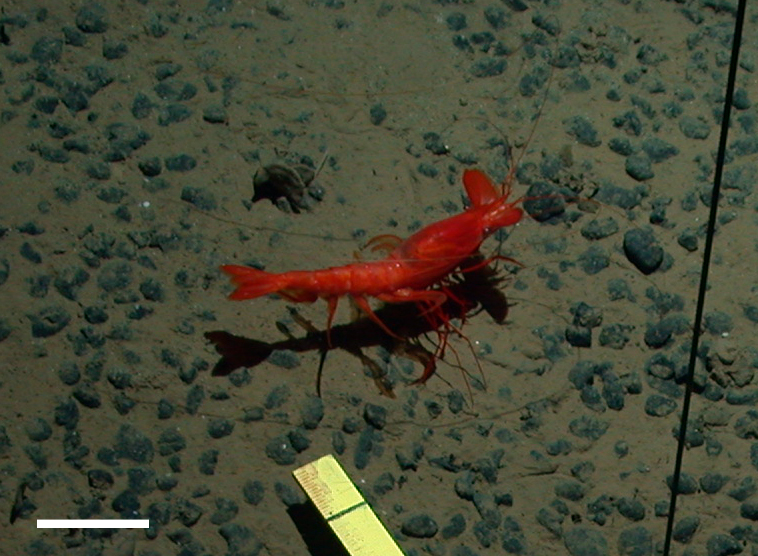
Hemipenaeus cf. spinidorsalis in situ on the seafloor. Scale bar is 10 cm. Image attribution: A Leitner & J Drazen, University of Hawai’i.

**Figure 13b.**
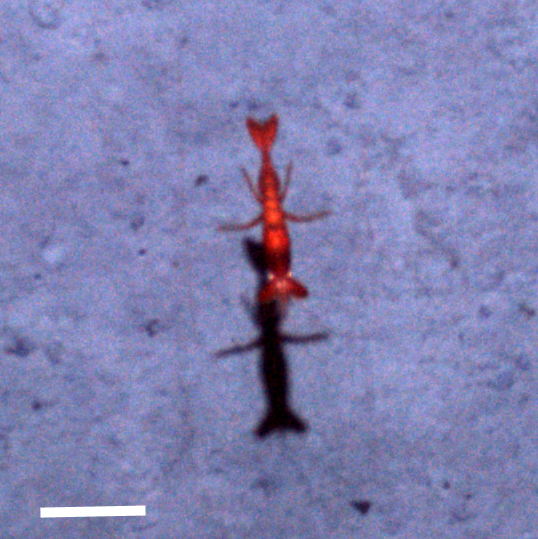
Hemipenaeus cf. spinidorsalis swimming above the seafloor. Scale bar is 10 cm. Image attribution: Woods Hole Oceanographic Institution.

**Figure 13c.**
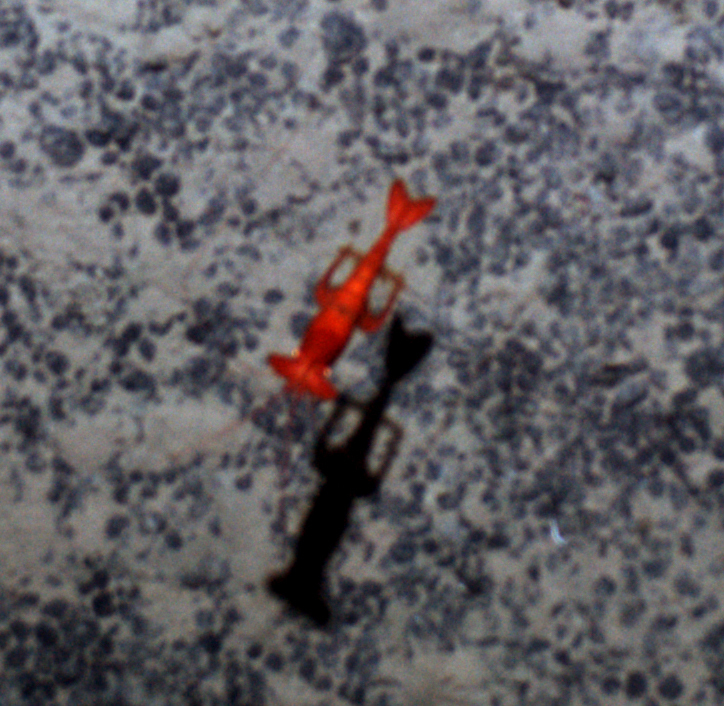
Hemipenaeus cf. spinidorsalis swimming above the seafloor. Image attribution: Woods Hole Oceanographic Institution.

**Figure 13d.**
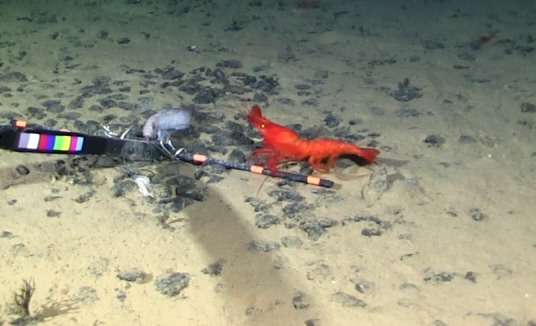
Hemipenaeus cf. spinidorsalis in situ on the seafloor. Image attribution: A Leitner & J Drazen, University of Hawai’i.

**Figure 13e.**
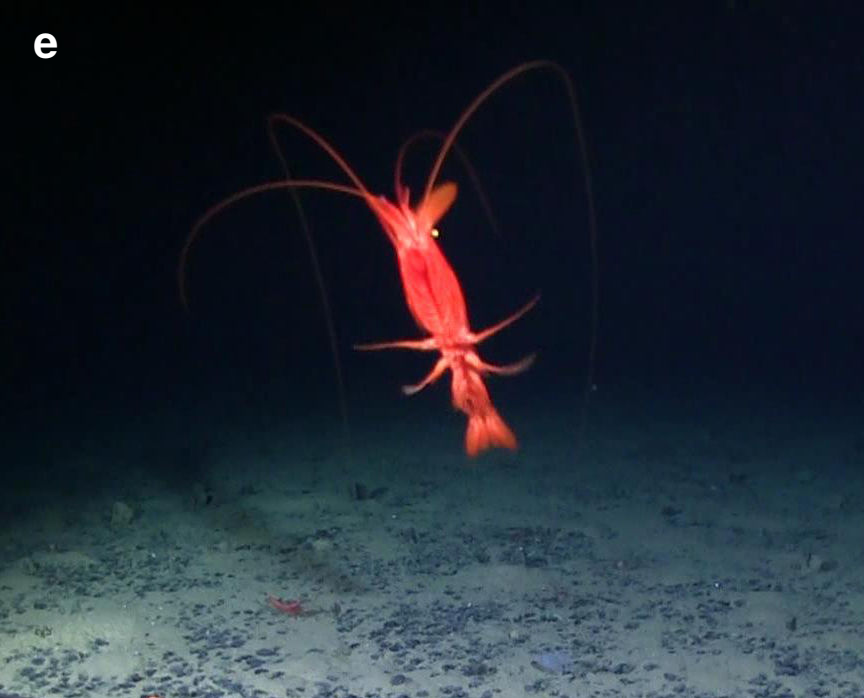
Hemipenaeus cf. spinidorsalis swimming above the seafloor. Image attribution: A Leitner & J Drazen, University of Hawai’i.

**Figure 14.**
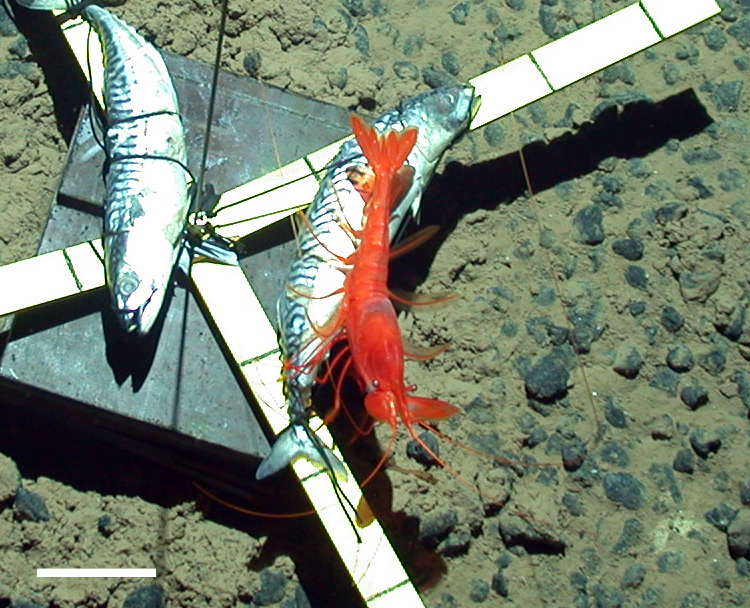
Cerataspis cf. monstrosus in situ feeding on bait on the seafloor in the UK-1 exploration contract area. Image corresponds with the data above. Scale bar is 10 cm. Image attribution: A Leitner and J Drazen, University of Hawai'i.

**Figure 15.**
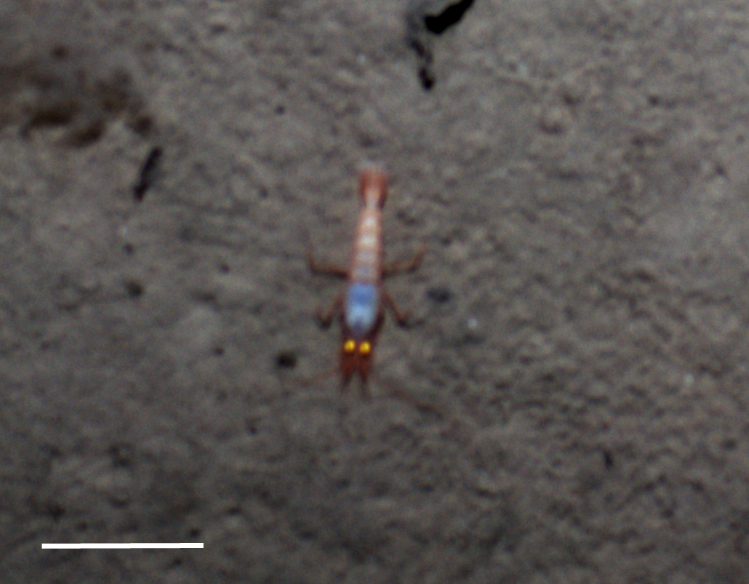
cf. *Glyphocrangon* morphospecies in situ on the seafloor in the UK-1 exploration contract area. Image corresponds with the data above. Scale bar is 10 cm. Image attribution: Woods Hole Oceanographic Institution.

**Figure 16.**
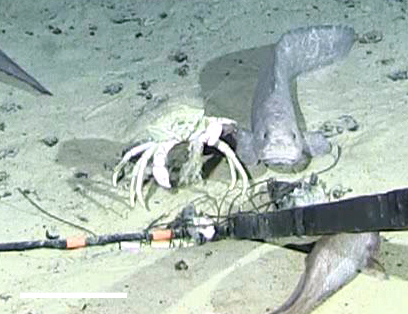
cf. *Munidopsis* morphospecies with several Pachycara cf. nazca in situ on the seafloor in the UK-1 exploration contract area. Image corresponds with the data above. Scale bar is 10 cm. Image attribution: A Leitner and J Drazen, University of Hawai’i.

**Figure 17a.**
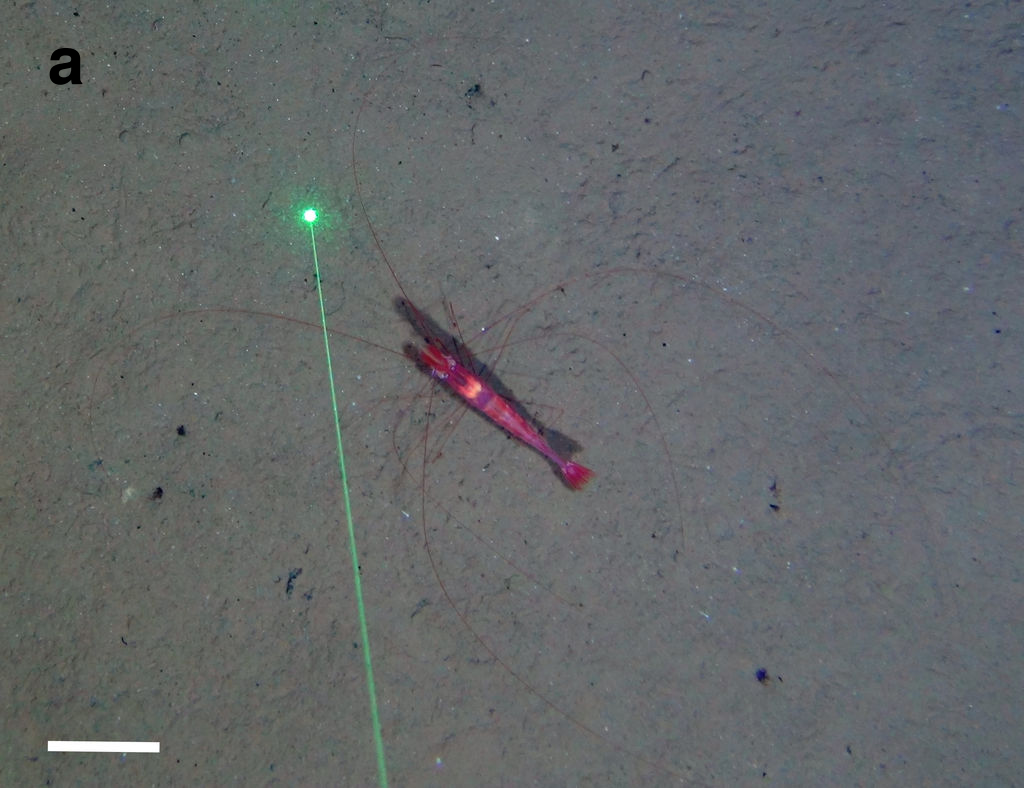
cf. *Nematocarcinus* morphospecies in situ on the seafloor. Scale bar is 10 cm. Image attribution: DJ Amon & CR Smith, University of Hawai’i.

**Figure 17b.**
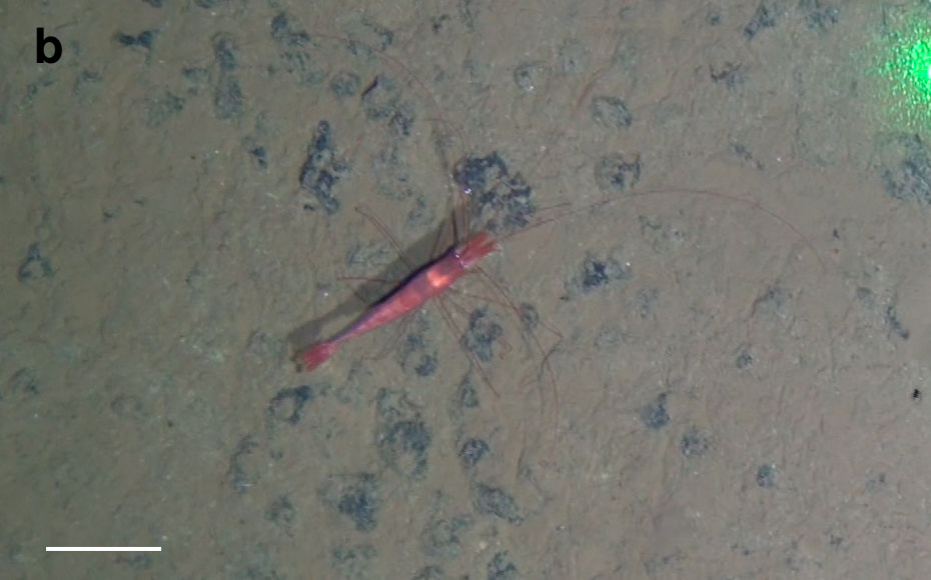
cf. *Nematocarcinus* morphospecies in situ on the seafloor. Scale bar is 10 cm. Image attribution: DJ Amon & CR Smith, University of Hawai’i.

**Figure 18a.**
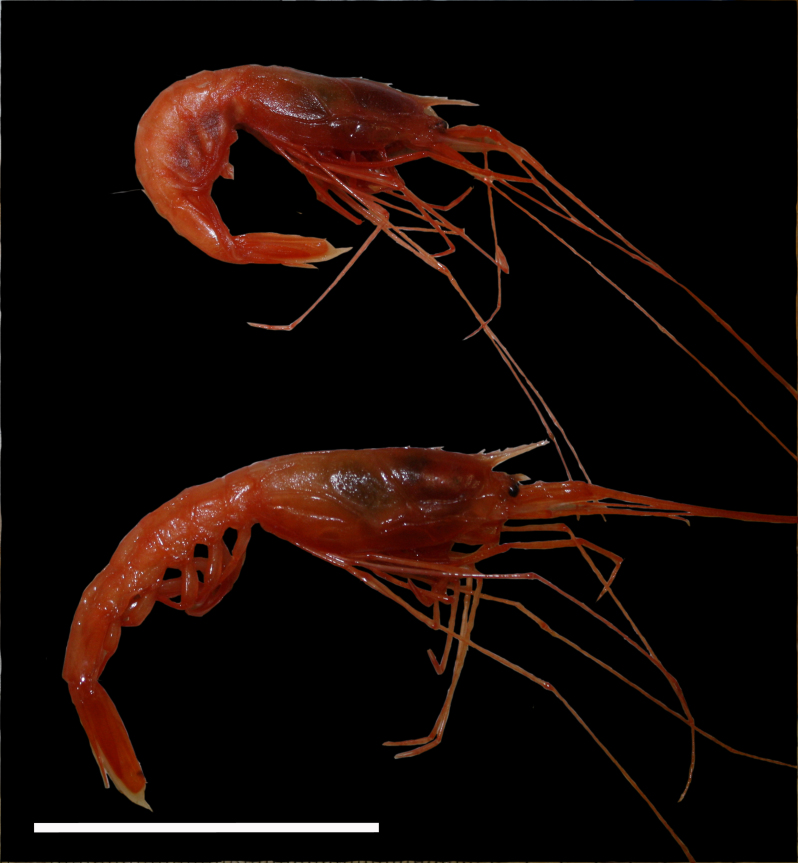
*Hymenopenaeus nereus* after collection via baited trap from the UK-1 exploration contract area. Scale bar is 10 cm. Image attribution: A Leitner and J Drazen, University of Hawai’i.

**Figure 18b.**
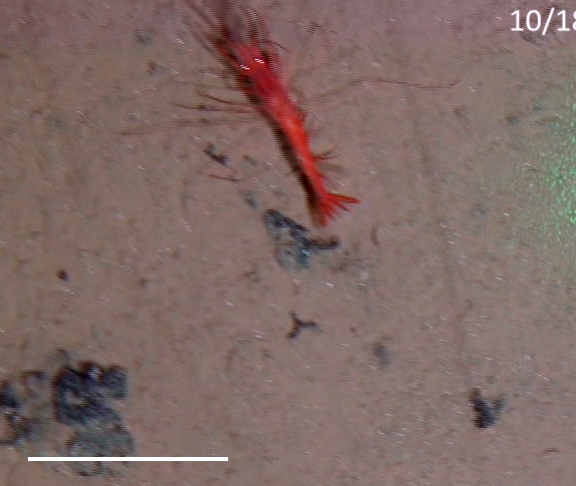
*Hymenopenaeus cf. nereus* in situ on the seafloor. Scale bar is 10 cm. Image attribution: DJ Amon and CR Smith, University of Hawai’i.

**Figure 18c.**
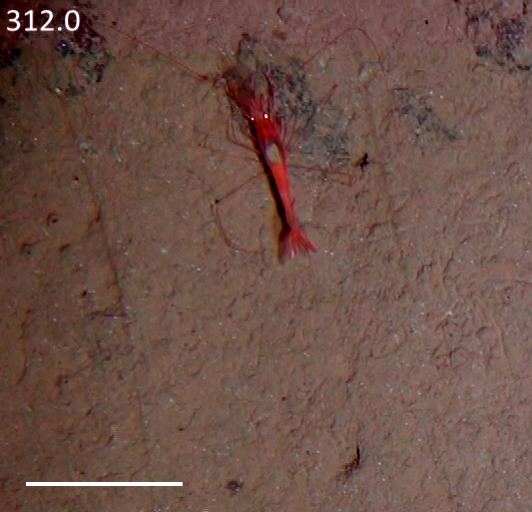
*Hymenopenaeus cf. nereus* in situ on the seafloor. Scale bar is 10 cm. Image attribution: DJ Amon and CR Smith, University of Hawai’i.

**Figure 18d.**
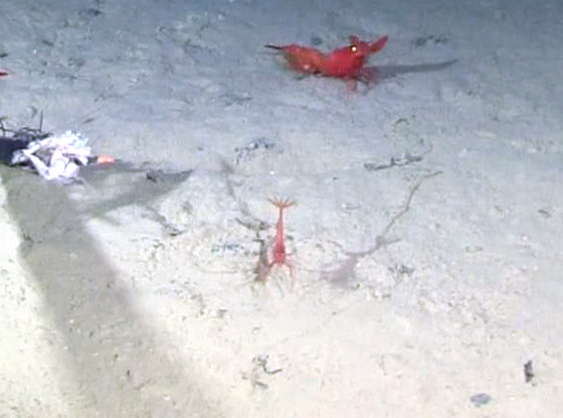
*Hymenopenaeus cf. nereus* in situ on the seafloor (centre of image) with an aristeid shrimp (nearest to top of image). Image attribution: A Leitner and J Drazen, University of Hawai’i.

**Figure 19.**
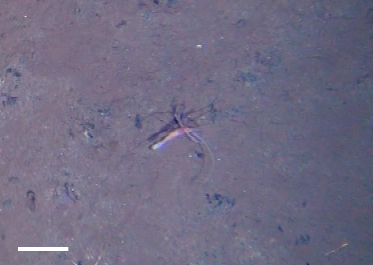
cf. *Bathystylodactylus* morphospecies in situ on the seafloor in the UK-1 exploration contract area. Image corresponds with the data above. Scale bar is 10 cm. Image attribution: Woods Hole Oceanographic Institution.

**Figure 20a.**
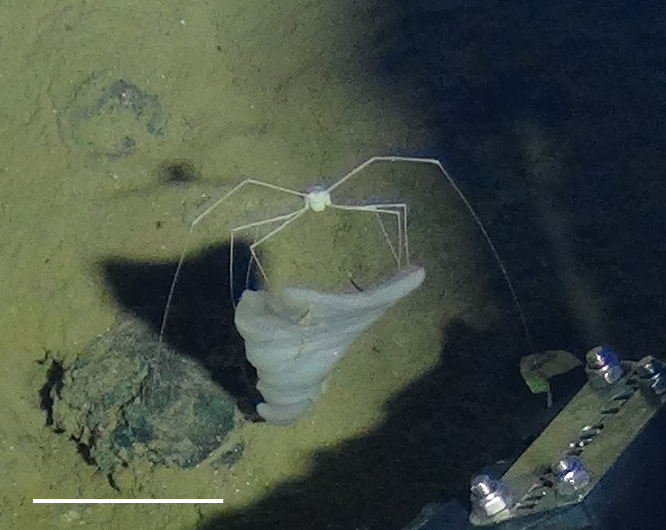
Front view of cf. Munnopsidae morphospecies in situ on a sponge. Scale bar is 10 cm. Image attribution: DJ Amon & CR Smith, University of Hawai’i.

**Figure 20b.**
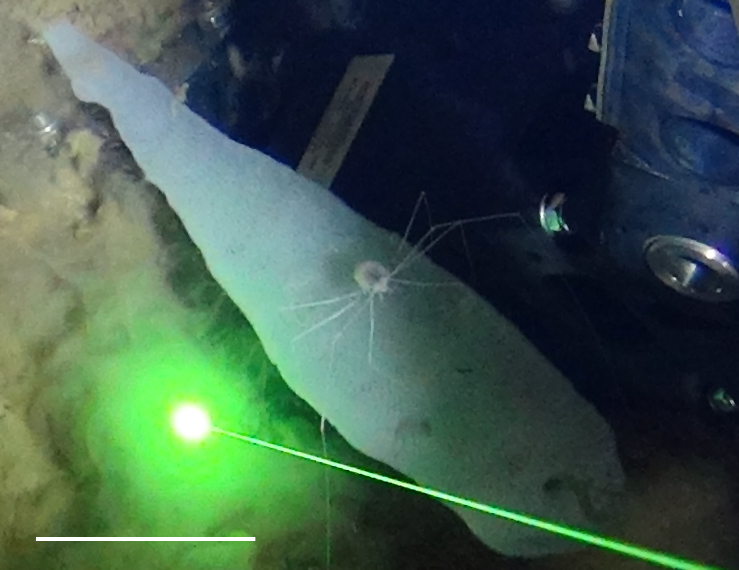
Dorsal view of cf. Munnopsidae morphospecies in situ on a sponge. Scale bar is 10 cm. Image attribution: DJ Amon & CR Smith, University of Hawai’i.

**Figure 20c.**
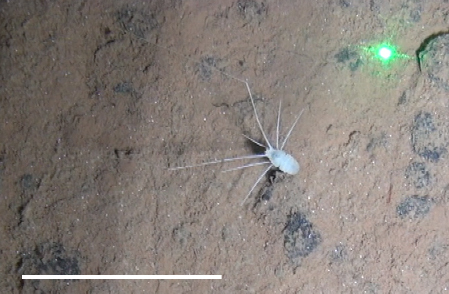
cf. Munnopsidae morphospecies in situ on the seafloor. Scale bar is 10 cm. Image attribution: DJ Amon & CR Smith, University of Hawai’i.

**Figure 21.**
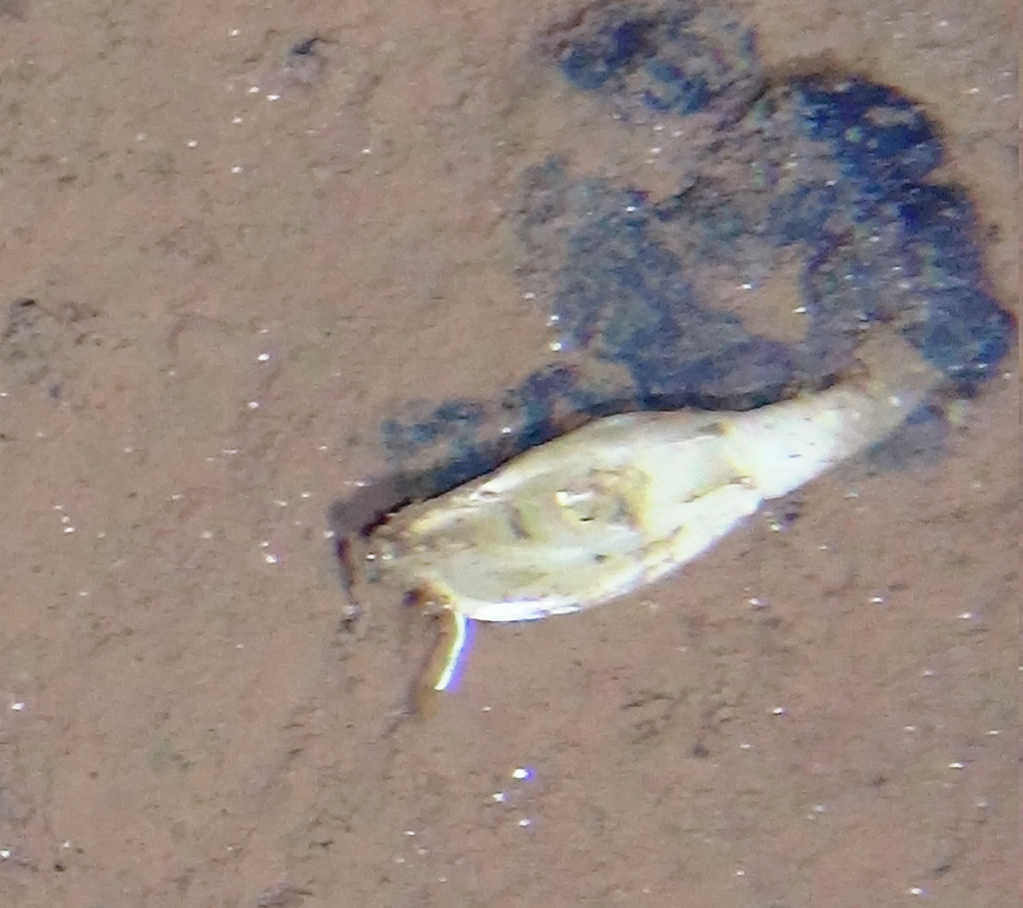
cf. *Neoscalpellum* morphospecies attached to a polymetallic nodule on the seafloor in the UK-1 exploration contract area. Image corresponds with the data above. Image attribution: DJ Amon & CR Smith, University of Hawai’i.

**Figure 22a.**
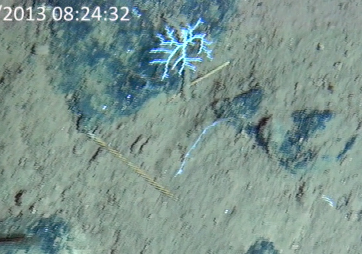
cf. *Smithsonius* morphospecies attached to a polymetallic nodule on the seafloor. Image attribution: DJ Amon & CR Smith, University of Hawai’i.

**Figure 22b.**
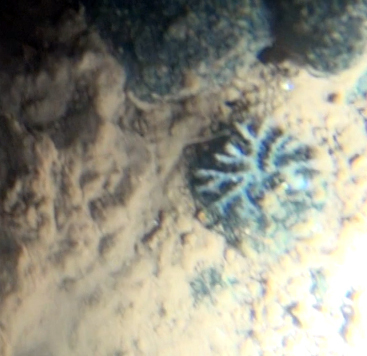
cf. *Smithsonius* morphospecies attached to a polymetallic nodule on the seafloor. Image attribution: DJ Amon & CR Smith, University of Hawai’i.

**Figure 22c.**
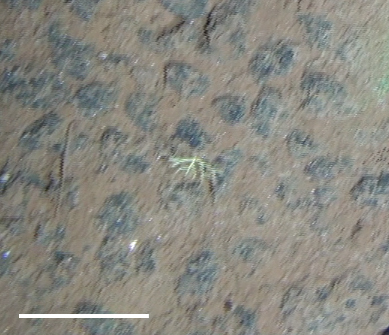
cf. *Smithsonius* morphospecies attached to a polymetallic nodule on the seafloor. Scale bar is 10 cm. Image attribution: DJ Amon & CR Smith, University of Hawai’i.

**Figure 22d.**
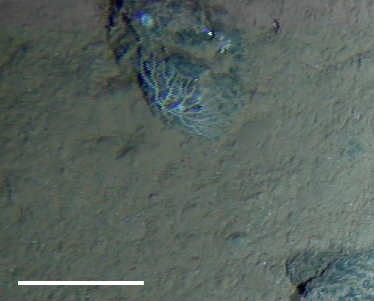
cf. *Smithsonius* morphospecies attached to a polymetallic nodule on the seafloor. Scale bar is 10 cm. Image attribution: DJ Amon & CR Smith, University of Hawai’i.

**Figure 23a.**
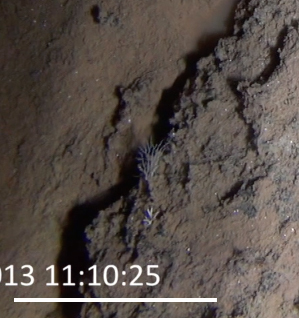
cf. *Notoplites* morphospecies (the branching delicate colony in the centre of image) attached to a polymetallic nodule on the seafloor. Scale bar is 10 cm. Image attribution: DJ Amon & CR Smith, University of Hawai’i.

**Figure 23b.**
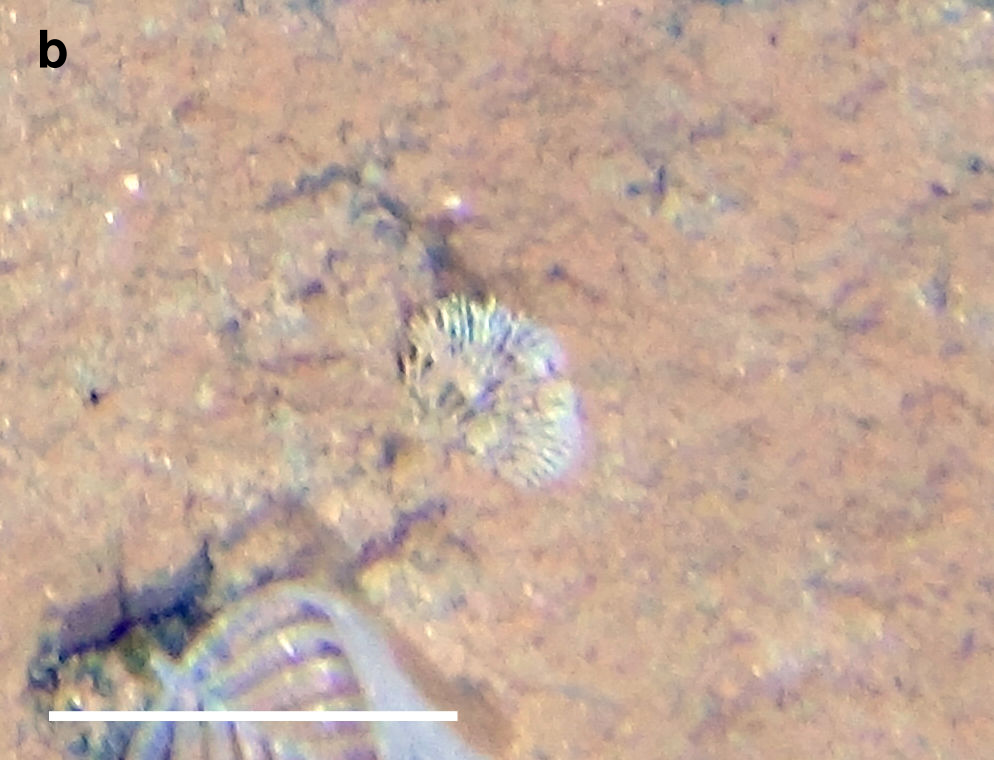
cf. *Notoplites* morphospecies attached to a polymetallic nodule on the seafloor. Scale bar is 10 cm. Image attribution: DJ Amon & CR Smith, University of Hawai’i.

**Figure 23c.**
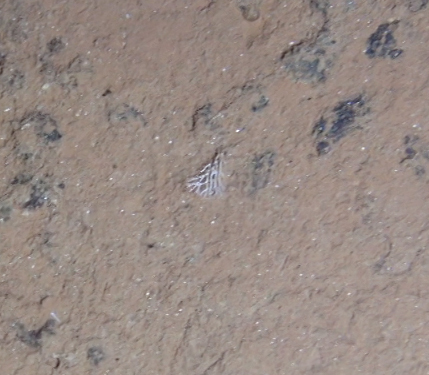
cf. *Notoplites* morphospecies attached to a polymetallic nodule on the seafloor. Image attribution: DJ Amon & CR Smith, University of Hawai’i.

**Figure 23d.**
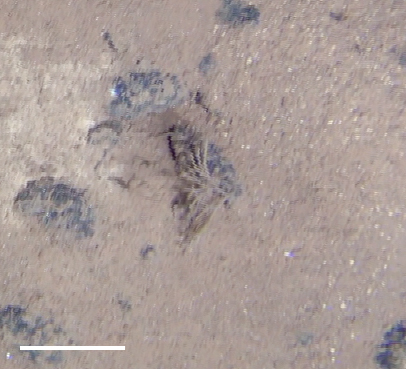
cf. *Notoplites* morphospecies attached to a polymetallic nodule on the seafloor. Scale bar is 10 cm. Image attribution: DJ Amon & CR Smith, University of Hawai’i.

**Figure 24.**
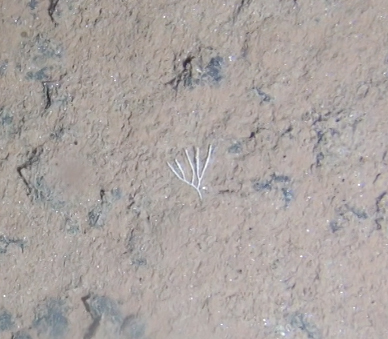
cf. *Columnella* morphospecies attached to a polymetallic nodule on the seafloor in the UK-1 exploration contract area. Image corresponds with the data above. Image attribution: DJ Amon & CR Smith, University of Hawai’i.

**Figure 25.**
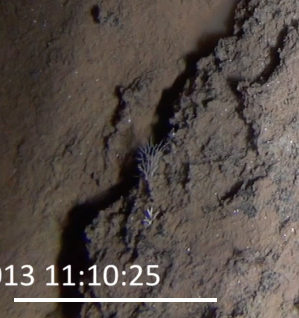
cf. Lepralielloidea morphospecies (small white colony nearest to bottom of the image) attached to a polymetallic nodule on the seafloor in the UK-1 exploration contract area. Image corresponds with the data above. Scale bar is 10 cm. Image attribution: DJ Amon & CR Smith, University of Hawai’i.

**Figure 26.**
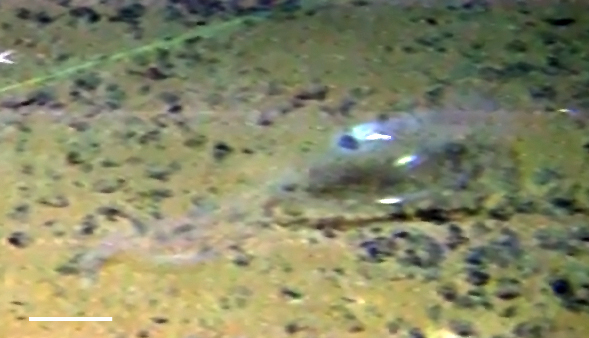
cf. Phlebobranchia morphospecies 1 attached to a polymetallic nodule on the seafloor in the UK-1 exploration contract area. Image corresponds with the data above. Scale bar is 10 cm. Image attribution: DJ Amon & CR Smith, University of Hawai’i.

**Figure 27.**
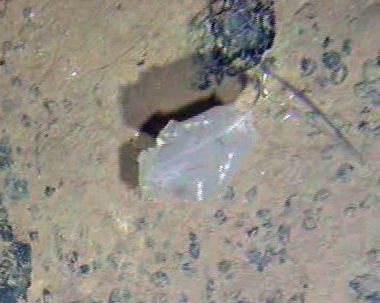
cf. Phlebobranchia morphospecies 2 attached to a polymetallic nodule on the seafloor in the UK-1 exploration contract area. It is attached to another organism that resembles a narrow stalk. Image corresponds with the data above. Image attribution: DJ Amon & CR Smith, University of Hawai’i.

**Figure 28a.**
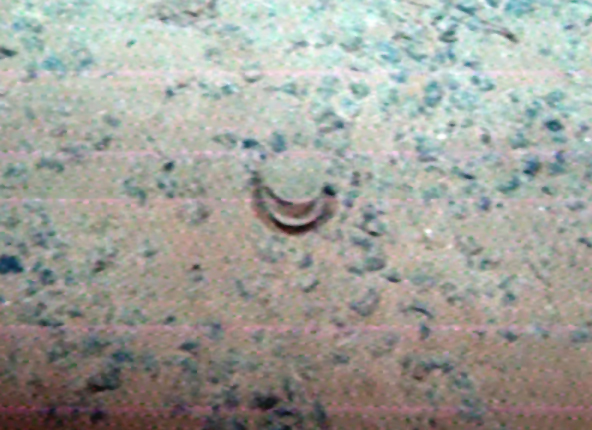
cf. *Dicopia* morphospecies attached to a polymetallic nodule on the seafloor. Image attribution: DJ Amon & CR Smith, University of Hawai’i.

**Figure 28b.**
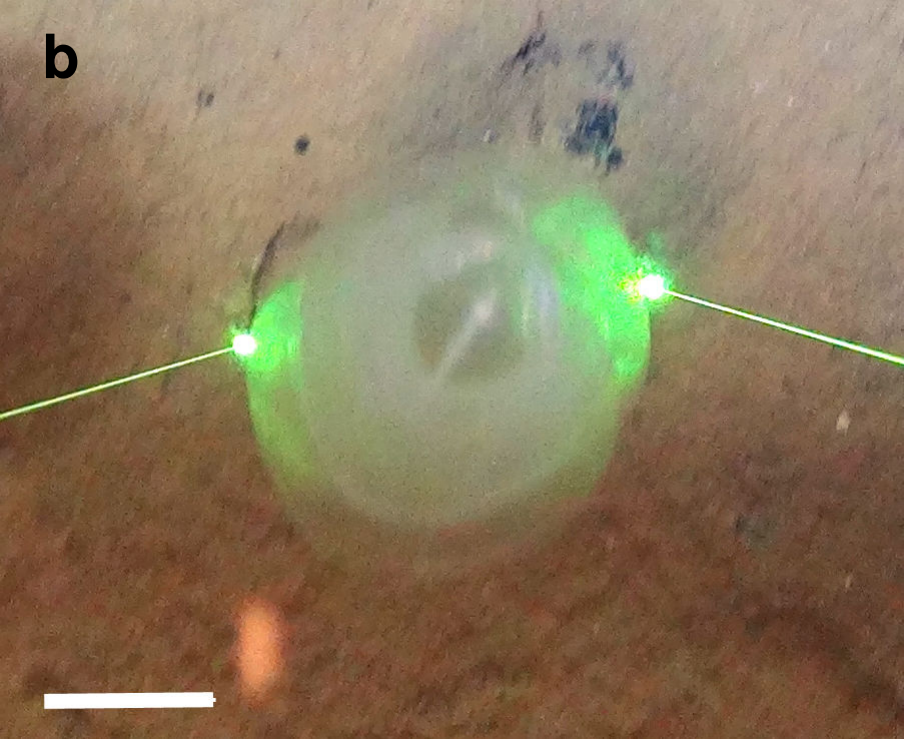
cf. *Dicopia* morphospecies attached to a polymetallic nodule on the seafloor. Image attribution: DJ Amon & CR Smith, University of Hawai’i.

**Figure 28c.**
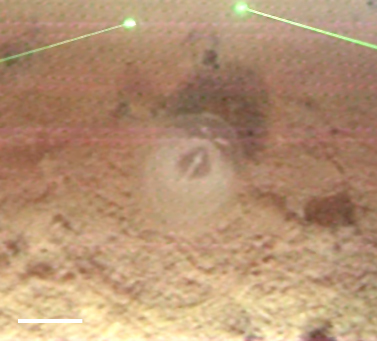
cf. *Dicopia* morphospecies attached to a polymetallic nodule on the seafloor. Image attribution: DJ Amon & CR Smith, University of Hawai’i.

**Figure 29a.**
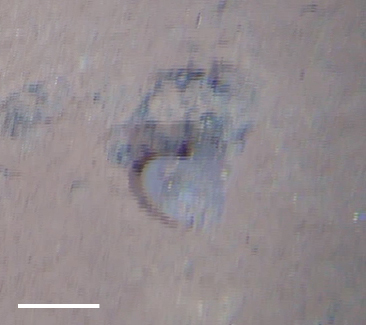
cf. *Megalodicopia* morphospecies attached to a polymetallic nodule on the seafloor. Scale bar is 10 cm. Image attribution: DJ Amon & CR Smith, University of Hawai’i.

**Figure 29b.**
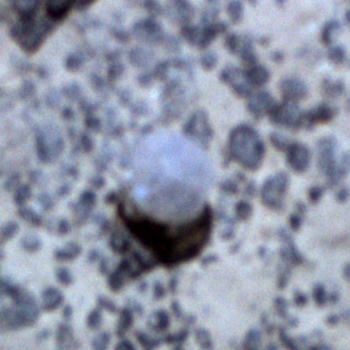
cf. *Megalodicopia* morphospecies attached to a polymetallic nodule on the seafloor. Image attribution: Woods Hole Oceanographic Institution.

**Figure 30a.**
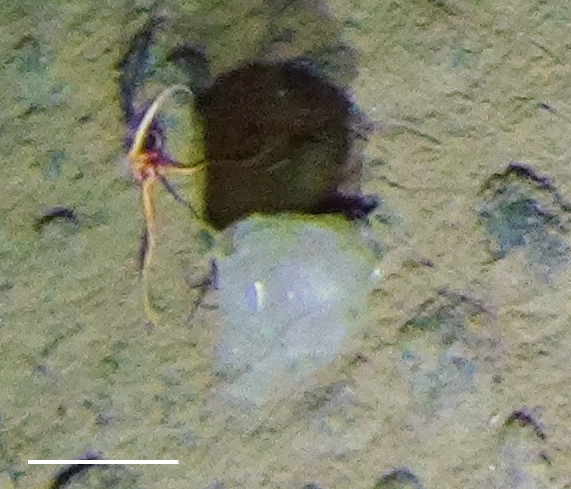
cf. *Situla* morphospecies attached to a polymetallic nodule on the seafloor. Scale bar is 10 cm. Image attribution: DJ Amon & CR Smith, University of Hawai’i.

**Figure 30b.**
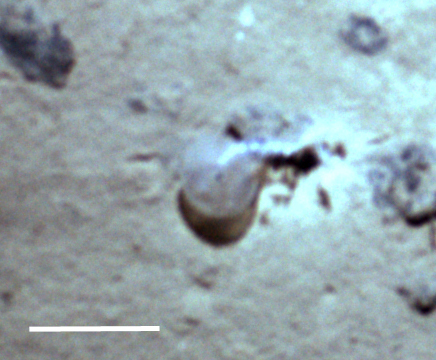
cf. *Situla* morphospecies attached to a polymetallic nodule on the seafloor. Scale bar is 10 cm. Image attribution: Woods Hole Oceanographic Institution.

**Figure 30c.**
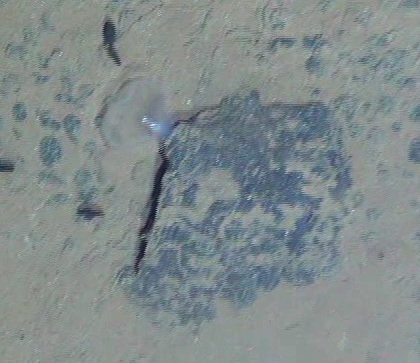
cf. *Situla* morphospecies attached to a polymetallic nodule on the seafloor. Image attribution: DJ Amon & CR Smith, University of Hawai’i.

**Figure 31.**
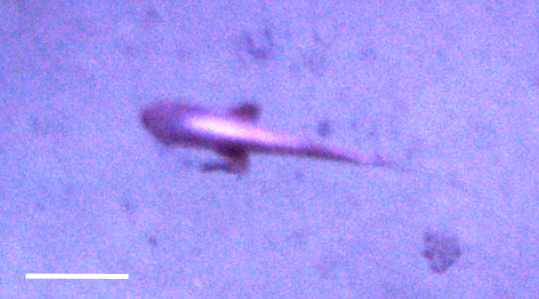
cf. Actinopterygii morphospecies observed swimming above the seafloor in the UK-1 exploration contract area. Image corresponds with the data above. Scale bar is 10 cm. Image attribution: Woods Hole Oceanographic Institution.

**Figure 32a.**
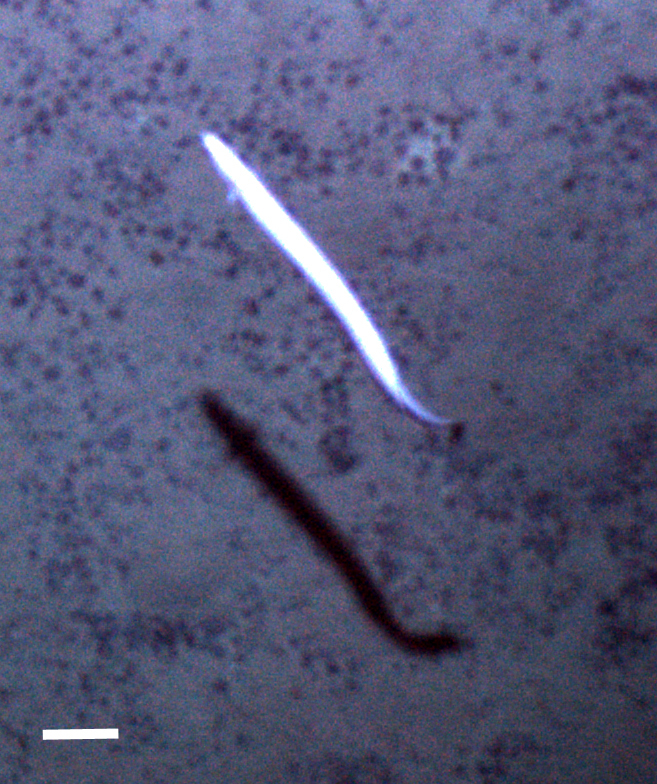
Histiobranchus cf. bathybius swimming above the seafloor. Scale bar is 10 cm. Image attribution: Woods Hole Oceanographic Institution.

**Figure 32b.**
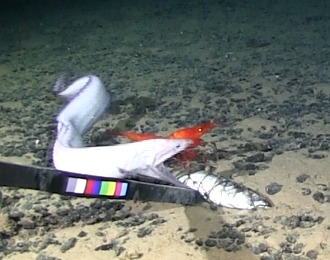
Histiobranchus cf. bathybius in situ feeding on bait on the seafloor. Image attribution: A Leitner & J Drazen, University of Hawai’i.

**Figure 32c.**
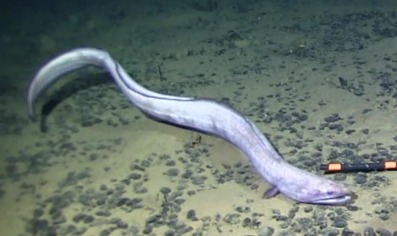
Histiobranchus cf. bathybius feeding on bait on the seafloor. Image attribution: A Leitner & J Drazen, University of Hawai’i.

**Figure 32d.**
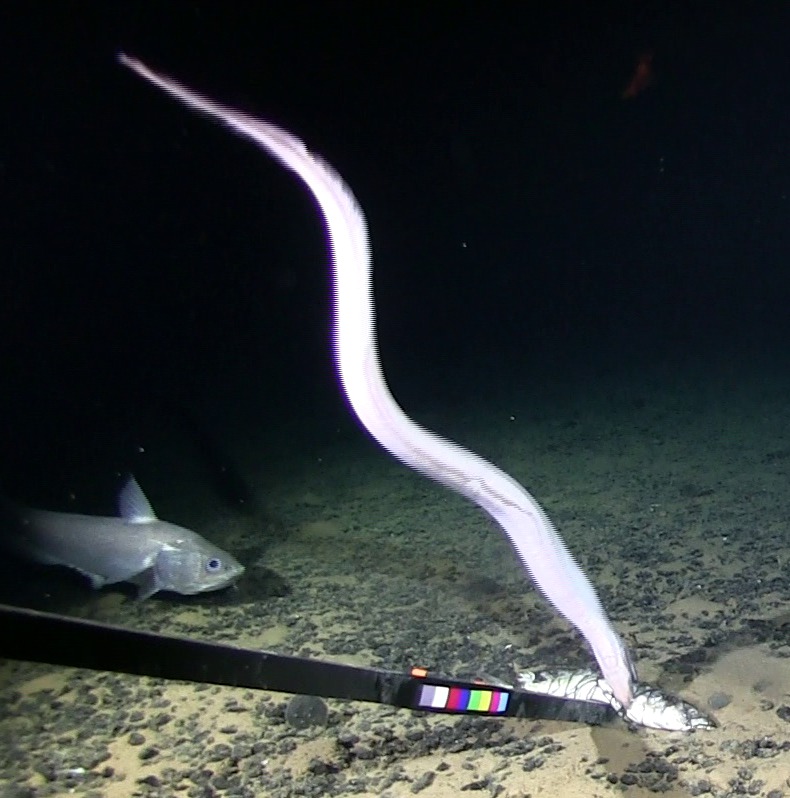
Histiobranchus cf. bathybius (foreground) feeding on bait on the seafloor with a cf. *Coryphaenoides* morphospecies (background). Image attribution: A Leitner & J Drazen, University of Hawai’i.

**Figure 33a.**
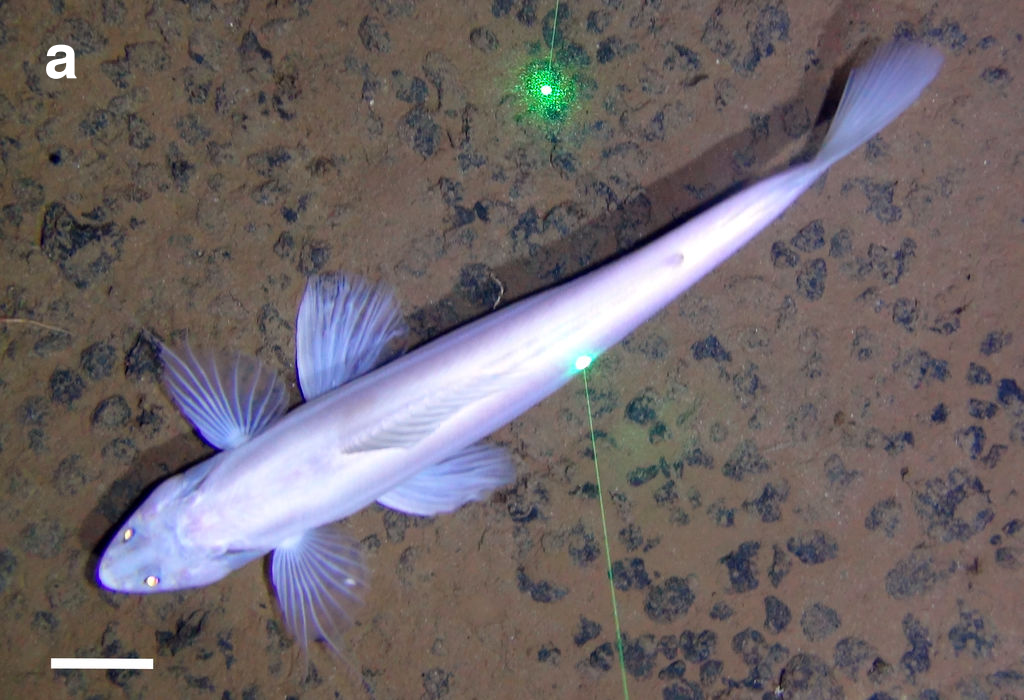
Dorsal view of Bathysaurus cf. mollis swimming above the seafloor. Scale bar is 10 cm. Image attribution: DJ Amon & CR Smith, University of Hawai’i.

**Figure 33b.**
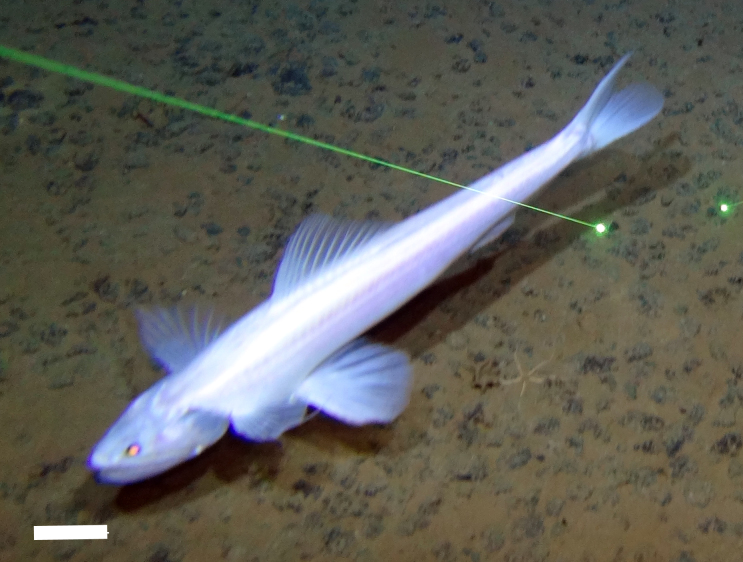
Side view of Bathysaurus cf. mollis swimming above the seafloor. Scale bar is 10 cm. Image attribution: DJ Amon & CR Smith, University of Hawai’i.

**Figure 33c.**
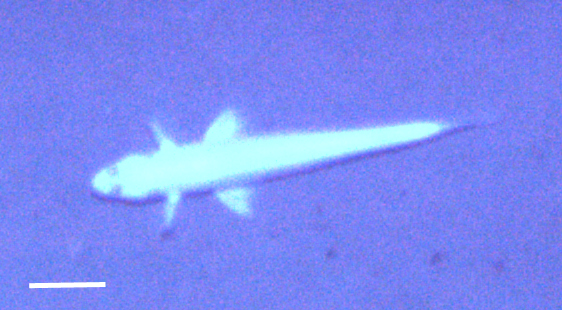
Bathysaurus cf. mollis in situ on the seafloor. Scale bar is 10 cm. Image attribution: Woods Hole Oceanographic Institution.

**Figure 34a.**
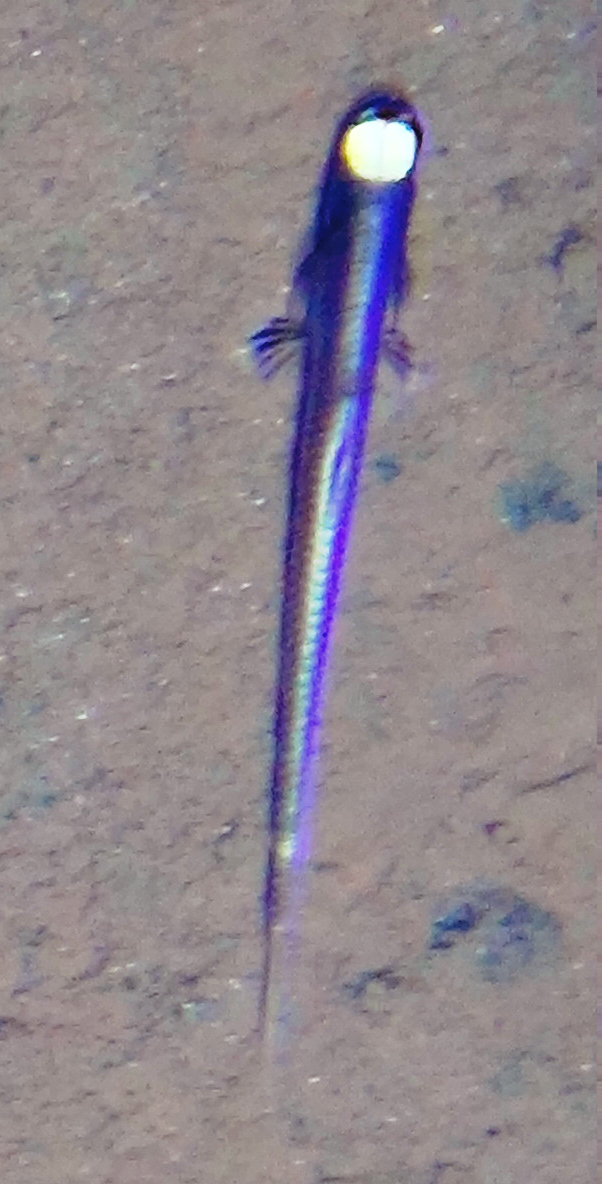
Ipnops cf. meadi in situ on the seafloor. Image attribution: DJ Amon & CR Smith, University of Hawai’i.

**Figure 34b.**
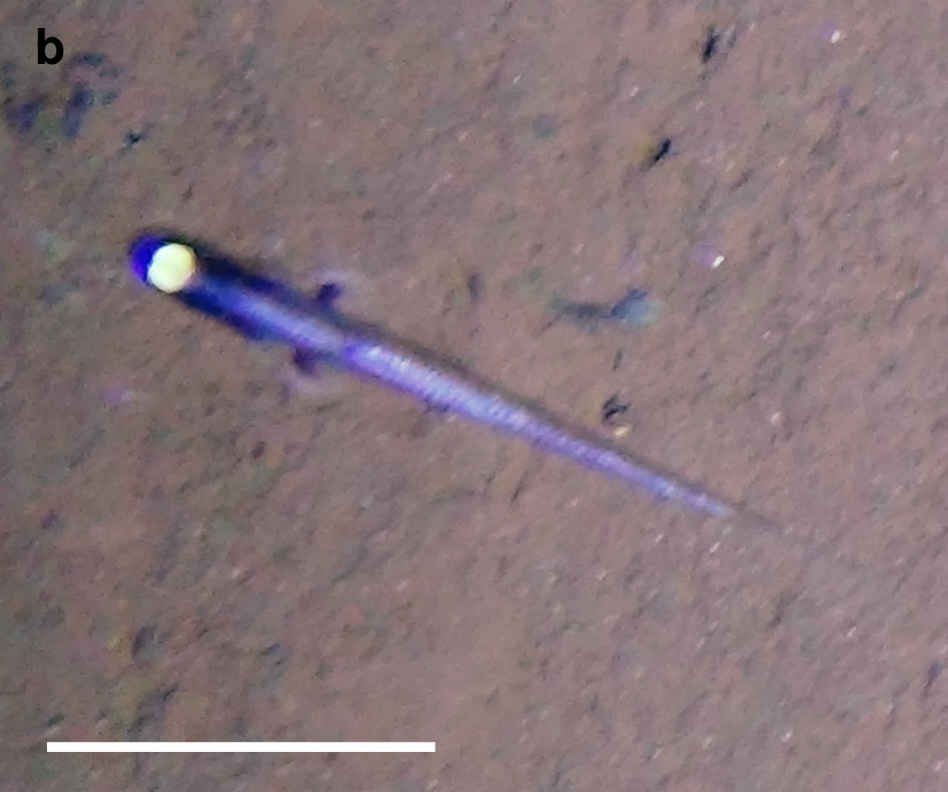
Ipnops cf. meadi in situ on the seafloor. Scale bar is 10 cm. Image attribution: DJ Amon & CR Smith, University of Hawai’i.

**Figure 35a.**
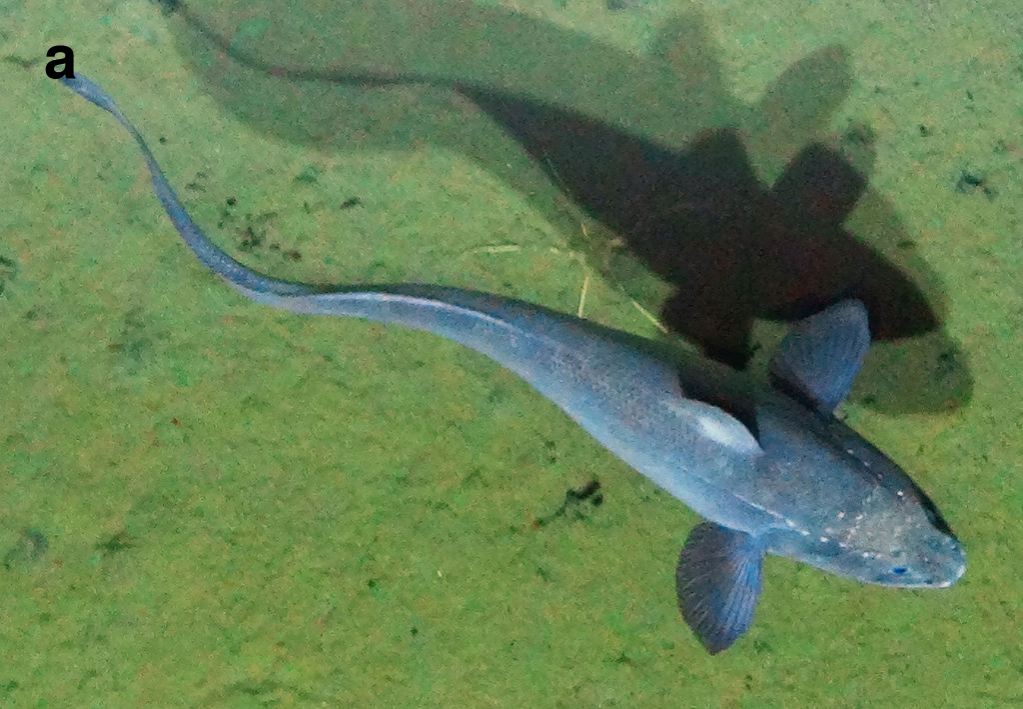
Dorsal view of cf. *Coryphaenoides* morphospecies swimming above the seafloor. Image attribution: DJ Amon & CR Smith, University of Hawai’i.

**Figure 35b.**
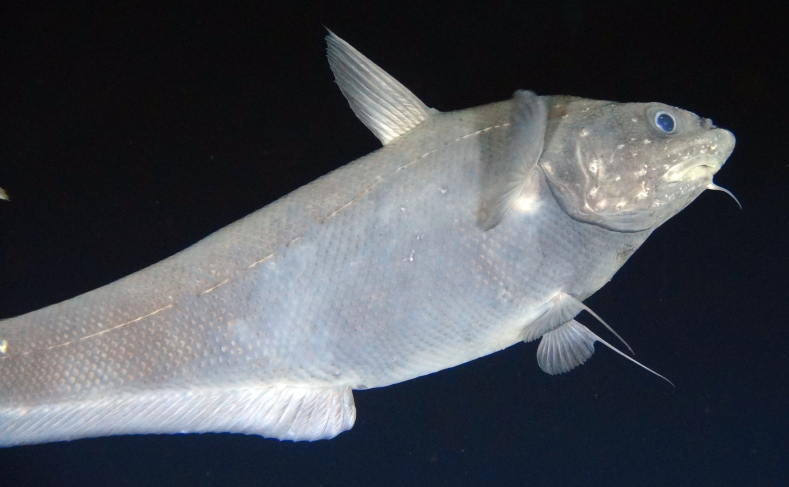
Side view of cf. *Coryphaenoides* morphospecies swimming above the seafloor. Image attribution: DJ Amon & CR Smith, University of Hawai’i.

**Figure 35c.**
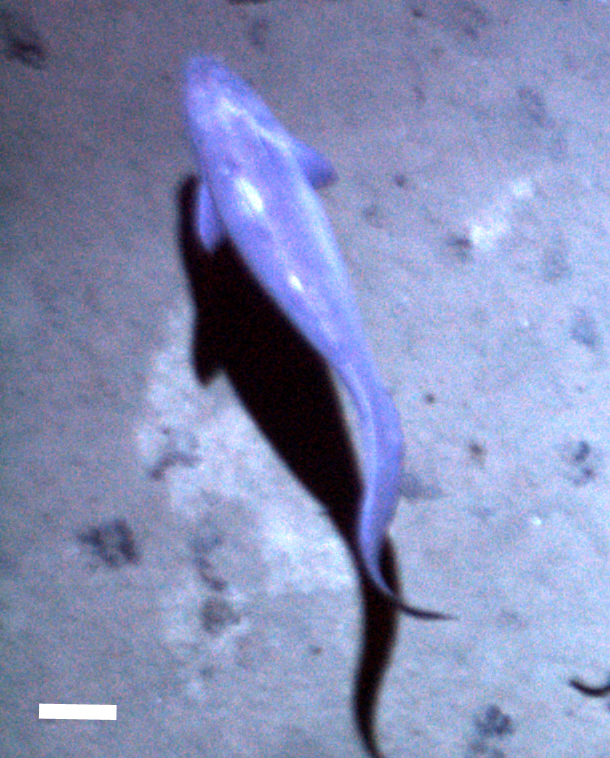
cf. *Coryphaenoides* morphospecies swimming above the seafloor. Scale bar is 10 cm. Image attribution: Woods Hole Oceanographic Institution.

**Figure 35d.**
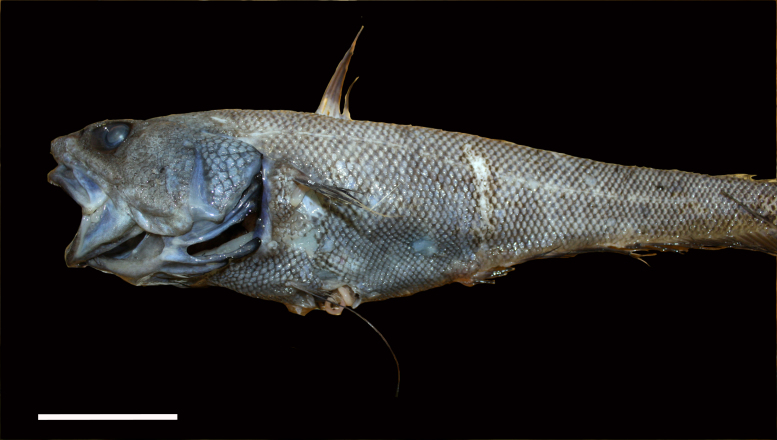
*Coryphaenoides yaquinae* after collection via baited trap from the UK-1 exploration contract area. Scale bar is 10 cm. Image attribution: A Leitner and J Drazen, University of Hawai’i.

**Figure 35e.**
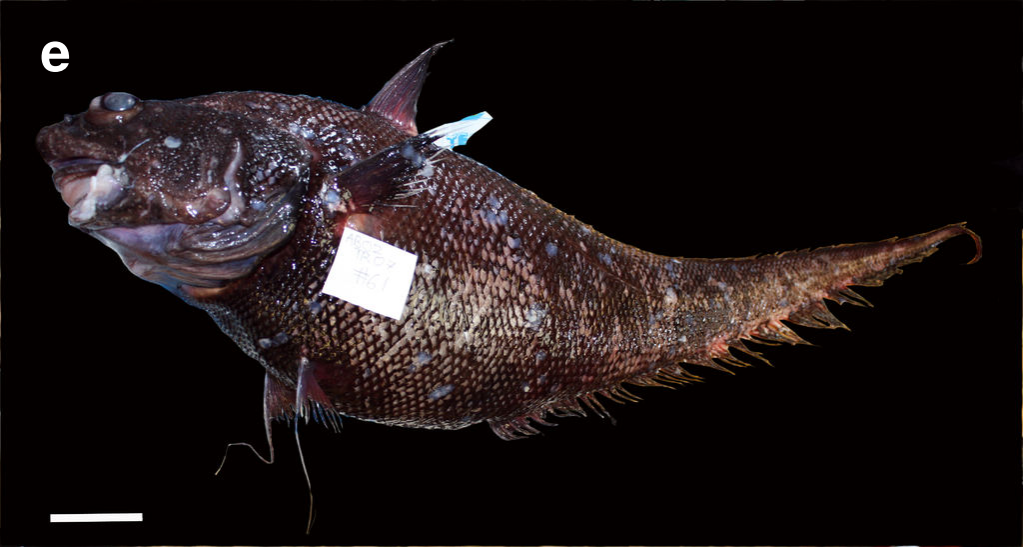
*Coryphaenoides armatus* after collection via baited trap from the UK-1 exploration contract area. Scale bar is 10 cm. Image attribution: A Leitner and J Drazen, University of Hawai’i.

**Figure 35f.**
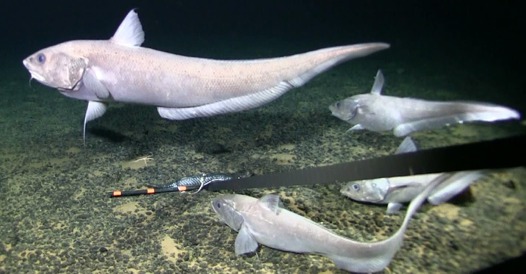
Four cf. *Coryphaenoides* morphospecies swimming above the seafloor. Image attribution: A Leitner and J Drazen, University of Hawai’i.

**Figure 36a.**
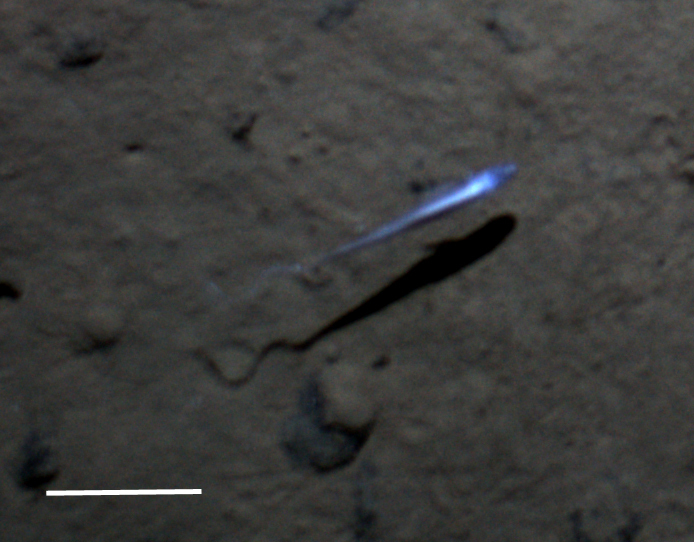
cf. Halosauridae morphospecies swimming above the seafloor. Scale bar is 10 cm. Image attribution: Woods Hole Oceanographic Institution.

**Figure 36b.**
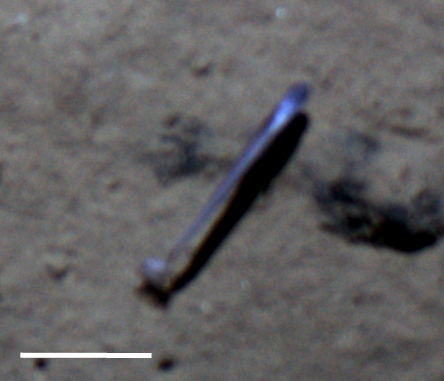
cf. Halosauridae morphospecies swimming above the seafloor. Scale bar is 10 cm. Image attribution: Woods Hole Oceanographic Institution.

**Figure 37a.**
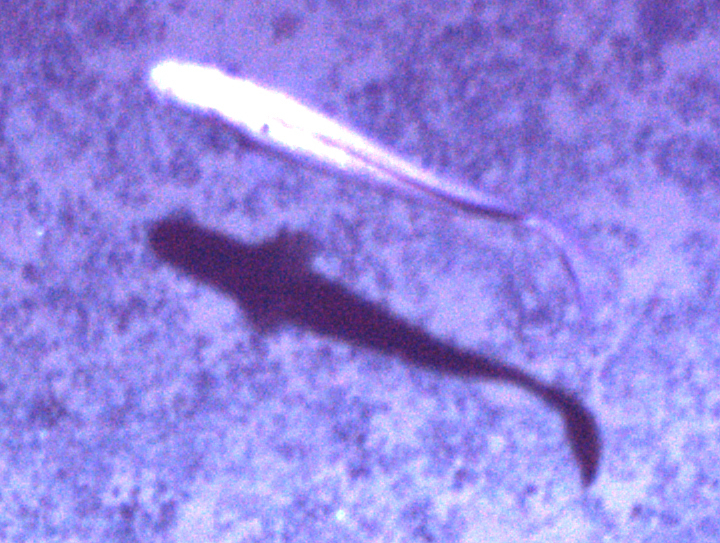
cf. Ophidiidae morphospecies 1 swimming above the seafloor. Image attribution: Woods Hole Oceanographic Institution.

**Figure 37b.**
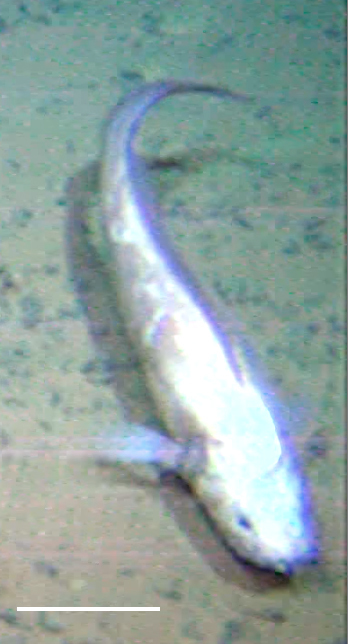
cf. Ophidiidae morphospecies 1 swimming above the seafloor. Scale bar is 10 cm. Image attribution: DJ Amon & CR Smith, University of Hawai’i.

**Figure 38.**
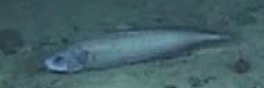
cf. Ophidiidae morphospecies 2 in situ on the seafloor in the UK-1 exploration contract area. Image corresponds with the data above. Image attribution: A Leitner & J Drazen, University of Hawai’i.

**Figure 39.**
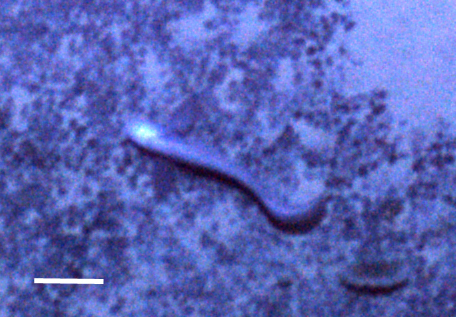
cf. Ophidiidae morphospecies 3 in situ on the seafloor in the UK-1 exploration contract area. Image corresponds with the data above. Scale bar is 10 cm. Image attribution: Woods Hole Oceanographic Institutio.

**Figure 40a.**
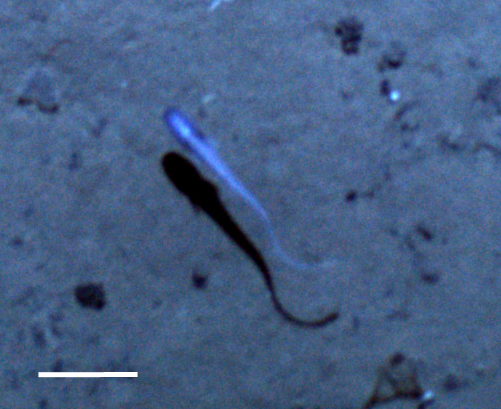
cf. Ophidiidae morphospecies 4 swimming above the seafloor. Scale bar is 10 cm. Image attribution: Woods Hole Oceanographic Institution.

**Figure 40b.**
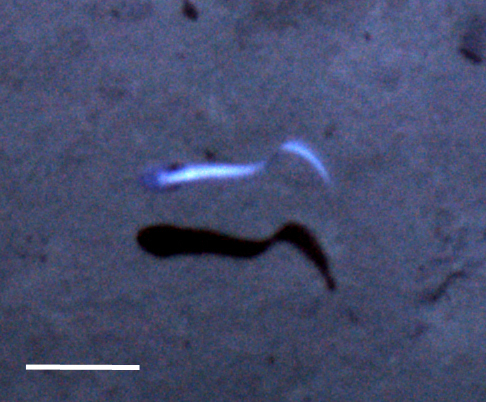
cf. Ophidiidae morphospecies 4 swimming above the seafloor. Scale bar is 10 cm. Image attribution: Woods Hole Oceanographic Institution.

**Figure 41a.**
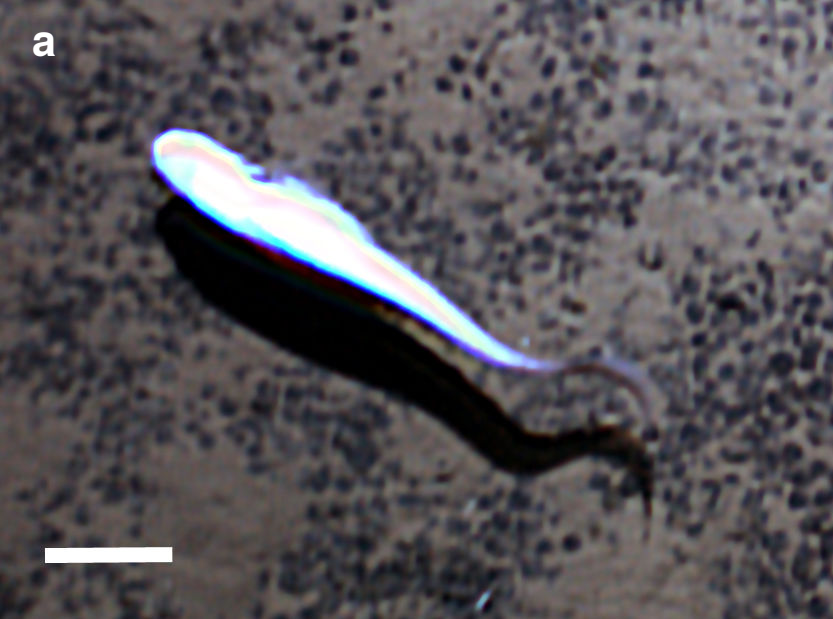
cf. Ophidiidae morphospecies 5 swimming above the seafloor. Scale bar is 10 cm. Image attribution: Woods Hole Oceanographic Institution.

**Figure 41b.**
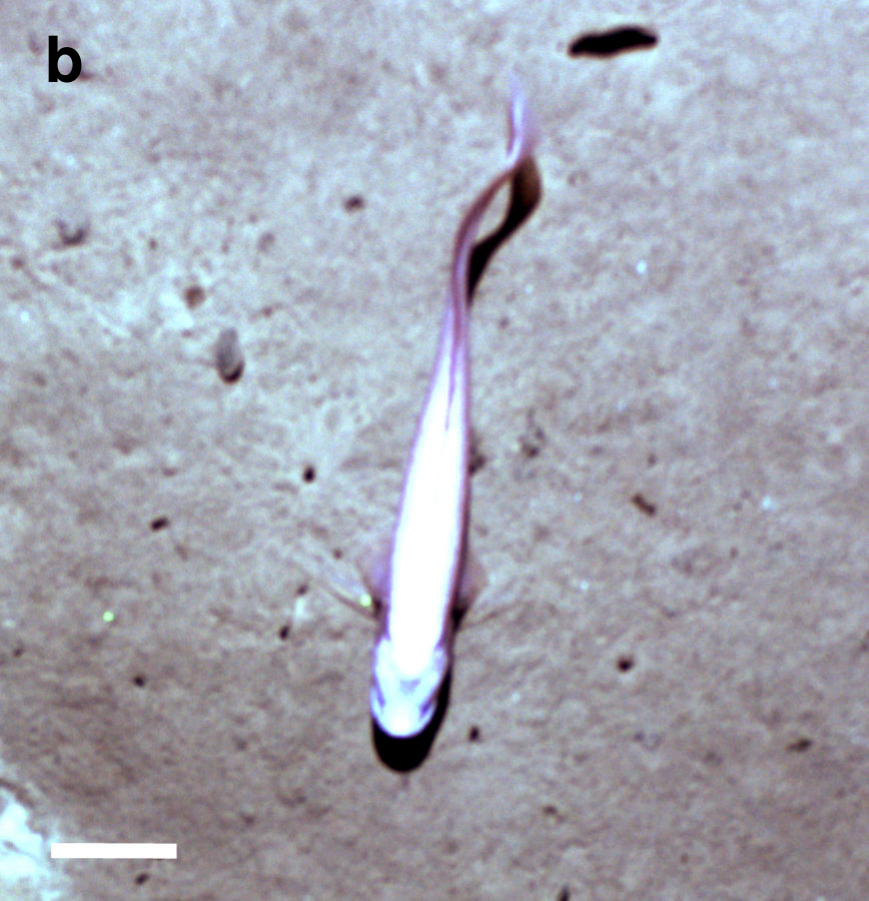
cf. Ophidiidae morphospecies 5 swimming above the seafloor. Scale bar is 10 cm. Image attribution: Woods Hole Oceanographic Institution.

**Figure 42.**
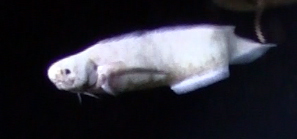
Barathrites cf. iris swimming above the seafloor in the UK-1 exploration contract area. Image corresponds with the data above. Image attribution: A Leitner & J Drazen, University of Hawai’i.

**Figure 43a.**
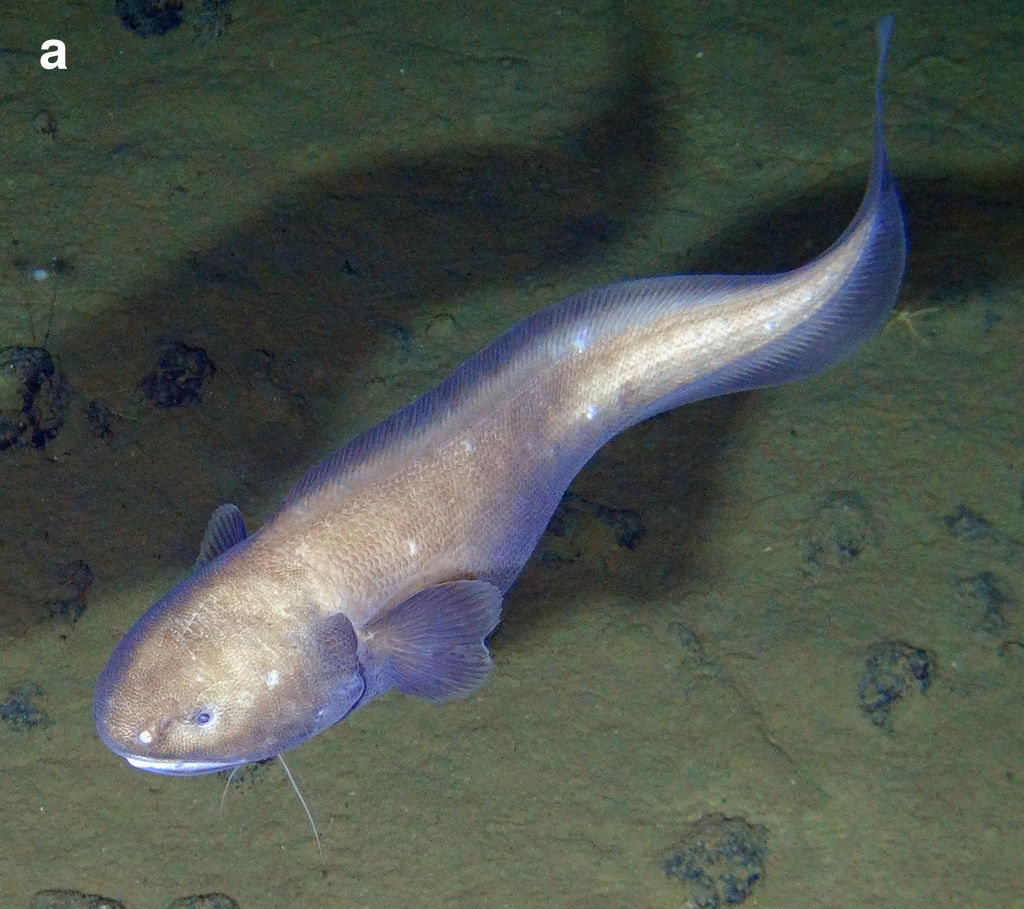
Side view of Bassozetus cf. nasus swimming above the seafloor. Image attribution: DJ Amon & CR Smith, University of Hawai’i.

**Figure 43b.**
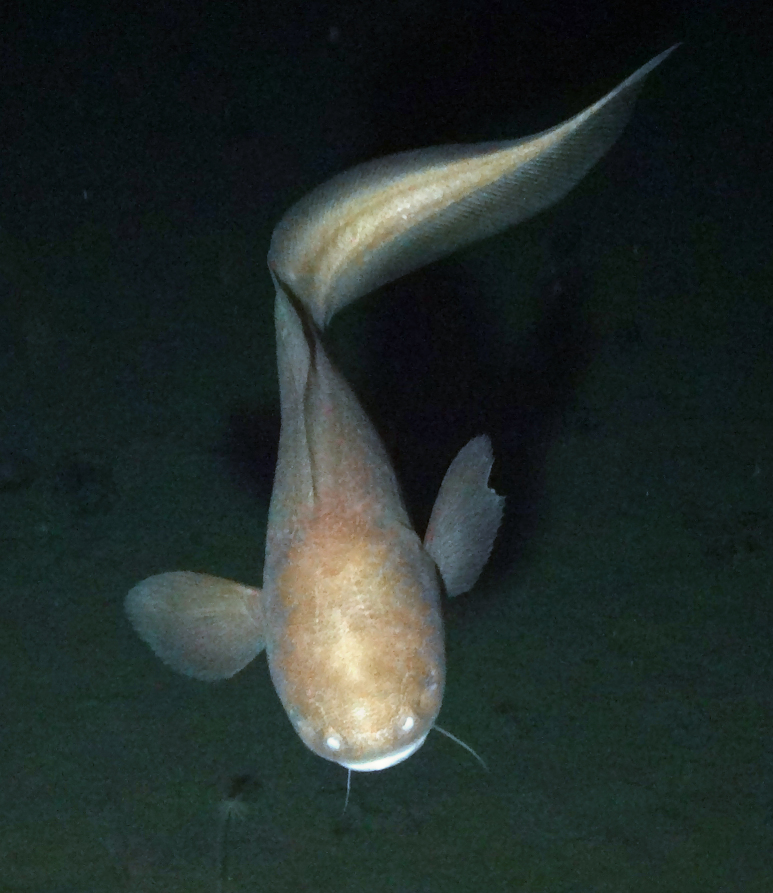
Front view of Bassozetus cf. nasus swimming above the seafloor. Image attribution: DJ Amon & CR Smith, University of Hawai’i.

**Figure 43c.**
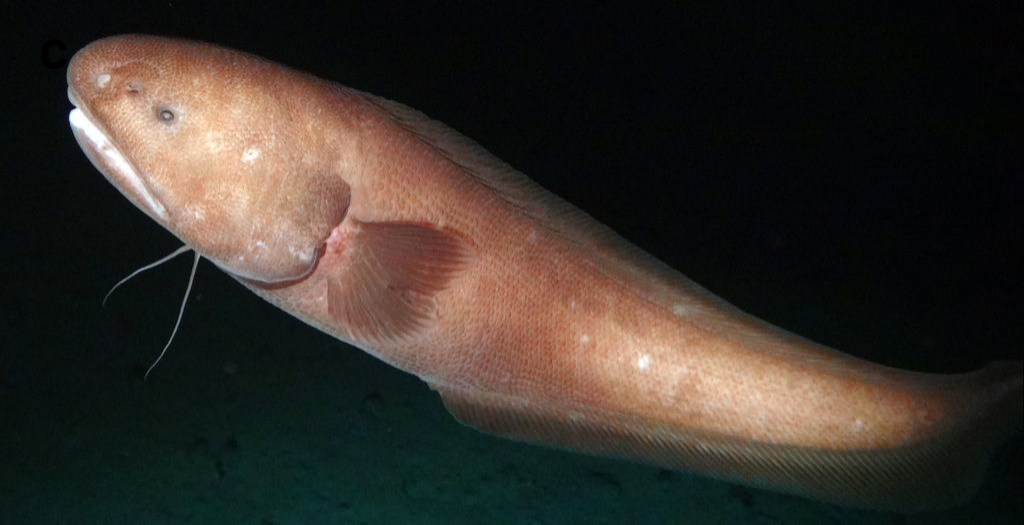
Side view of Bassozetus cf. nasus swimming above the seafloor. Image attribution: DJ Amon & CR Smith, University of Hawai’i.

**Figure 43d.**
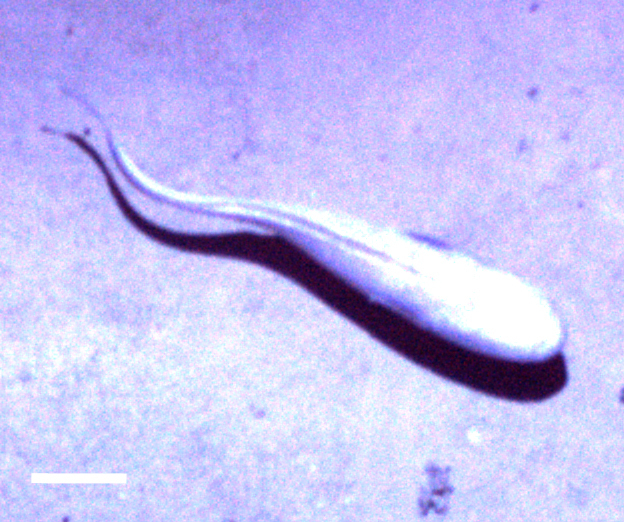
Bassozetus cf. nasus swimming above the seafloor. Scale is 10 cm. Image attribution: Woods Hole Oceanographic Institution.

**Figure 43e.**
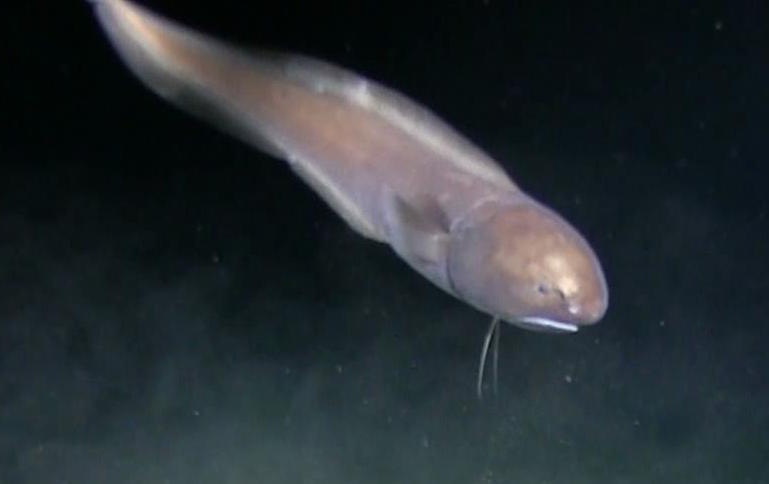
Bassozetus cf. nasus swimming above the seafloor. Image attribution: A Leitner & J Drazen, University of Hawai’i.

**Figure 44a.**
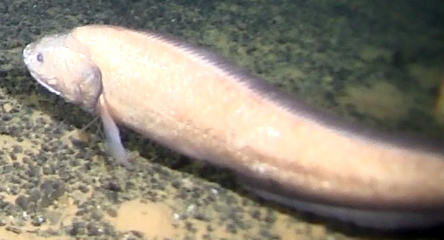
Bathyonus cf. caudalis swimming above the seafloor. Image attribution: A Leitner & J Drazen, University of Hawai’i.

**Figure 44b.**
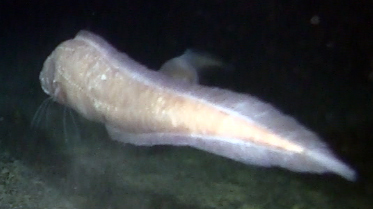
Bathyonus cf. caudalis swimming above the seafloor. Image attribution: A Leitner & J Drazen, University of Hawai’i.

**Figure 44c.**
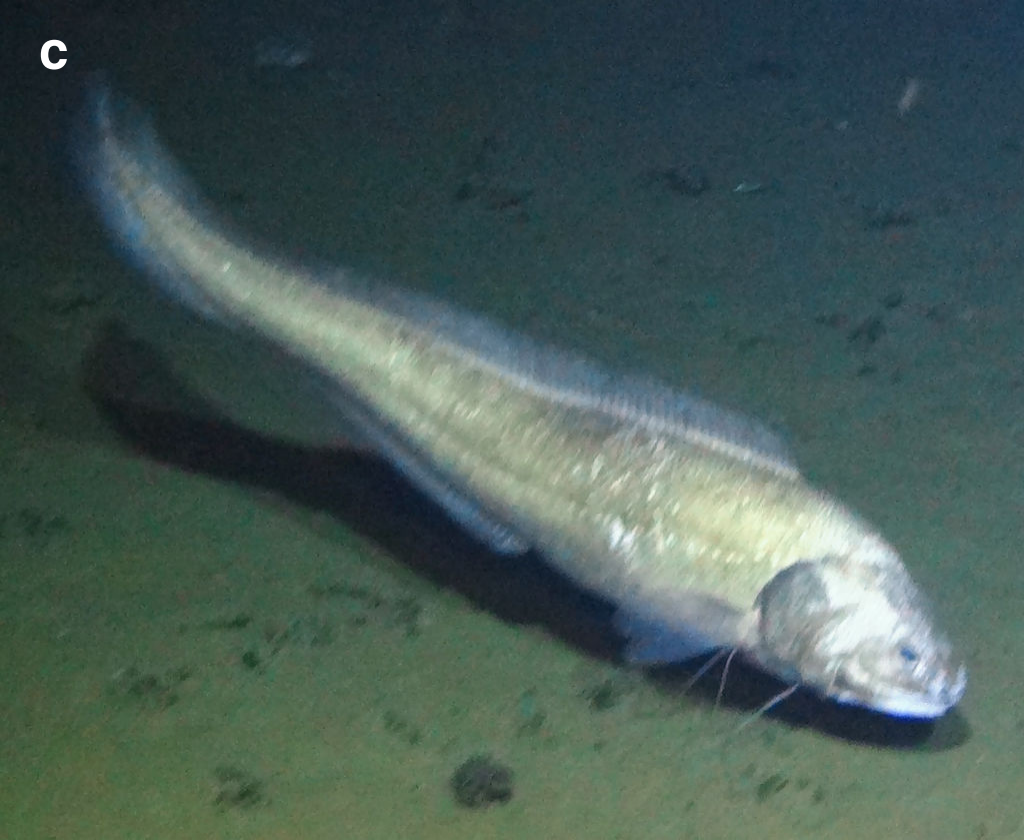
Bathyonus cf. caudalis swimming above the seafloor. Image attribution: DJ Amon & CR Smith, University of Hawai’i.

**Figure 45.**
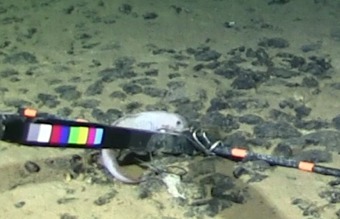
cf. Zoarcidae morphospecies 1 in situ on the seafloor in the UK-1 exploration contract area. Image corresponds with the data above. Image attribution: A Leitner & J Drazen, University of Hawai’i.

**Figure 46a.**
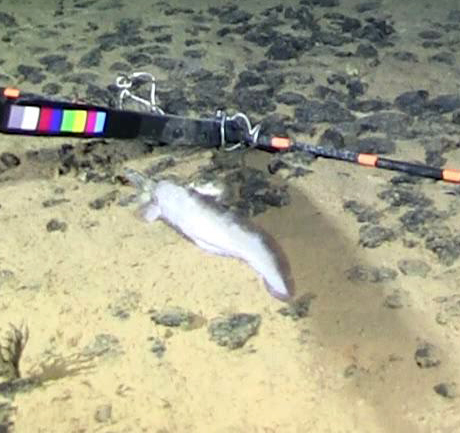
cf. Zoarcidae morphospecies 2 in situ on the seafloor. Image attribution: A Leitner & J Drazen, University of Hawai’i.

**Figure 46b.**
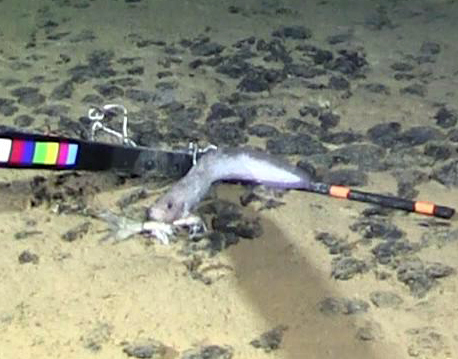
Front view of cf. Zoarcidae morphospecies 2 in situ on the seafloor. Image attribution: A Leitner & J Drazen, University of Hawai’i.

**Figure 47a.**
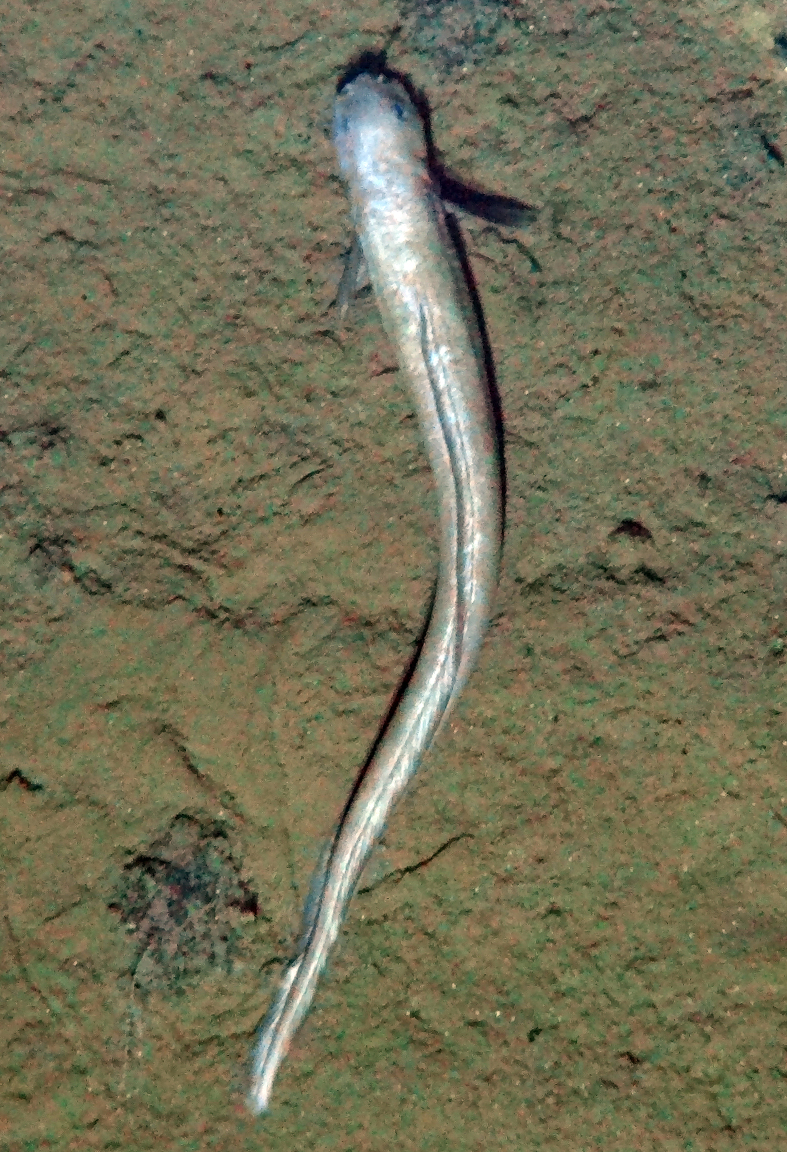
Pachycara cf. nazca in situ on the seafloor. Image attribution: DJ Amon & CR Smith, University of Hawai’i.

**Figure 47b.**
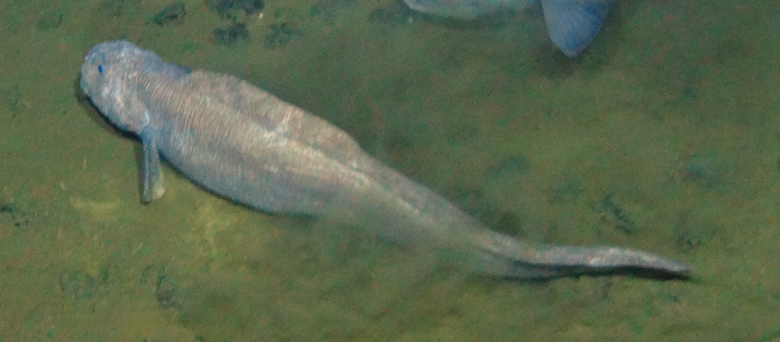
Pachycara cf. nazca in situ on the seafloor. Image attribution: DJ Amon & CR Smith, University of Hawai’i.

**Figure 47c.**
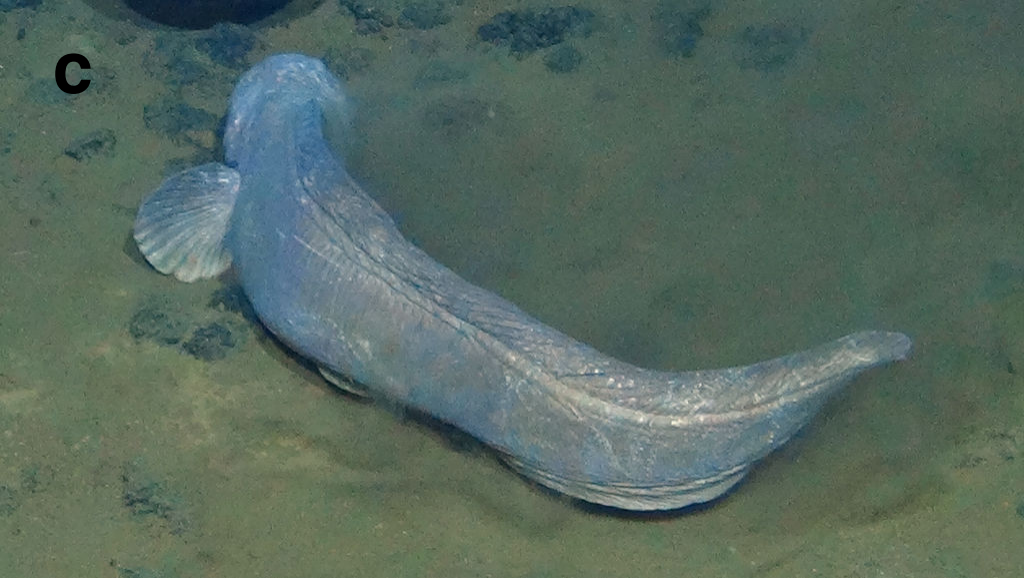
Pachycara cf. nazca in situ on the seafloor. Image attribution: DJ Amon & CR Smith, University of Hawai’i.

**Figure 47d.**
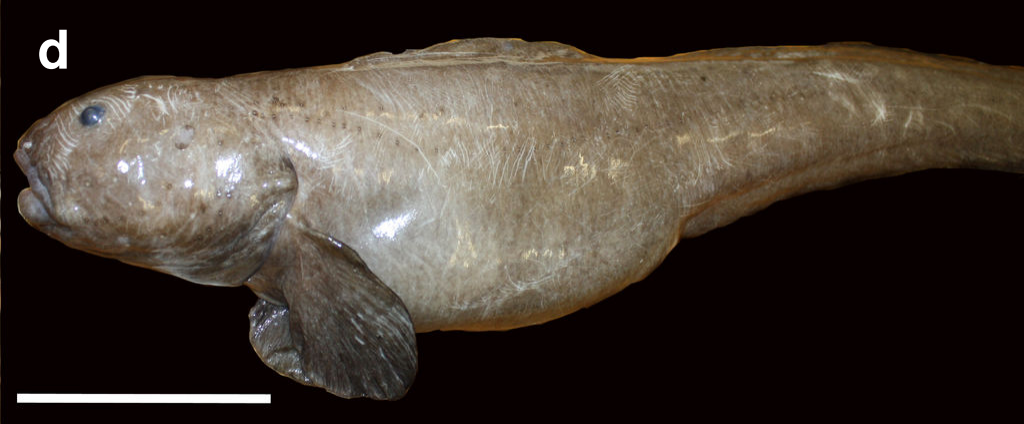
*Pachycara nazca* after collection via baited trap from the UK-1 exploration contract area. Image attribution: A Leitner & J Drazen, University of Hawai’i.

**Figure 47e.**
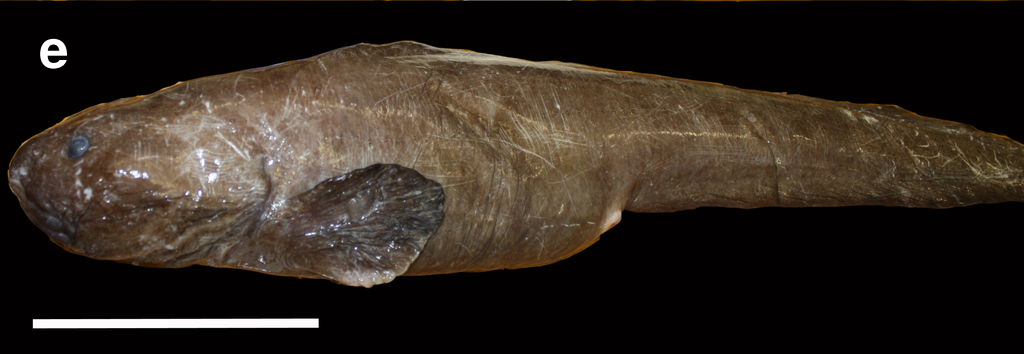
*Pachycara nazca* after collection via baited trap from the UK-1 exploration contract area. Image attribution: A Leitner & J Drazen, University of Hawai’i.

**Figure 47f.**
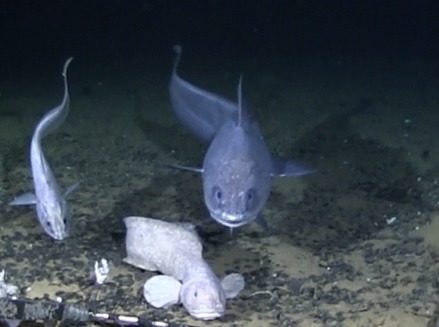
Pachycara cf. nazca (middle) in situ on the seafloor with two *Coryphaenoides* fish swimming above. Image attribution: A Leitner & J Drazen, University of Hawai’i.

**Figure 48.**
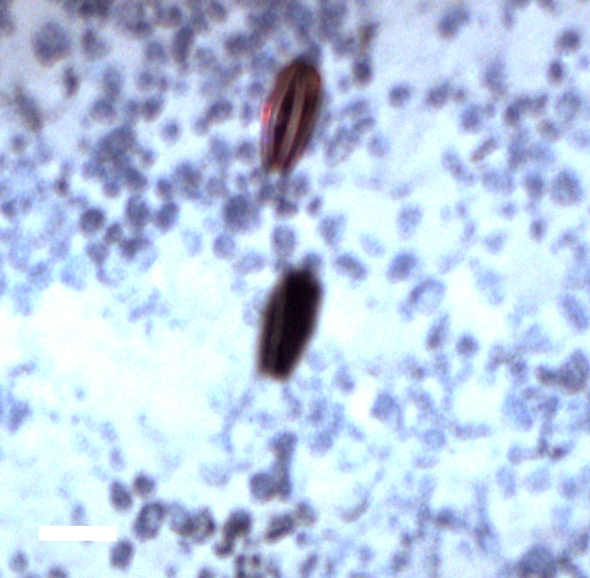
cf. Mertensiidae morphospecies observed swimming above the seafloor in the UK-1 exploration contract area. Image corresponds with the data above. Scale bar is 10 cm. Image attribution: Woods Hole Oceanographic Institution.

**Figure 49.**
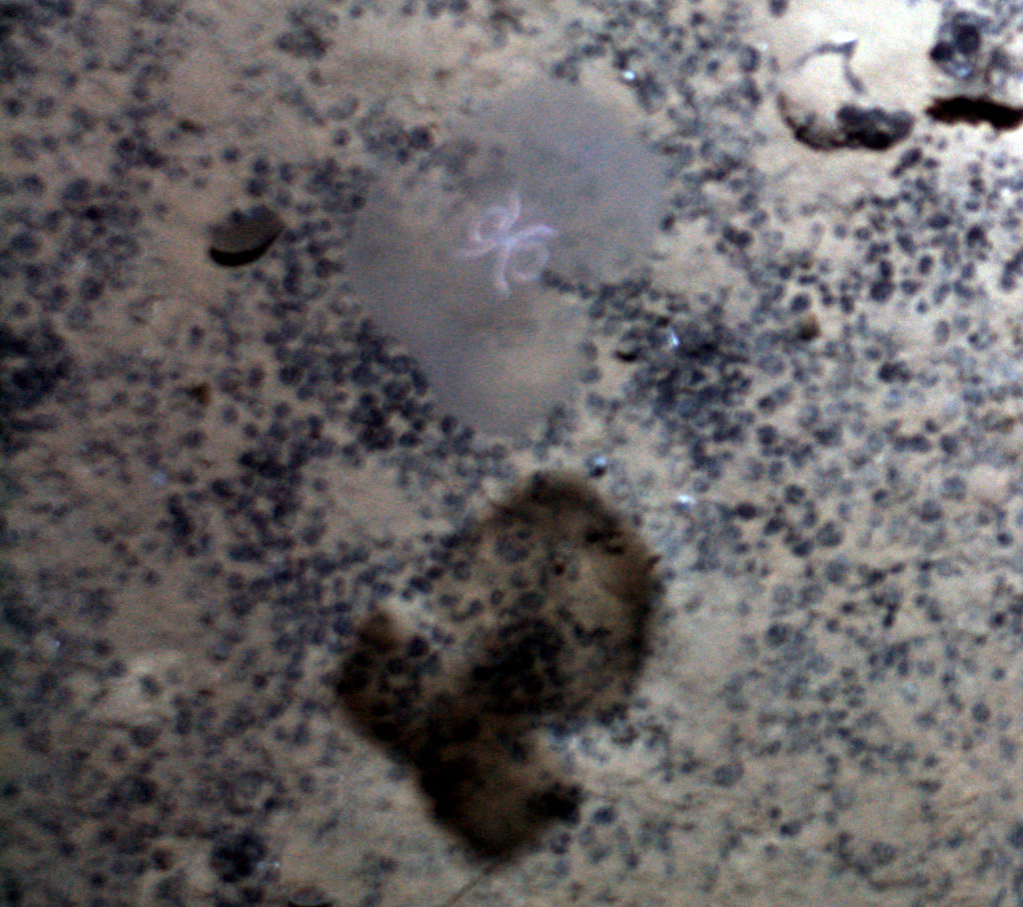
cf. Lobata morphospecies 1 observed swimming above the seafloor in the UK-1 exploration contract area. Image corresponds with the data above. Image attribution: Woods Hole Oceanographic Institution.

**Figure 50.**
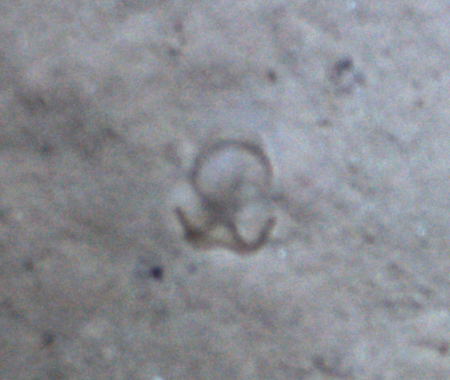
cf. Lobata morphospecies 2 observed swimming above the seafloor in the UK-1 exploration contract area. Image corresponds with the data above. Image attribution: Woods Hole Oceanographic Institution.

**Figure 51a.**
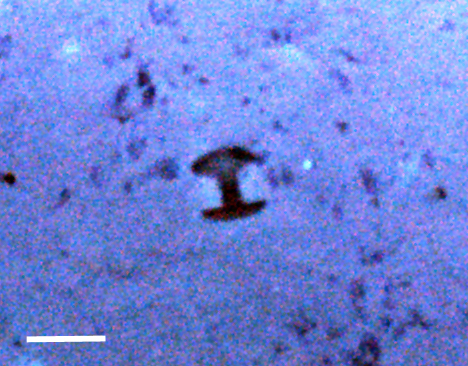
cf. Lobata morphospecies 3 swimming above the seafloor. Scale bar is 10 cm. Image attribution: Woods Hole Oceanographic Institution.

**Figure 51b.**
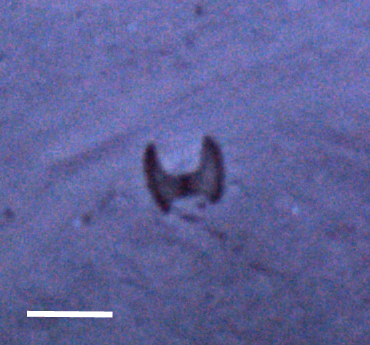
cf. Lobata morphospecies 3 swimming above the seafloor. Scale bar is 10 cm. Image attribution: Woods Hole Oceanographic Institution.

**Figure 51c.**
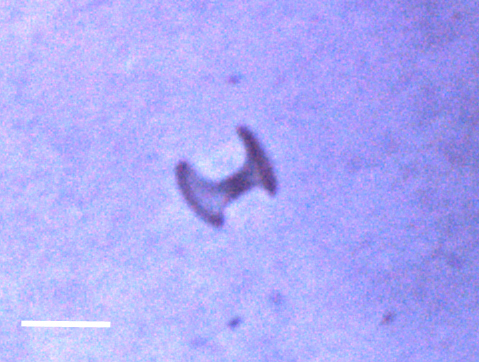
cf. Lobata morphospecies 3 swimming above the seafloor. Scale bar is 10 cm. Image attribution: Woods Hole Oceanographic Institution.

**Figure 51d.**
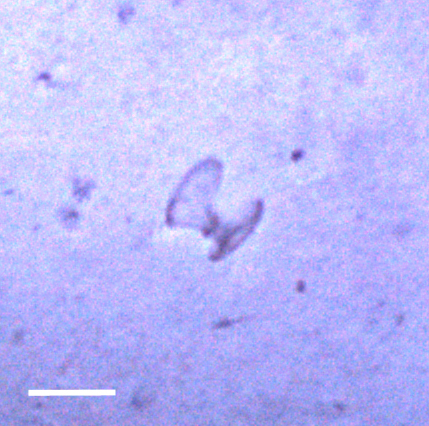
cf. Lobata morphospecies 3 swimming above the seafloor. Scale bar is 10 cm. Image attribution: Woods Hole Oceanographic Institution.

**Figure 52.**
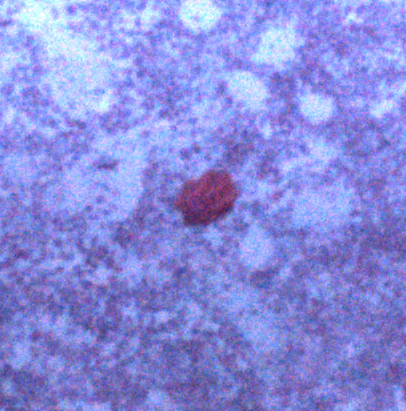
Lampocteis cf. cruentiventer observed swimming above the seafloor in the UK-1 exploration contract area. Image corresponds with the data above. Image attribution: Woods Hole Oceanographic Institution.

**Figure 53a.**
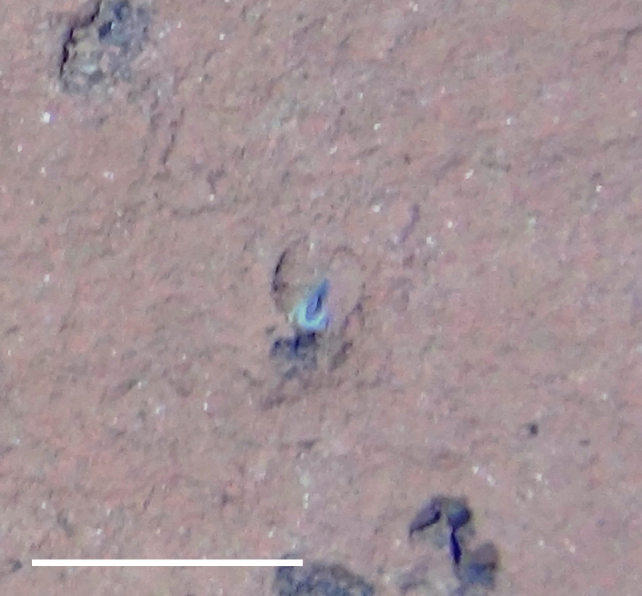
cf. Bivalvia morphospecies in situ on the seafloor. Scale bar is 10 cm. Image attribution: DJ Amon & CR Smith, University of Hawai’i.

**Figure 53b.**
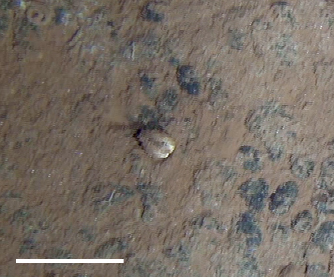
cf. Bivalvia morphospecies in situ on the seafloor. Scale bar is 10 cm. Image attribution: DJ Amon & CR Smith, University of Hawai’i.

**Figure 53c.**
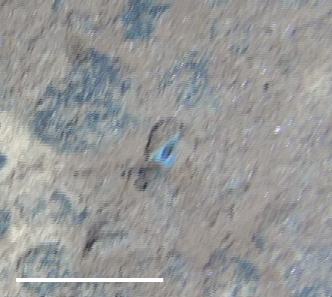
cf. Bivalvia morphospecies in situ on the seafloor. Scale bar is 10 cm. Image attribution: DJ Amon & CR Smith, University of Hawai’i.

**Figure 54a.**
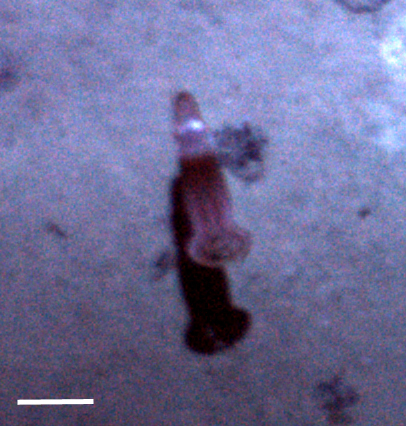
cf. Cirroteuthidae morphospecies swimming above the seafloor. Scale bar is 10 cm. Image attribution: Woods Hole Oceanographic Institution.

**Figure 54b.**
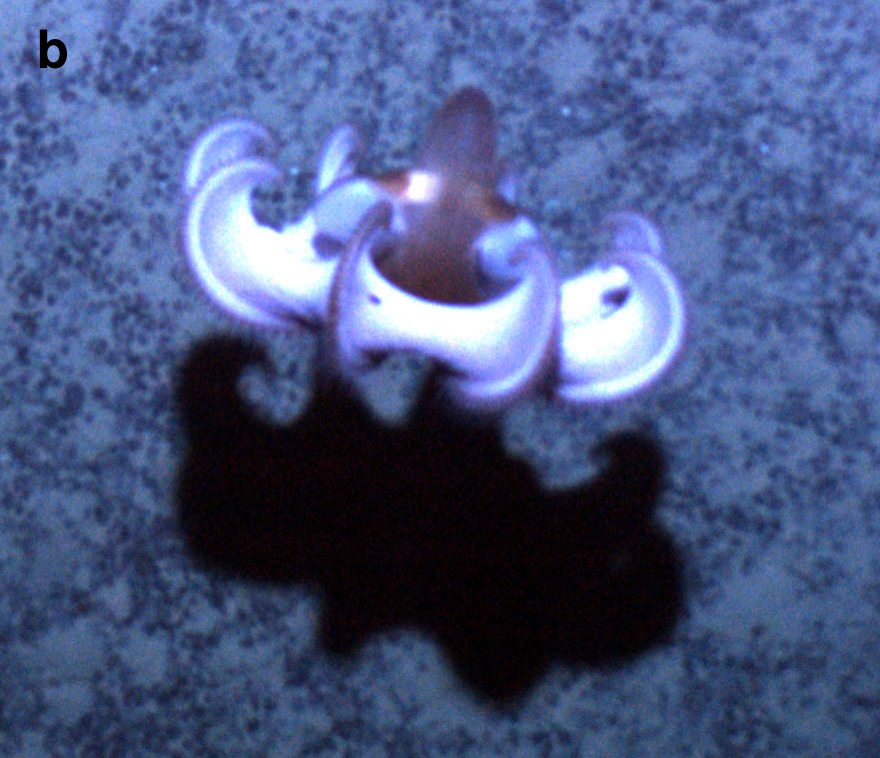
cf. Cirroteuthidae morphospecies swimming above the seafloor. Image attribution: Woods Hole Oceanographic Institution.

**Figure 54c.**
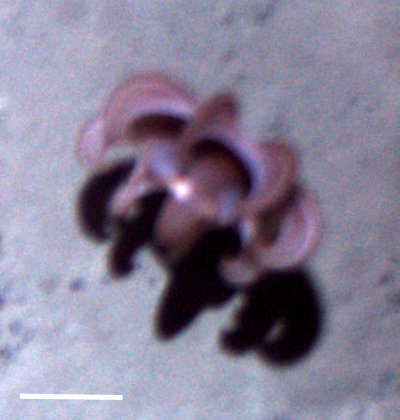
cf. Cirroteuthidae morphospecies swimming above the seafloor. Scale bar is 10 cm. Image attribution: Woods Hole Oceanographic Institution.

**Figure 54d.**
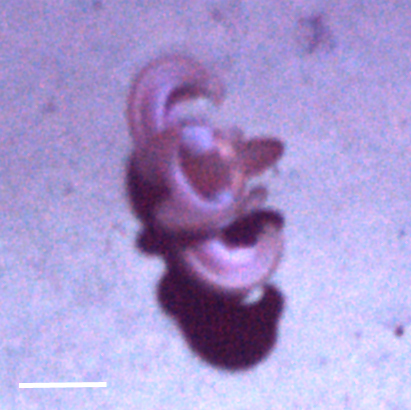
cf. Cirroteuthidae morphospecies swimming above the seafloor. Scale bar is 10 cm. Image attribution: Woods Hole Oceanographic Institution.

**Figure 55a.**
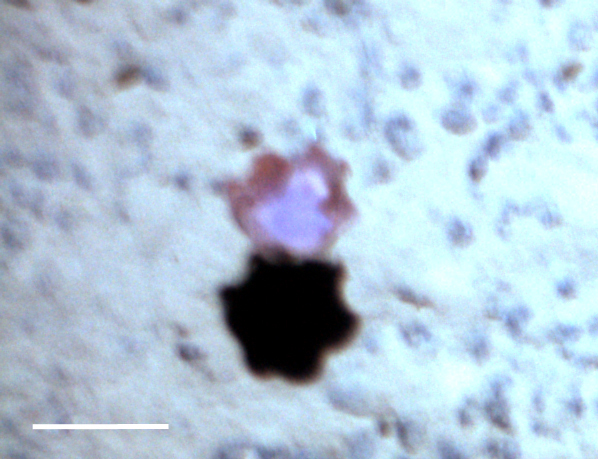
cf. *Grimpoteuthis* morphospecies swimming above the seafloor. Scale bar is 10 cm. Image attribution: Woods Hole Oceanographic Institution.

**Figure 55b.**
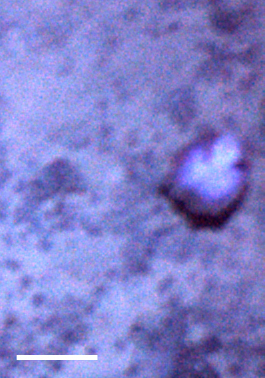
cf. *Grimpoteuthis* morphospecies swimming above the seafloor. Scale bar is 10 cm. Image attribution: Woods Hole Oceanographic Institution.

**Figure 55c.**
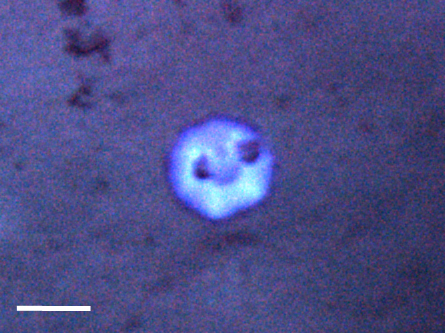
cf. *Grimpoteuthis* morphospecies in situ on the seafloor. Scale bar is 10 cm. Image attribution: Woods Hole Oceanographic Institution.

**Figure 55d.**
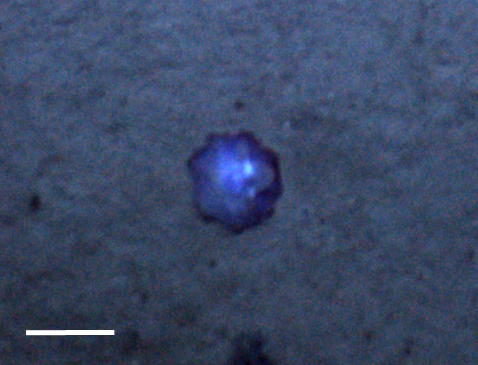
cf. *Grimpoteuthis* morphospecies in situ on the seafloor. Scale bar is 10 cm. Image attribution: Woods Hole Oceanographic Institution.

**Figure 56.**
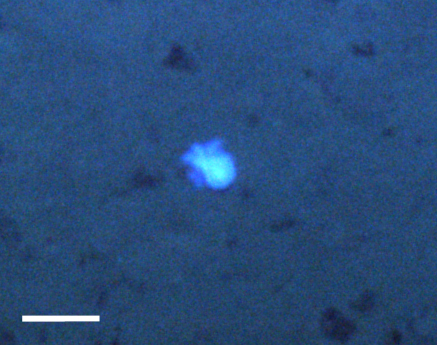
cf. *Muusoctopus* morphospecies observed on the seafloor in the UK-1 exploration contract area. Image corresponds with the data above. Scale bar is 10 cm. Image attribution: Woods Hole Oceanographic Institution.
